# Epigenetic regulation during cancer transitions across 11 tumour types

**DOI:** 10.1038/s41586-023-06682-5

**Published:** 2023-11-01

**Authors:** Nadezhda V. Terekhanova, Alla Karpova, Wen-Wei Liang, Alexander Strzalkowski, Siqi Chen, Yize Li, Austin N. Southard-Smith, Michael D. Iglesia, Michael C. Wendl, Reyka G. Jayasinghe, Jingxian Liu, Yizhe Song, Song Cao, Andrew Houston, Xiuting Liu, Matthew A. Wyczalkowski, Rita Jui-Hsien Lu, Wagma Caravan, Andrew Shinkle, Nataly Naser Al Deen, John M. Herndon, Jacqueline Mudd, Cong Ma, Hirak Sarkar, Kazuhito Sato, Omar M. Ibrahim, Chia-Kuei Mo, Sara E. Chasnoff, Eduard Porta-Pardo, Jason M. Held, Russell Pachynski, Julie K. Schwarz, William E. Gillanders, Albert H. Kim, Ravi Vij, John F. DiPersio, Sidharth V. Puram, Milan G. Chheda, Katherine C. Fuh, David G. DeNardo, Ryan C. Fields, Feng Chen, Benjamin J. Raphael, Li Ding

**Affiliations:** 1 Department of Medicine, Washington University in St Louis, St Louis, MO USA; 2 McDonnell Genome Institute, Washington University in St Louis, St Louis, MO USA; 3 Department of Computer Science, Princeton University, Princeton, NJ USA; 4 Department of Surgery, Washington University in St Louis, St Louis, MO USA; 5 Siteman Cancer Center, Washington University in St Louis, St Louis, MO USA; 6 Josep Carreras Leukaemia Research Institute, Barcelona, Spain; 7 Barcelona Supercomputing Center, Barcelona, Spain; 8 Department of Radiation Oncology, Washington University in St Louis, St Louis, MO USA; 9 Department of Neurological Surgery, Washington University in St Louis, St Louis, MO USA; 10 Department of Otolaryngology—Head & Neck Surgery, Washington University in St Louis, St Louis, MO USA; 11 Department of Obstetrics and Gynecology, University of California, San Francisco, San Francisco, CA USA; 12 Department of Obstetrics and Gynecology, Washington University in St Louis, St Louis, MO USA; 13 Department of Genetics, Washington University in St Louis, St Louis, MO USA

**Keywords:** Cancer genomics, Cell biology, Computational biology and bioinformatics

## Abstract

Chromatin accessibility is essential in regulating gene expression and cellular identity, and alterations in accessibility have been implicated in driving cancer initiation, progression and metastasis^1–4^. Although the genetic contributions to oncogenic transitions have been investigated, epigenetic drivers remain less understood. Here we constructed a pan-cancer epigenetic and transcriptomic atlas using single-nucleus chromatin accessibility data (using single-nucleus assay for transposase-accessible chromatin) from 225 samples and matched single-cell or single-nucleus RNA-sequencing expression data from 206 samples. With over 1 million cells from each platform analysed through the enrichment of accessible chromatin regions, transcription factor motifs and regulons, we identified epigenetic drivers associated with cancer transitions. Some epigenetic drivers appeared in multiple cancers (for example, regulatory regions of *ABCC1* and *VEGFA*; GATA6 and FOX-family motifs), whereas others were cancer specific (for example, regulatory regions of *FGF19*, *ASAP2* and *EN1*, and the PBX3 motif). Among epigenetically altered pathways, TP53, hypoxia and TNF signalling were linked to cancer initiation, whereas oestrogen response, epithelial–mesenchymal transition and apical junction were tied to metastatic transition. Furthermore, we revealed a marked correlation between enhancer accessibility and gene expression and uncovered cooperation between epigenetic and genetic drivers. This atlas provides a foundation for further investigation of epigenetic dynamics in cancer transitions.

## Abstract (web summary)

A pan-cancer epigenetic and transcriptomic atlas identifies epigenetic drivers associated with cancer transitions.

## Main

The spatiotemporal dynamics of chromatin decondensation and subsequent binding of transcriptional machinery^1^ has an important but incompletely understood role in pathogenic transitions in cancer, such as initiation, progression and metastasis^2^. Epigenetic regulation affects gene expression, lineage determination, cell–cell interactions and therapeutic resistance. In contrast to genetic drivers, such as somatic mutations, epigenetic drivers are less well defined^3^. However, they might be identified by an enrichment type of analysis, similar to how driver genes are found. Understanding their interactions with genetic and environmental factors is also crucial. An interaction was recently demonstrated^4^ involving *KRAS* mutation and tissue damage in the pancreatic epithelium that remodels chromatin, producing cancer-favouring transcriptional activity. Here we consider epigenetic drivers to be the activity of regulatory elements or transcription factors (TFs) associated with cancer initiation, progression and metastasis, often through interactions with genetic drivers. It is possible that such epigenetic drivers may explain previously unknown tumorigenic mechanisms.

The assay for transposase-accessible chromatin using sequencing (ATAC-seq) is a rapid and sensitive method for profiling the epigenome^5^. Previous studies have obtained ATAC-seq results for some cancers^6,7^ at the bulk level as averages across different cell types within a tumour. The recent development of single-nucleus ATAC-seq (snATAC-seq) provides a far greater resolution to examine single-cell epigenomes^8,9^. Coupling snATAC-seq with single-nucleus RNA sequencing (snRNA-seq) permits simultaneous profiling of the epigenome and transcriptome in the same individual cells, enabling direct analysis of associations between chromatin accessibility and gene transcription. We constructed an integrative multi-omic atlas of 11 major cancer types procured from over 200 patient cases. The large number of samples and considerable representation of cancer types and statuses (for example, normal, primary and metastatic) furnish a well-powered cohort for investigating epigenetic drivers in cancer.

We provide a unified map of lineage-specific and cancer-specific cell populations, differentially accessible enhancers and promoters, epigenetically regulated cancer-associated genes and TFs that are important across major cancer transitions. Although some of these drivers and transcriptional programs are associated with transitions in multiple cancer types, others show high cancer-type specificity. We found that correlations between epigenetic changes and genetic mutations within the same pathway are present across cancer types, suggesting numerous instances of cooperation in cancer transition programs. This study highlights the potential of TFs as prognostic markers, offering a deeper understanding of the molecular underpinnings driving cancer evolution.

## Chromatin accessibility across cancers

**Fig. 1:**
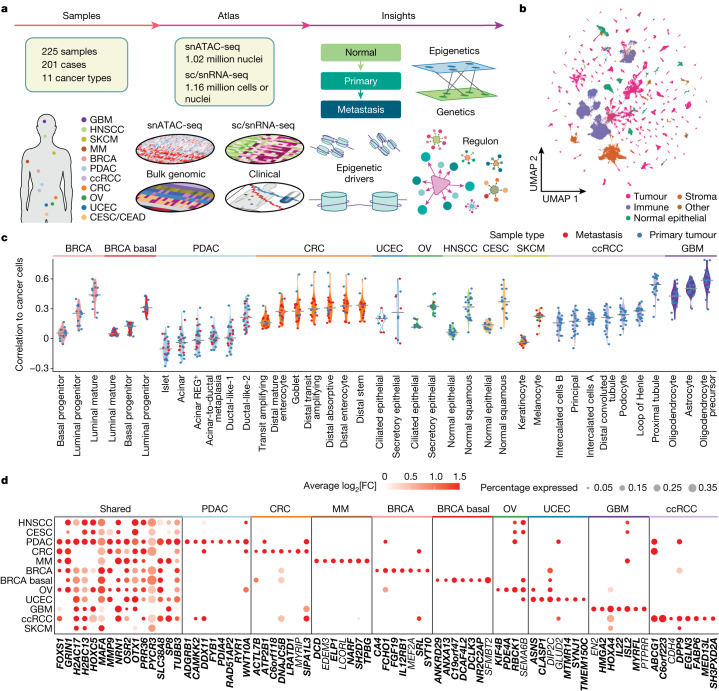
Chromatin accessibility patterns across 11 cancer types. **a**, Schematic of the data generation and study design, showing the cancer types and sample types collected, the building, annotation and integration of the atlas, and the biological entities that were investigated. **b**, Uniform manifold approximation and projection (UMAP) plot of an integrated pan-cancer snATAC-seq object showing the distribution of 250,222 immune, 69,684 stromal, 69,506 normal epithelial and 588,895 cancer cells across 225 samples. A detailed breakdown of 36 different cell types is shown in Extended Data Figs. 1b and 2a,b. **c**, The Pearson’s correlation coefficients between cancer cells from each tumour and normal cell types of the tumour’s tissue of origin. Cell types are ordered by increasing median correlation coefficient per cohort; the right-most cell type was considered the CNC and was subsequently used as a reference for identifying cancer-associated epigenetic drivers. **d**, The top cancer-cell-associated DACRs identified by comparing cancer cells versus the CNCs. The bubble size shows the percentage of cancer cells with accessible DACRs and the colour conveys the log_2_[FC]. The *x* axis shows the nearest gene of each DACR. Genes are grouped by those shared between cancers and those specific to cancer types. Cancer-specific DACRs were selected on the basis of specificity and by fold change (FC) in each cancer type (columns), or if they were shared by the maximal number of cancers (shared). Positive log_2_[FC] is shown if ACR was accessible in >5% of cancer cells. DACRs of genes that overlap promoters and enhancers from the EpiMap database are highlighted in bold.

**Extended Data Fig. 1:**
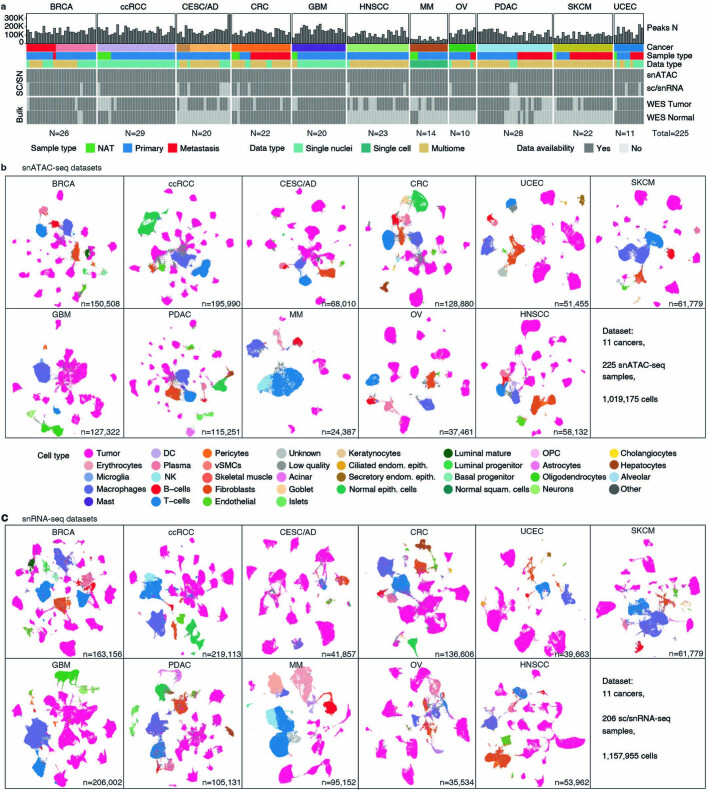
snATAC-seq and sc/snRNA-seq data overview. **a**, Overview of the cohort and sample availability, indicating 11 different cancer types, 3 sample types (NAT - normal adjacent tissue, Primary - primary tumour, Metastasis - metastatic tumour), and 3 data types collected for each sample. The bar plot annotation on top of the heatmap provides information about the number of peaks detected in each sample. **b**, UMAPs of 11 cancer types based on snATAC-seq chromatin accessibility. Each cell is colour-coded by cell type to visualize the differences in chromatin accessibility. **c**, UMAPs of 11 cancer types based on sc/snRNA-seq data. Each cell is colour-coded by cell type to visualize the differences in gene expression.

**Extended Data Fig. 2:**
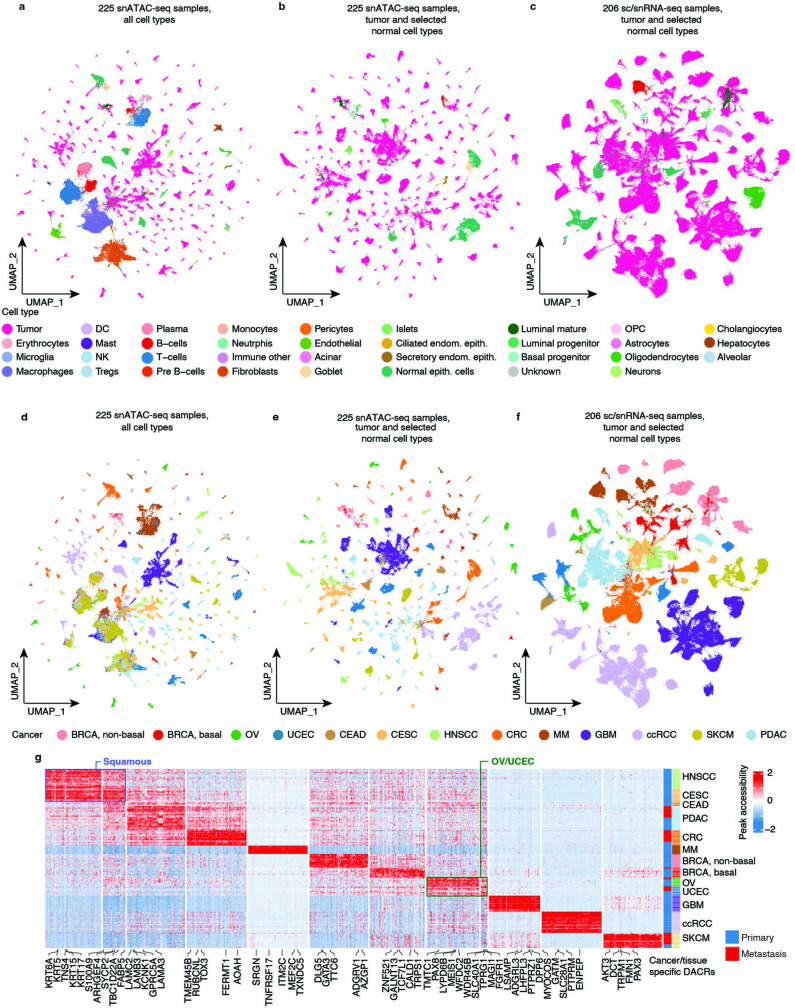
Integrated data overview and DACRs between cancer cells. **a-c**, UMAPs of pan-cancer objects: (**a**) 225 snATAC-seq samples, all cell types; (**b**) 225 snATAC-seq samples, tumour and selected normal cell types; (**c**) 206 sc/snRNA-seq samples, tumour and selected normal cell types. Cells are coloured by cell type. **d-f**, UMAPs of pan-cancer objects: (**d**) 225 snATAC-seq samples, all cell types; (**e**) 225 snATAC-seq samples, tumour and selected normal cell types; (**f**) 206 sc/snRNA-seq samples, tumour and selected normal cell types. Cells are coloured by cancer type. **g**, Tissue- and cancer cell-specific differentially accessible chromatin regions (DACRs) identified across 11 cancer types. Columns correspond to DACRs and rows to tumour samples of every cancer type. For each sample peak accessibility was calculated as average across its cancer cells. Labels on the abscissa indicate DACRs that are in promoters of the top 5 tissue- and cancer cell-specific DEGs by fold change. DACRs that are shared between CESC/HNSCC and UCEC/OV are highlighted.

As part of the NCI Human Tumour Atlas Network (HTAN), we procured 225 samples from 158 primary and 52 metastatic tumour samples and 15 normal adjacent tissues (NATs) from 201 patients across 11 cancer types (Fig. 1a,b, Extended Data Figs. 1 and 2 and Supplementary Table 1a,b). This set contains 52 metastatic samples from colorectal cancer (CRC), pancreatic ductal adenocarcinoma (PDAC), skin cutaneous melanoma (SKCM), uterine corpus endometrial carcinoma (UCEC), ovarian cancer (OV) and breast cancer (BRCA), including paired primary and metastatic samples of five cases of UCEC and four cases of CRC. We performed snATAC-seq analysis of all 225 samples, along with paired single-cell or single-nucleus RNA-seq (sc/snRNA-seq) for 206 of these samples (Supplementary Table 1a). Among those, 14 scRNA-seq multiple myeloma (MM) samples, 10 snRNA-seq PDAC samples, 14 snRNA-seq glioblastoma (GBM) samples, and 28 snATAC-seq and 27 snRNA-seq clear-cell renal cell carcinoma (ccRCC) samples were obtained from previous studies^10–13^, with many of the ccRCC and GBM samples originating from the NCI Clinical Proteomic Tumour Analysis Consortium (CPTAC). Bulk whole-exome sequencing (WES) data were also generated for 195 samples (Supplementary Table 1a). These multi-omic datasets enable the systematic discernment of cell subpopulations in diverse cancer types and tracing of cancer transitions from normal precursor to primary tumour to metastasis.

The snATAC-seq data encompassed 1,019,175 nuclei from the 225 samples (mean nuclei per sample, 4,530) (Fig. 1b and Extended Data Fig. 1b). We identified accessible chromatin regions (ACRs) across all samples, averaging 126,196 ACRs per sample, with most appearing in intronic (49%), distal intergenic (30.8%) and promoter (8.6%) regions, as expected (Supplementary Table 1c,d and Supplementary Note 1). We also performed sc/snRNA-seq analysis of 206 samples, with snRNA-seq and snATAC-seq data generated from the same cells in 129 instances (snMultiome-seq samples; Extended Data Fig. 1a). The combined sc/snRNA-seq data yielded 1,157,955 cells or nuclei, which were annotated by the expression of curated epithelial, immune and stromal marker genes (Extended Data Fig. 1c, Supplementary Note 2 and Supplementary Table 1e). sc/snRNA-seq cell annotation was further used to annotate the snATAC-seq dataset. In total, 250,222 immune, 69,684 stromal, 69,506 normal epithelial and 588,895 cancer cells were detected (Fig. 1b and Extended Data Fig. 1b).

**Extended Data Fig. 3:**
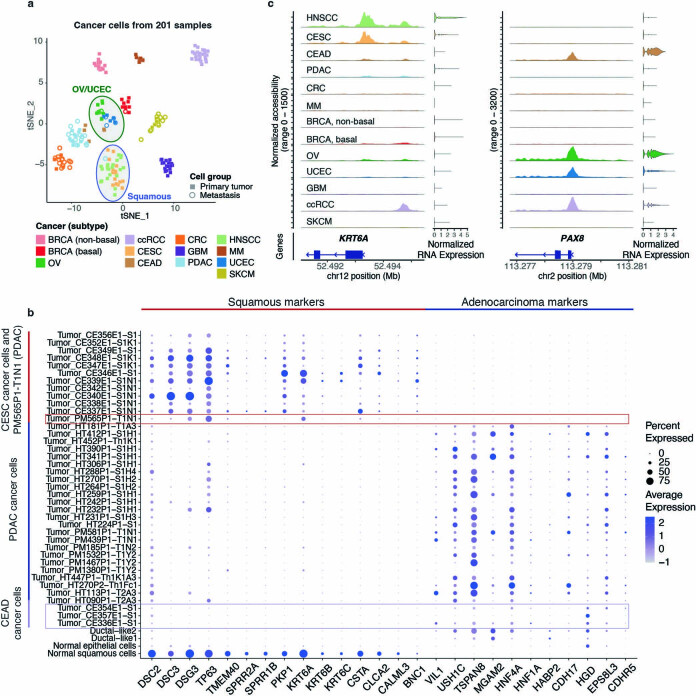
Similarity patterns between squamous cancers, and OV and UCEC. **a**, tSNE-plot based on top tissue- and cancer cell-specific DACRs from Extended Data Fig. 2g showing tumour samples clustering. CESC/HNSCC and UCEC/OV clusters of samples are highlighted with shading. **b**, Dot plots showing squamous and adenocarcinoma markers snRNA-seq expression in CESC/AD and PDAC samples. Markers were obtained from^101^. **c**, snATAC-seq based coverage plots showing examples of pan-cancer ACRs: *KRT6A* (left) is shared between CESC and HNSCC, and *PAX8* (right) is shared among UCEC, OV, CEAD, and ccRCC cancer types.

We identified 56,001 tissue- and cancer-cell-specific differentially accessible chromatin regions (DACRs) by comparing each cancer type to all others (Extended Data Fig. 2g, Supplementary Table 2a–c and Supplementary Note 3). Many of these DACRs include the promoters of tissue-specific genes, such as keratin genes in squamous cancers, *PAX8* in OV and UCEC, *GATA3* in non-basal BRCA, *PTPRZ1* in GBM and *PAX3* in SKCM (Extended Data Fig. 2g). Dimensionality reduction of chromatin accessibility in malignant cells at the sample level (Extended Data Fig. 3a) revealed the expected similarity between cancer types that reflect their primary tissue of origin. Specifically, head and neck squamous cell carcinoma (HNSCC) and cervical squamous cell carcinoma (CESC) were co-clustered, whereas non-squamous cervical samples were clustered with epithelial cancer types, further supported by high expression of adenocarcinoma markers (therefore annotated as CEAD) (Extended Data Fig. 3b). We also found that one PDAC metastatic sample had high expression of squamous markers, but lacked adenocarcinoma markers, explaining its co-clustering with other squamous cancers (Extended Data Fig. 3a). We also observed that UCEC and OV clustered together, whereas basal BRCA was distinctly separated from non-basal BRCA, indicating significant differences between these subtypes. These were subsequently treated as separate cancer types here. One example of a squamous-tissue-specific ACR shared by the squamous cancers HNSCC and CESC was the *KRT6A* promoter region (Extended Data Fig. 3c (left)). *KRT6A* encodes keratin 6A, which is an important biomarker of the squamous lineage^14^. The similarity between OV and UCEC cancer cells was exemplified by shared accessibility of the *PAX8* promoter (Extended Data Fig. 3c (right)), which is consistent with its known association with these cancers^15,16^.

## Chromatin regions altered in tumours

**Extended Data Fig. 4:**
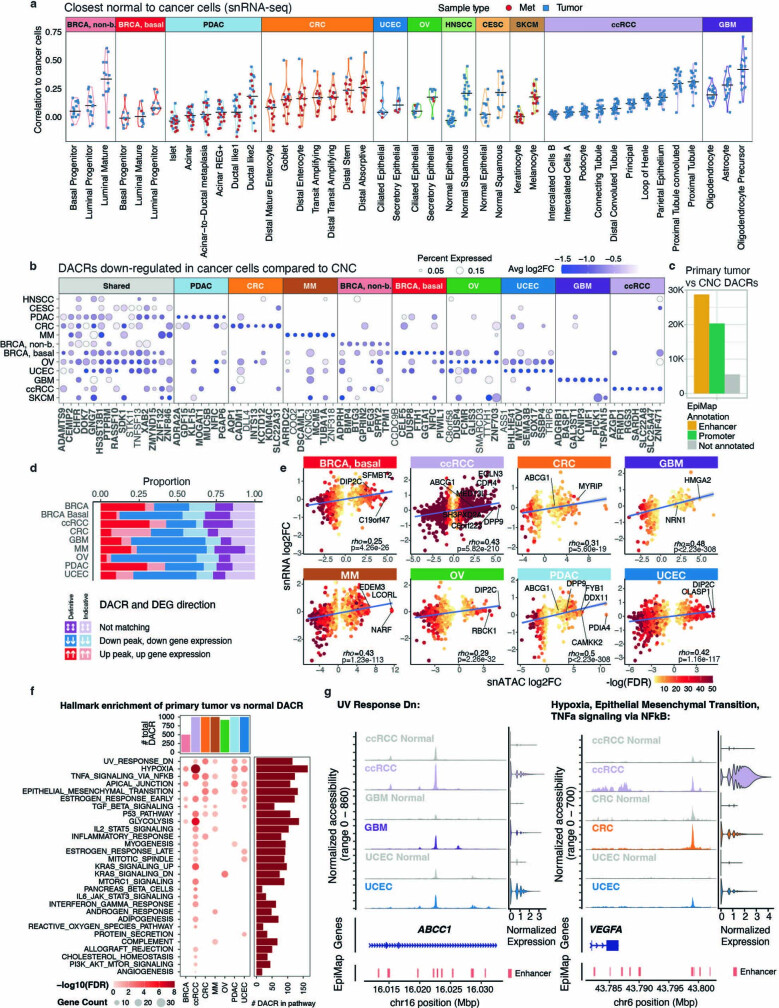
Characterization of cancer cell-specific DACRs. **a**, Violin plots showing distributions of Pearson correlation coefficients between cancer cells from each tumour and normal cell types of tissue of origin using snRNA-seq data. For each cohort, cell types are ordered by increasing median correlation coefficient across samples. **b**, Bubble plot showing top down-regulated cancer cell-associated DACRs, including shared and cancer-specific DACRs, identified by comparing cancer cells vs. CNC. Bubble size shows percentage of cancer cells with accessible DACR and colour conveys log2 fold change (FC). X-axis shows the nearest gene of each DACRs. Genes are grouped by those shared between cancers and those specific to cancer types. Cancer-specific DACRs were selected based on specificity and by FC in each cancer type (columns), or if they were shared by maximal number of cancers (shared). Negative log2(FC) is shown if ACR was accessible in >0.05 of cancer cells. Genes’ DACRs that overlap promoters and enhancers from the EpiMap database are highlighted in bold. **c**, Bar chart shows counts of primary cancer cells vs. CNCs DACRs broken down by EpiMap annotation. 53% of DACRs are annotated as enhancer regions and 37% as promoter regions, the rest are not annotated in EpiMap. **d**, Bar plot showing the proportion of primary cancer cells vs. CNCs DACRs (FDR < 0.05) for which the nearest gene definitively changes expression in the same direction (includes significant RNA hits at FDR < 0.05 and suggestive RNA hits at FDR < 0.3, absolute log2FC > 0.05), or gene indicatively changes expression in the same direction (absolute log2FC < 0.05). DACRs and nearby genes that do not match in the direction of accessibility/expression are marked as ‘not matching’. **e**, Scatter plots showing correlation of log2FC of DACRs and log2FC of DEGs of nearby genes. Spearman’s rho values and two-sided *p-*values are shown. Dot colour indicates -log10(FDR). The grey band corresponds to the 95% confidence level interval for predictions from the linear model. **f**, Bubble plot showing significant and suggestive (FDR ≤ 20%) hallmark pathway enrichments from upregulated cancer cell-specific DACRs in panel Fig. 1d. Bubble size and colour convey gene count and log10 of FDR, respectively. The total number of DACRs per cancer type in the analysis is capped at 1,000 by log2 fold change to ensure balanced comparison (top bar plot). Total number of DACRs annotated in each hallmark pathway are shown on the bar plot on the right. **g**, Coverage plots showing chromatin accessibility in genomic regions containing genes *ABCC1* and *VEGFA* for neoplastic cells and CNC in each cancer type. DACRs are highlighted and gene expression levels show concordance with chromatin accessibility (right).

We sought to define the genetic and epigenetic changes underpinning the transition from normal cells to cancer cells. By correlating cancer cells and normal cells on the basis of chromatin accessibility (Fig. 1c) and gene expression (Extended Data Fig. 4a), we defined the following normal cell populations as the closest normal cells (CNCs): luminal mature cells for BRCA of the non-basal subtypes; luminal progenitor cells for BRCA of the basal subtype; ductal-like-2 cells for PDAC; distal stem cells for CRC; secretory endometrial epithelial cells for UCEC and OV; normal squamous cells for HNSCC and CESC; melanocytes for SKCM; proximal tubule cells for ccRCC; and oligodendrocyte precursor cells (OPCs) for GBM. The CNCs that we identified in this manner are consistent with those identified in previous studies (Methods and Supplementary Table 2d). For MM, we used normal B cells as the CNC^17^.

We used these CNCs to remove tissue-specific signals and identify cancer-cell-specific changes in chromatin accessibility shared by several cancer types. By comparing cancer cells with their respective CNCs, we found 22,187 (Fig. 1d) and 29,074 (Extended Data Fig. 4b) respective regions of increased and decreased accessibility in cancer cells and mapped them to their nearest respective genes based on DACR proximity to the closest transcription start sites (TSS) (Supplementary Table 2e,f). In total, 53% of DACRs were found in enhancer regions and 37% in promoter regions, suggesting that their functional relevance to gene expression changes (Extended Data Fig. 4c). Indeed, across cancers, around 75% of DACRs matched the direction of the expression change of the nearest gene (Extended Data Fig. 4d). Furthermore, we performed a correlation analysis between DACRs and the nearest gene expression and observed significant positive correlation in all cancers, with the rho values ranging from 0.25 (BRCA, basal) to 0.5 (PDAC) (Extended Data Fig. 4e). Several genes showed pan-cancer patterns of increased accessibility of nearby genomic regions (Fig. 1d), including solute carrier family member *SLC38A8*, AP1 family TF *MAFA* and the prognostic biomarker in several cancers, class III β-tubulin (*TUBB3*)^18^. Cancer-type-specific DACRs included HOX TF *HOXA4* in GBM, an unfavourable prognostic factor in glioma^19^, the clinically significant marker *FGF19* in BRCA (of non-basal subtypes)^20^ and hypoxia-inducible factor 3 *EGLN3*, a known pathological marker of ccRCC^21^.

We further identified hallmark pathways enriched in cancer-cell-specific DACRs (Extended Data Fig. 4f). Large numbers of DACRs marked genes downregulated in response to ultraviolet radiation in 5 out of 7 cancer types. Among these are collagen genes, growth factor receptors and MAPK/ERK kinases. The hypoxia pathway was enriched in ccRCC, BRCA, PDAC and UCEC, as was TNF signalling in ccRCC, CRC, MM and PDAC. Although *VHL* mutation drives hypoxia in ccRCC^22^, hypoxia enrichment in other cancers cannot be explained solely by mutations, suggesting that epigenetic dysregulation is a driver. Two DACRs were especially notable (Extended Data Fig. 4g). Enhancer accessibility of *ABCC1* was increased in ccRCC, GBM and UCEC, exemplifying genes downregulated in response to ultraviolet radiation. *ABCC1* encodes multidrug-resistance protein-1 (MRP1) and promotes tumour growth through drug efflux in neuroblastoma cells^23^ and lipid-signalling pathways in uterine leiomyoma cells^24^. Another example shows increased accessibility of the enhancer of *VEGFA*, a known pro-angiogenic factor^25^, in ccRCC, CRC and UCEC.

## ACR-to-gene links in tumour progression

**Fig. 2:**
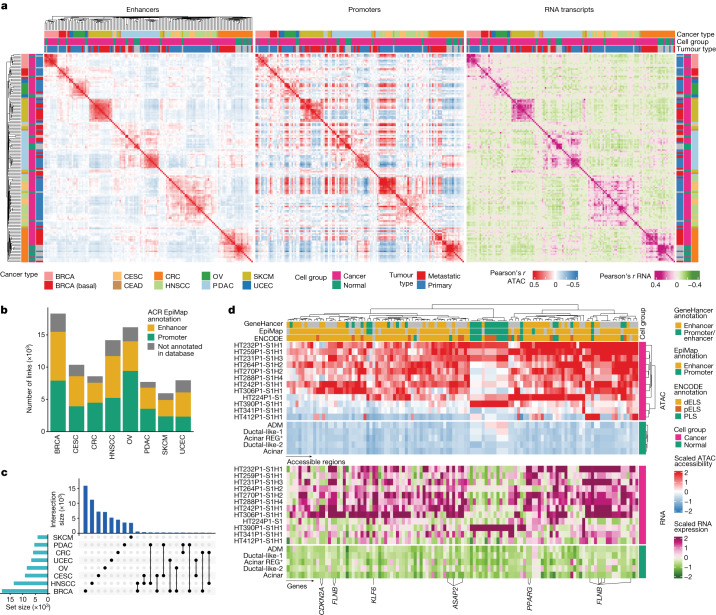
CREs regulating transcriptional programs in cancer. **a**, Sample-wise Pearson’s correlations of cancer cells and normal cells from the same tissue (snMultiome-seq samples) computed based on the accessibility of ACRs overlapping EpiMap enhancer regions (left), ACRs overlapping EpiMap promoter regions (centre) and RNA transcripts (right). The left heat map was clustered using a single-linkage clustering method and Euclidean distance, and the centre and the right heat maps follow the same order. **b**, The counts of ACR-to-gene links by cancer type and coloured by EpiMap annotation of ACRs. **c**, An UpSet plot showing that most enhancer-to-gene links are cancer-type specific. The connected dots at the bottom right indicate ACR-to-gene links shared between the cancer types denoted. **d**, The accessibility of ACRs (top) that are linked to gene expression (bottom) in PDAC cancer cells. The heat maps show the average normalized and scaled snATAC-seq and snRNA-seq values aggregated by sample for cancer cells and by cell type for normal pancreas cells. The top heat map was clustered using Ward’s minimum-variance method (Ward.D2 from R) and Euclidean distance. The bottom heat map columns and rows follow the order of the top heat map. Acinar, acinar cells; acinar REG^+^, acinar cells with high expression of regenerating proteins; ADM, acinar-to-ductal metaplasia; dELS, distal *cis-*regulatory regions (CREs) with enhancer-like signatures; ductal-like-1, ductal cells with high *SPP1* and *CRP*; ductal-like-2, ductal cells with increased mucus genes and trefoil factor genes; islets, all islets of Langerhans cells; pELS, proximal CREs with enhancer-like signatures; PLS, CREs with promoter-like signatures.

**Extended Data Fig. 5:**
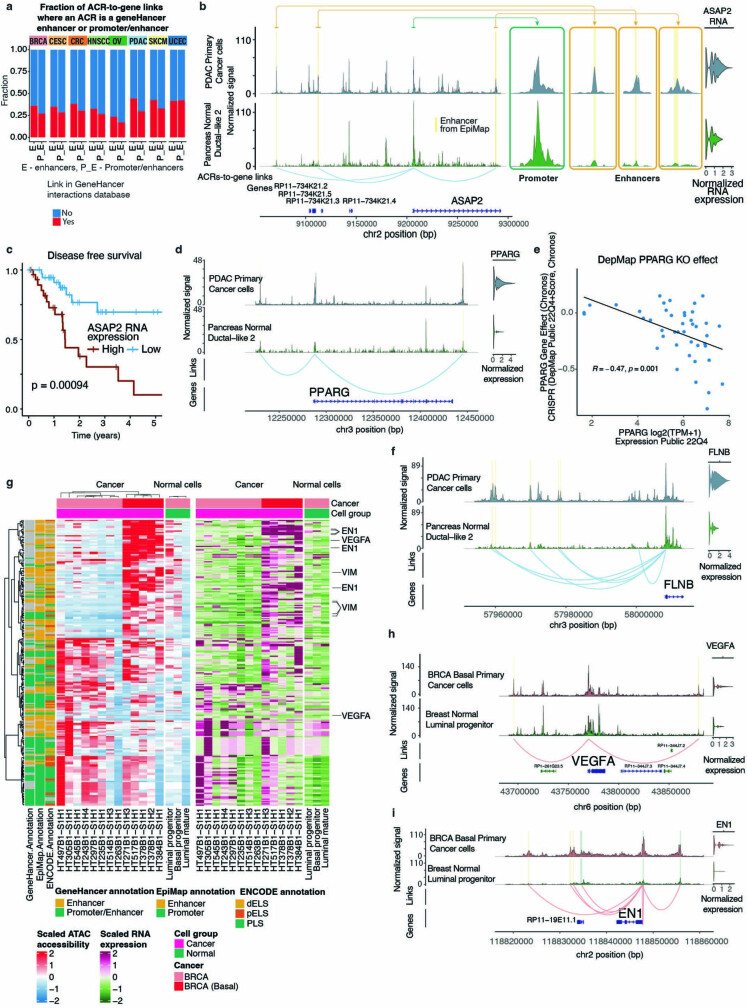
Cancer cell-specific enhancers. **a**, Bar chart showing proportions of ACR-to-gene links found in GeneHancer Interactions database (red and blue indicate found and not found, respectively). ACR-to-gene links were pre-filtered by ACRs overlapping with an element from GeneHancer regulatory elements database. **b**, Coverage plot of the *ASAP2* region in PDAC primary cancer cells and ductal-like-2 normal cells. Genomic regions highlighted in yellow correspond to EpiMap enhancers. Zoom-in views on enhancer and promoter regions on the right side provide finer detail. Violin plot of *ASAP2* RNA expression appears at far right (log2(FC) = 0.71, Wilcoxon rank-sum test two-sided *p-*value = 1.57^−87^). **c**, Kaplan-Meier plot of disease-free survival of TCGA PDAC patients stratified by *ASAP2* high and low expression (high *ASAP2*, n = 30; low *ASAP2*, n = 38, Log Rank Test *p-*value < 0.001). High *ASAP2* group was defined as the top 50% quantile of RNA expression and low *ASAP2* as the bottom 50%. *p-*value was calculated using the log-rank test. **d**, Coverage plot showing ATAC-seq accessibility of *PPARG* region and enhancers linked to *PPARG* expression. Both linked enhancers are DACRs between primary PDAC cancer cells and pancreatic ductal-like 2 cells. Violin plot on the right side indicates *PPARG* RNA expression in the same cells. **e**, Scatter plots showing CRISPR *PPARG* KO effect (Y-axis) in pancreatic cancer cell lines vs *PPARG* expression (X-axis). Data was obtained from DepMap portal. Pearson’s correlation coefficient and its *p-*value are shown. **f**, Coverage plot showing snATAC-seq accessibility of *FLNB* region and enhancers linked *FLNB* expression. All linked enhancers are DACRs between primary PDAC cancer cells and pancreatic ductal-like2 cells. Violin plot on the right side indicates *FLNB* RNA expression in the same cells. **g**, Heatmap of ACR-to-gene links connecting regions with increased accessibility in BRCA basal cancer cells with genes with increased expression in BRCA basal cancer cells. Heatmap shows average normalized and scaled snATAC-seq and snRNA-seq values aggregated by sample for cancer cells and by cell type for normal breast cells. snATAC-based heatmap is clustered using Ward’s minimum variance method (Ward.D2 from R) and Euclidean distance, snRNA-based heatmap columns and rows follow respective snATAC-based heatmap column and row orders. Label key: dELS - distal *cis-*regulatory regions (CREs) with enhancer-like signatures, pELS - proximal CREs with enhancer-like signatures, PLS - CREs with promoter-like signatures. **h**, Coverage plot showing snATAC-seq accessibility of *VEGFA* region and enhancers linked to *VEGFA* expression. All linked enhancers are DACRs between primary BRCA basal cancer cells and luminal progenitor cells. Violin plot on the right side indicates *VEGFA* RNA-seq expression in the same cells. **i**, Coverage plot showing snATAC-seq accessibility of *EN1* region and enhancers linked EN1 expression. All linked enhancers are DACRs between primary BRCA basal cancer cells and luminal progenitor cells. Violin plot on the right side indicates *EN1* RNA-seq expression in the same cells.

The large number of malignant nuclei sequenced using snMultiome-seq in 122 tumour samples from 8 cancer types and measurements of over half a million enhancer ACRs enabled us to predict enhancer ACRs regulating gene expression. First, we evaluated whether our dataset shows any global association patterns between accessibility of enhancer and promoter ACRs and gene expression. By correlating malignant and normal epithelial cells using accessibility and gene expression, we found that the accessibilities of enhancer elements were more specific to cancer types and tissue of origin than the accessibilities of promoters (Fig. 2a), consistent with other studies^6,26^. Enhancer accessibility also better reflected transcript expression than promoter accessibility did, suggesting their crucial role in regulating gene expression. To predict regulatory relationships between ACRs and gene expression, we computed correlation-based ACR-to-gene links (Methods). Nearly half of all significant ACR-to-gene links were between genes and EpiMap enhancer regions (Fig. 2b) and most of those enhancer ACR-to-gene links were cancer-type specific (Fig. 2c), supporting the tissue and cancer-type specificity of enhancers demonstrated in Fig. 2a. Only a modest fraction (25–35%) of the ACR-to-gene links was previously reported in the GeneHancer Interactions database (Extended Data Fig. 5a and Supplementary Table 3), indicating that the majority of the links that we identified in this study are previously undescribed.

We next sought to identify which linked ACRs and genes might be related to transition from normal to primary cancer cells. For each link, we required that both the ACR and the gene were a respective DACR and a differentially expressed gene (DEG; log_2_[fold change (FC)] > 0.25 and false-discovery-rate-adjusted *P* (FDR) < 0.05). We observed 397 linked ACRs (most of which are enhancers) gaining accessibility in most primary PDAC tumours (Fig. 2d). One proximal and two distal enhancers in particular were linked to the expression of recently reported oncogenic *ASAP2* in PDAC^27^ (Fig. 2d and Extended Data Fig. 5b), while the accessibility of its promoter did not change (Extended Data Fig. 5b). *ASAP2* encodes a GTPase-activating protein that activates the GTPases ARF1, ARF5 and ARF6^28,29^, influencing the dynamics of focal adhesions^28^. It also was shown to promote proliferation of PDAC and HCC cancer cells^27,30^. Expression of *ASAP2* was a similarly unfavourable prognostic factor in the TCGA PDAC cohort (Extended Data Fig. 5c).

Other notable examples of ACR-to-gene links include TF genes *KLF6* and *PPARG*, linked respectively to one and two enhancers that gain accessibility in PDAC cancer cells (Fig. 2d and Extended Data Fig. 5d). *PPARG* expression in pancreatic cancer is associated with worse survival^31^ and its knockout in pancreatic cancer cell lines leads to decreased cell proliferation^32^ (Extended Data Fig. 5e). Another unfavourable prognostic marker of PDAC, *FLNB*, was linked to five enhancer regions, suggesting extensive epigenetic regulation (Extended Data Fig. 5f). In the basal BRCA cohort, we observed several enhancers linked to the genes *EN1*, *VIM* and *VEGFA* (Extended Data Fig. 5g–i). The region between 10 kb upstream and 20 kb downstream of the *EN1* gained high accessibility compared with the CNC (Extended Data Fig. 5i), suggesting substantial epigenetic regulation of *EN1* expression. EN1 is a developmental TF that was shown to be a transcriptional dependency of triple-negative BRCA, promoting the survival of basal-like breast and other cancers^33,34^. As opposed to more proximal enhancers of *VEGFA* that gain accessibility in ccRCC, CRC and UCEC cancers (Extended Data Fig. 4g), distal upstream and downstream enhancers were upregulated in BRCA basal cancer (Extended Data Fig. 5h). These observations demonstrate the strength of ACR-to-gene analysis to identify potential regulatory relationships between distal elements and clinically relevant genes.

## Regulons in primary tumours

**Fig. 3:**
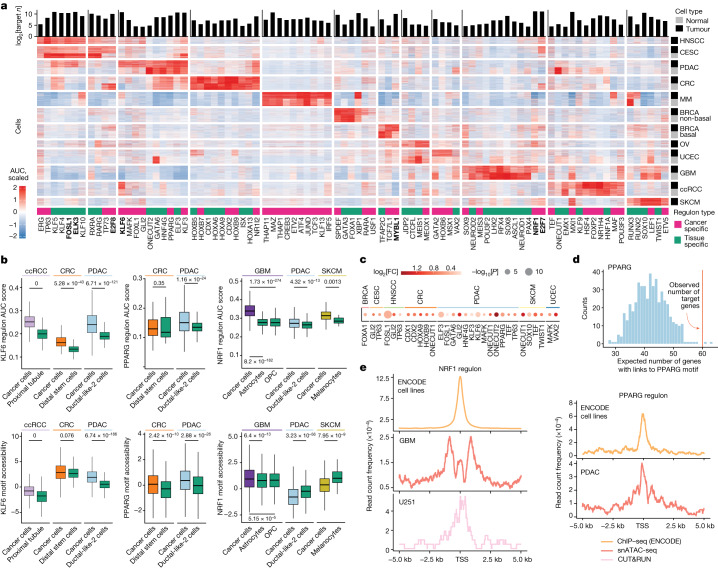
Pan-cancer and cancer-specific regulons. **a**, Tissue- and cancer-cell-specific regulons (columns) identified using SCENIC on sc/snRNA-seq data, where a regulon is a TF and its *n* target genes, with the number of genes shown at the top. The heat map shows scaled area under the curve (AUC) scores across 200 tumour and 200 normal randomly selected cells (rows) from each cancer. Cancer-specific regulons show higher activity in cancer cells versus the CNC. The top cancer-cell-specific regulons shared across several cancers are highlighted in bold. **b**, Regulon activity scores in primary cancer cells and corresponding CNCs (top; *n* = 2,211 (PDAC), *n* = 744 (ductal-like-2), *n* = 5,000 (ccRCC), *n* = 714 (proximal tubule), *n* = 446 (CRC), *n* = 184 (distal stem cells), *n* = 3,600 (GBM), *n* = 842 (OPC), *n* = 389 (astrocytes), *n* = 800 (SKCM) and *n* = 20 (melanocytes)) and TF motif accessibility scores (bottom; *n* = 30,428 (PDAC), *n* = 1,652 (ductal-like-2), *n* = 106,250 (ccRCC), *n* = 11,471 (proximal tubule), *n* = 6,243 (CRC), *n* = 860 (distal stem cells), *n* = 83,507 (GBM), *n* = 933 (OPC), *n* = 996 (astrocytes), *n* = 7,844 (SKCM) and *n* = 20 (melanocytes)). The boxes are coloured by cancer type, and the green boxes represent normal cells. FDR-adjusted Wilcoxon two-sided *P* values are shown (Supplementary Table 4c,e). For the box plots, the centre line shows the median, the box limits show the first and third quartiles, the upper and lower whiskers extend from the hinge to the largest or the lowest value no further than 1.5× the interquartile range (IQR) from the hinge. **c**, TFs of which the target genes are enriched for TF-specific ACR-to-gene links (ACR containing this TF-binding site). Colour indicates the log_2_[FC] between the observed number of target genes with such links over the expected number (*K*_1, ..., *n*_) of random genes with such links. One-sided *P* values were calculated for each regulon from a Gaussian *z* score, *z* = (*M* − *μ*)/*σ*, where *M* is the observed number of target genes linked to TF motifs. **d**, Example of the normal distribution of the number of genes with PPARG-specific PDAC ACR-to-gene links found in randomly sampled genes. The observed number of PPARG target genes with PPARG-specific ACR-to-gene links is indicated by the red line. **e**, The presence of ChIP–seq peaks (ENCODE), snATAC-seq peaks or CUT&RUN peaks around the TSS of target genes.

**Extended Data Fig. 6:**
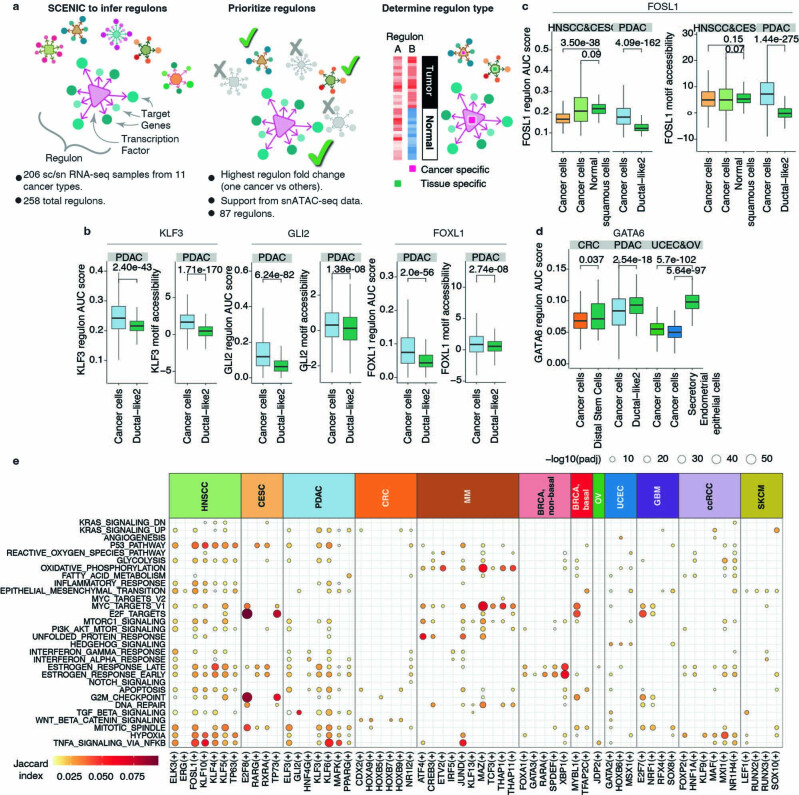
Tissue- and cancer cell-specific regulons. **a**, Schematic showing regulons identified using SCENIC across 11 cancer types (Methods). **b**, Box plots showing regulon activity scores and TF motif accessibility scores for KLF3, GLI2, and FOXL1 in primary PDAC cancer cells (*n* = 2,211 for regulon activity; *n* = 30,428 for TF motif) and normal pancreas ductal-like 2 cells (Ductal-like 2 *n* = 744 for regulon activity; *n* = 1,652 for TF motif). **c**, Box plots showing regulon activity scores and TF motif accessibility scores for FOSL1 in PDAC (*n* = 2,211) increase compared to those in squamous cancers (CESC *n* = 2,200; HNSCC *n* = 3,462) and normal squamous cells (*n* = 143). **d**, Box plots showing GATA6 regulon activity scores decreasing in CRC (*n* = 446), PDAC (*n* = 2,211), OV (*n* = 1,400), and UCEC (*n* = 800) cancer cells compared to respective CNCs (Distal stem cells *n* = 184; Ductal-like 2 *n* = 744; Secretory endometrial cells *n* = 202). In the **b-d** box plots for cancer cells are coloured by cancer types. Wilcoxon rank-sum test FDR adjusted two-sided *p-*values are shown (Supplementary Table S4c, e). Box plot center line corresponds to the median, the lower and upper hinges correspond to the first and third quartiles. The upper or lower whiskers extend from the hinge to the largest or the lowest value no further than 1.5*IQR from the hinge (where IQR is the inter-quartile range). **e**, Bubble plot showing pathway enrichment in target genes of tissue and cancer cell-specific regulons. Target genes were filtered out by tissue/cancer cell-specific DEGs in the respective cancer type (Supplementary Table 2b).

To better understand transcriptional regulations involved in cancer development, we sought to define TF target genes that underlie cell state. We used SCENIC^35^ to identify regulatory relationships between TFs and their target genes, namely the regulon, in each cancer cohort (Methods and Extended Data Fig. 6a). This analysis revealed 258 regulons with concordant gene expression between TFs and their targets (Supplementary Table 4a), each one containing between 20 and 4,310 target genes (median, 372). Of these, 87 regulons showed high specificity for certain cancer types (Fig. 3a and Supplementary Table 4b–e). Among those, we observed 41 regulons were tissue specific (shared between cancer cells and the CNCs) and 46 regulons were cancer cell specific (more active in cancer cells compared with in the CNCs; Supplementary Table 4c,e). Examples of tissue-specific regulons include FOXA1 and GATA3 in BRCA non-basal cancer, KLF4 and FOSL1 in CESC and HNSCC, HNF1A and KLF9 in ccRCC, and HNF4G and GATA6 in CRC and PDAC.

When compared to CNCs, several regulons showed enhanced activity in malignant cells (Fig. 3a and Supplementary Table 4c), including MYBL1 in BRCA basal, OV and UCEC; TP73 in CESC and HNSCC; KLF6 in PDAC and ccRCC (Fig. 3b); and NRF1 in PDAC, GBM and SKCM (Fig. 3b). The accessibilities of the KLF6 and NRF1 motifs were also increased in these cancers (Fig. 3b), further supporting enhanced activity of these TFs. Pancreatic cancer cells also showed enhanced activity of several PDAC-specific regulons, including PPARG, KLF3, FOXL1, MAFK and GLI2 (Fig. 3a,b and Extended Data Fig. 6b), and several regulons shared with squamous cells, such as TP63, FOSL1 and ELK3 (Fig. 3a and Extended Data Fig. 6c). Most of these TFs also had increased motif accessibility (Extended Data Fig. 6b,c and Supplementary Table 4d,e). Moreover, several regulons showed decreased activity in primary cancer cells, including GATA6, which is significantly reduced in UCEC, OV, PDAC and CRC (Extended Data Fig. 6d).

**Extended Data Fig. 7:**
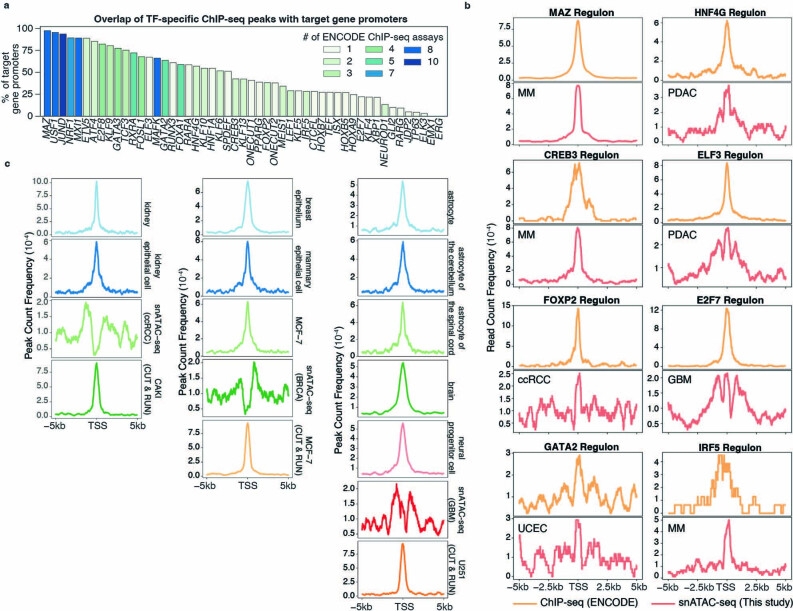
Regulon target genes validation. **a**, Bar plot summarizing the proportion of target gene promoters (within upstream and downstream 5 kb of TSS) that overlap with TF-specific ChIP-seq peaks for 53 TFs identified in Fig. 3a. Each bar represents a different TF, with the height of the bar indicating the proportion of target gene promoters that overlap with ChIP-seq peaks. The proportion is expressed as a percentage on the y-axis. **b**, TSS plots showing the presence of aggregated ChIP-seq peaks (ENCODE) and snATAC-seq peaks of target genes. **c**, TSS plots showing the presence of ChIP-seq peaks (ENCODE) from the corresponding biosamples, snATAC-seq peaks, and CUT&RUN peaks for CTCF’s target genes in CAKI (left), MCF-7 (middle), and U251 (right) cell lines.

We used various methods to further support the regulons that we prioritized (Supplementary Table 5 and Supplementary Note 4). We found that target genes of cancer-cell-specific regulons were enriched with cancer-specific pathways (Extended Data Fig. 6e), indicating their involvement in cancer-related processes. We also showed that target genes of 21 TFs were more likely to be linked to ACRs containing binding motifs of these TFs than random genes (Fig. 3c,d), validating the connections among target gene expression, ACR accessibility and TF activity. We next validated the target genes for each TF using TF-specific chromatin immunoprecipitation followed by sequencing (ChIP–seq) data from ENCODE^36^, corroborating direct binding to target genes in 51 out of 53 TFs that we examined (Extended Data Fig. 7a and Supplementary Table 5b,c). Our findings were further supported by a centred distribution of ChIP–seq peaks around the TSSs of target genes, indicating regulation of the target genes by the TFs (Fig. 3e and Extended Data Fig. 7b,c). These analyses not only confirm some of our findings, but also highlight the power of discovering new events with a larger pan-cancer cohort. To further validate our results, we performed a cleavage under targets and release using nuclease (CUT&RUN) assay in the U251 GBM cell line (Fig. 3e and Extended Data Fig. 7c), profiling the direct binding of NRF1 at the promoters of target genes. We observed a consistent pattern of binding across many different target genes, providing further evidence to support our findings.

## Epigenetic programs in cancer metastasis

Our cohort included 52 metastatic samples from 6 tumour types: SKCM (16), CRC (15), PDAC (13), UCEC (5), OV (2) and BRCA (1). Among those, we had 9 cases (5 UCEC and 4 CRC) with paired primary–metastatic samples, enabling us to directly evaluate the epigenetic changes that lead to metastasis in individual cases.

**Extended Data Fig. 8:**
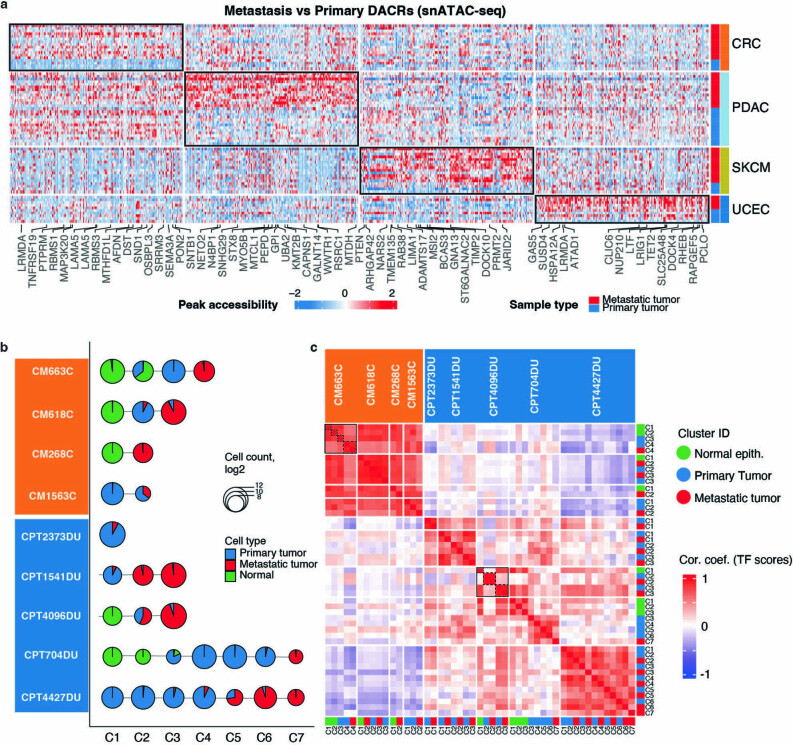
snATAC-seq based differences between primary and metastatic cancer cells. **a**, Heatmap showing top 200 up-regulated DACRs associated with the transition from primary to metastasis across four cancer types. Labels on the X-axis show the nearest genes to DACRs which also have significantly up-regulated gene expression in metastatic samples compared to primary tumour samples based on snRNA-seq data. For each sample (rows) peak accessibility was calculated as average across its cancer cells. **b**, Pie-charts showing contribution of primary cancer cells, metastatic cancer cells, and normal epithelial cells to each cluster of case-level objects (see Methods) from 5 UCEC and 4 CRC cases. **c**, Pearson correlation coefficient heatmap computed based on TF motif scores averaged per cluster and per sample, showing that cancer cells from the same cluster tend to be similar by their TF score profiles.

We first analysed transcriptional programs involved in metastasis using the four cohorts for which we had at least five metastasis samples, namely SKCM, CRC, PDAC and UCEC. We compared cancer cells from primary tumour samples and metastasis samples for each cohort, finding several important prognostic markers (Extended Data Fig. 8a and Supplementary Table 6a–c). *LAMA5* regulatory regions were upregulated across CRC liver metastasis samples, consistent with *LAMA5* promoting colorectal liver metastasis growth^37^. *GNA13* regulatory regions were upregulated in metastatic SKCM; *GNA13* is associated with proliferation and metastasis in several tumour types, but its specific role in melanoma is less understood^38,39^.

**Fig. 4:**
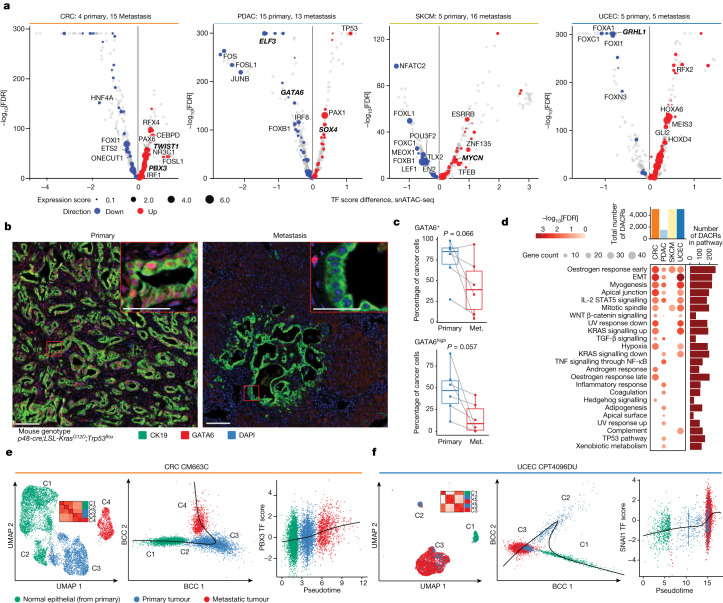
Epigenetic programs activated in cancer metastasis. **a**, TFs with differential motif accessibilities between metastasis and primary samples in four cancer types. The *y* axis shows FDR-adjusted *P* values calculated using two-sided Wilcoxon rank-sum tests. The expression score corresponds to the absolute value of the log_2_[FC] in TF expression between metastasis and primary cancer cells, using per sample average values, and requiring the same fold change direction as that of the motif score difference for the same TF–cancer pair. **b**, mpIHC analysis of GATA6 (red) expression in mouse models of PDAC. CK19 (green) marks cancer cells and DAPI (blue) marks nuclei. Scale bars, 100 μm (main images) and 50 μm (insets). **c**, The GATA6^+^ and GATA6^high^ cancer cell percentage was higher in primary PDAC compared with in matched metastatic (met.) PDAC. *n* = 6. *P* values calculated using two-sided paired *t*-tests are shown. For the box plots, the centre line shows the median, the box limits show the first and third quartiles, the upper and lower whiskers extend from the hinge to the largest or the lowest value no further than 1.5 × IQR from the hinge. **d**, Significant and suggestive (FDR ≤ 20%) hallmark pathway enrichments from DACRs upregulated in metastatic versus primary tumour. The bubble size and colour convey gene count and log_10_[FDR], respectively. The total number of DACRs per cancer type used in the analysis was capped at 5,000 by log_2_[FC] (top). The total number of DACRs annotated in each pathway is shown on the right. **e**,**f**, UMAP plots (left) for paired primary and metastasis samples of a CRC case (**e**) and a UCEC case (**f**). The small heat maps show Pearson’s correlation coefficients based on TF-motif scores averaged per cluster in each sample. Scatter plots showing cells ordered along the trajectories identified by Slingshot (centre), and scatter plots showing the association between PBX3 (**e**) or SNAI1 (**f**) motif accessibility and the progression of pseudotime (right) are shown.

To identify TFs that change their activity during the transition from primary to metastasis, we compared TF motif accessibility scores (Methods) between primary and metastatic cells across four cancers (SKCM, CRC, PDAC and UCEC). For CRC, we observed several TF motifs with consistently higher accessibilities in metastatic cells versus primary cancer cells, including the epithelial to mesenchymal transition (EMT) master regulator TWIST1^40^, and PBX3, which promotes migration of CRC cells^41^ (Fig. 4a and Supplementary Table 6d–f). In PDAC, we found ELF3 and GATA6 among the top significant TFs with decreased motif accessibilities. ELF3 is associated with the epithelial phenotype and represses EMT^42^, and it was also identified as a tissue-specific regulon in our data (Fig. 3a). GATA6 regulates EMT and inhibits EMT in vitro and cell dissemination in vivo in pancreatic cancer^43^. GRHL1 was one of the top TFs with decreased motif accessibility in metastasis compared with in the primary tumour in UCEC and induces epithelial cell adhesion molecules and represses genes that are involved in cell migration and invasion^44^. We also observed multiple members of FOX-family TFs among the most significant downregulated TFs across cancers. FOX-family motifs were also enriched in DACRs that were upregulated in primary PDAC and UCEC (Supplementary Note 4). FOXA1 represses genes associated with EMT^45^ and FOXN3 represses growth and invasion in some cancers^46,47^. These results support the idea that both common and specific TFs are involved in the process of metastasis across different cancers. Next, we used the genetically engineered mouse models of PDAC driven by *Kras*^*G12D*^ mutation and *Trp53* loss (*p48-cre;LSL-Kras*^*G12D*^*;Trp53*^*flox*^) to validate decreasing activity of GATA6 in PDAC metastases. Specifically, we performed multiplex immunohistochemistry (mpIHC) analysis of GATA6 and cytokeratin 19 (a cancer cell marker) in matched primary tumours and metastases in liver tissues. Consistent with observations from human snATAC-seq data analysis, we found fewer GATA6^+^ and GATA6^high^ PDAC cancer cells in all liver metastases compared with in their matched primary pancreatic tumours (Fig. 4b,c; paired *t*-test, *P* = 0.066 and *P* = 0.057, respectively).

We further evaluated the pathways that are enriched in DACRs upregulated between metastatic and primary tumour samples in each of these four cohorts, and found that development-related pathways—such as EMT, myogenesis and apical junction—were significant in three cohorts. This observation is consistent with the fact that the loss of the epithelial phenotype is an important process involved in metastasis (Fig. 4d). We also observed pathways that were enriched specifically in individual cohorts, for example, TNF signalling was significant in PDAC, consistent with the known KRAS-induced NF-κB activation in PDAC^48^.

**Extended Data Fig. 9:**
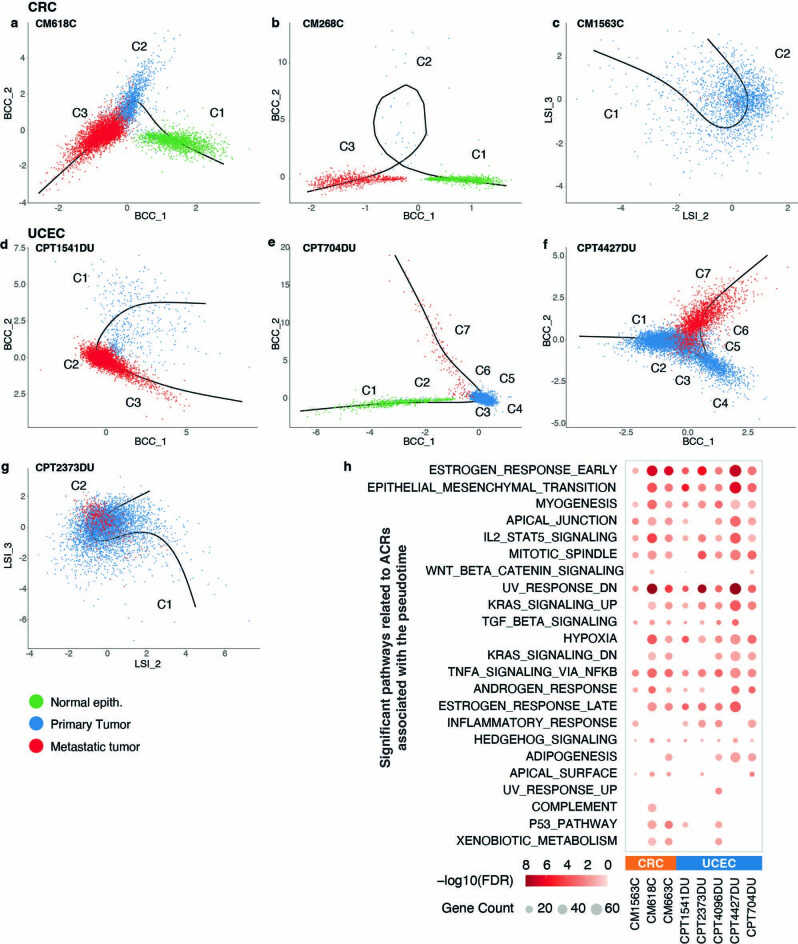
Pseudotime trajectories of UCEC and CRC cases, and significant pathways enriched in ACRs associated with pseudotime. **a-g**, Plots of trajectories found by Slingshot for 3 CRC (**a-c**) and 4 UCEC (**d-g**) cases coloured by cell type. Each case (except for CM1563C and CPT2373DU) is plotted using Between Cluster Analysis (BCA, a novel supervised dimensionality reduction method) and visualized on the first two between cluster components (BCCs). Cases CM1563C and CPT2373DU, which have only two cell types as well as two or less case clusters, were visualized with the second and third LSI components instead. Each trajectory starts from the normal cell type if available; otherwise, it starts from the primary tumour cell type. **h**, Bubble plot of significant pathways enriched in ACRs significantly associated with pseudotime (from normal to primary cancer cells, and then to metastatic cancer cells). The significant ACRs were found by regressing them against pseudotime using lasso regression.

Finally, we analysed snATAC-seq data for the nine CRC and UCEC cases with both primary and metastasis samples available. First, by combining normal epithelial cells with cancer cells, we observed distinct clusters composed of neoplastic cells, with prevalence varying between primary and metastatic samples in each cluster (Fig. 4e,f and Extended Data Fig. 8b). We next evaluated TF-motif accessibility profiles of primary, metastatic and normal epithelial cells in each cluster from those nine cases (Extended Data Fig. 8c). By conducting trajectory analysis of tumour and normal epithelial cells, we observed that all of the paired primary–metastatic samples followed a linear trajectory, gradually progressing from normal (if available) to primary to metastatic cells (Fig. 4e,f and Extended Data Fig. 9a–g). This suggests that trajectory pseudotimes reflect the metastatic process. We found that the trajectories of samples were positively correlated with known EMT-specific motifs and other motifs implicated in the metastases, such as SNAI1 and PBX3^41^ (Fig. 4e,f, Supplementary Note 5 and Supplementary Table 7). Leveraging the trajectory analysis, we identified ACRs that are significantly associated with pseudotimes (Methods) and further evaluated pathways that are enriched in those ACRs (Extended Data Fig. 9h) from nine cases. Although some variations were observed across samples, the top pathways (for example, EMT, myogenesis, apical junction, early oestrogen response) enriched in metastases found at the cohort level were redetected in the majority of the cases (Extended Data Fig. 9h and Supplementary Note 6).

## Genetic and epigenetic interactions

**Extended Data Fig. 10:**
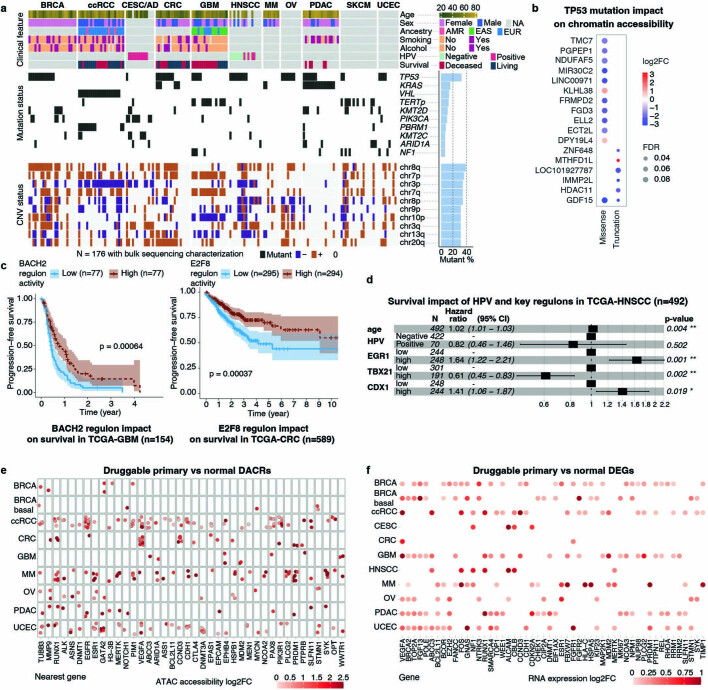
Summary of somatic drivers, druggable targets, and prognostic significance. **a**, Summary heatmap showing the landscape of genetic drivers (i.e., somatic mutations, CNVs) and clinical annotation for the samples with bulk WES available in this cohort (*n* = 176). **b**, Dotplot showing DACRs between *TP53* missense mutant samples and *TP53* WT, or between *TP53* truncation mutants and *TP53* WT in BRCA, using sample-level snATAC-seq ACRs accessibilities. For this analysis we used only ACRs that were supported by TP53 ChIP-seq obtained from ENCODE^36^ and that also contained a TP53 binding motif. Dot size corresponds to two-sided Wilcoxon rank-sum test FDR adjusted *p-*values. **c**, The Kaplan-Meier plots and analysis of progression-free survival in TCGA-GBM cohort stratified by BACH2 regulon activity (left) and in TCGA-CRC stratified by E2F8 regulon activity (right). Error bands represent 95% confidence intervals; two-sided *p*-values by the log-rank (Mantel–Cox) test are indicated; *n* is specified for each patient subgroup; high and low regulon activity groups are defined based on values above and below the median, respectively. **d**, A forest plot showing the hazard ratio (X-axis, the center of error bars) and 95% confidence intervals (error bars) associated with regulons and HPV status (Y-axis) identified by multiple Cox proportional-hazards models with overall survival adjusted for age and sex in TCGA-HNSCC cohort. Cox proportional hazards model two-sided *p-*values are shown; high and low regulon activity groups are defined based on values above and below the median, respectively. **e**, Bubble plot showing druggable targets as annotated in CIViC database^102^ found by DACR analysis between primary cancer cells and corresponding CNCs. Each dot is one DACR. X-axis shows the nearest gene. **f**, Bubble plot showing druggable targets as annotated in CIViC database^102^ found by DEG analysis between primary cancer cells and corresponding CNCs. Each dot is one DEG.

To facilitate the investigation of how epigenetic drivers interact with genetic drivers, we performed genetic characterizations of somatic mutations and copy-number variations (CNVs) on the 176 tumour samples with available WES data (Supplementary Table 8). The mutation burdens across cancer types were similar to those that were previously reported^49^ (Extended Data Fig. 10a and Supplementary Note 7). We further investigated the impact of *TP53* missense and truncation mutations on chromatin accessibility within BRCA samples, which were enriched for *TP53* mutations (Extended Data Fig. 10b). For this analysis, we used ACRs that overlapped with TP53 ChIP–seq peaks obtained from ENCODE^36^, also requiring that they contain a TP53*-*binding motif. The only ACR identified in both comparisons was the one associated with *GDF15*—a known target of TP53^50^ that mediates G1 cell cycle arrest and apoptosis and is involved in treatment resistance and maintenance of BRCA stem-like cells^51^. *FGD3*, a positive prognostic feature in BRCA that inhibits cell migration^52^, was identified in DACRs associated with wild-type versus missense *TP53*.

**Fig. 5:**
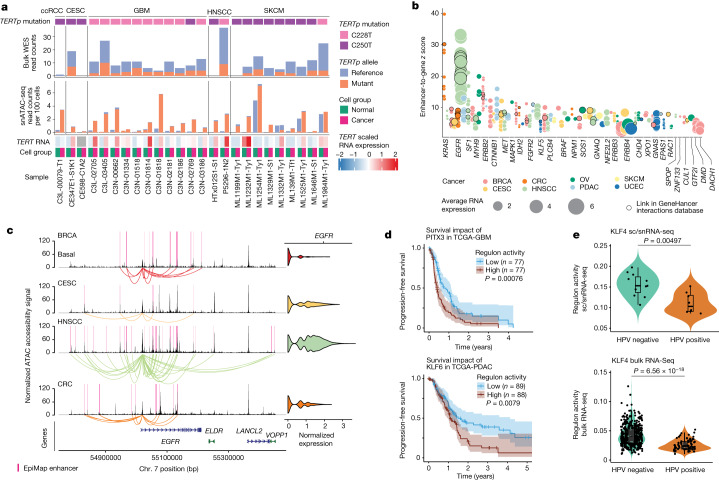
The impact of genetic drivers on chromatin accessibility. **a**, *TERTp* mutations (C228T and C250T) detected in five cancer types from snATAC-seq data and WES data. Read counts supporting the reference or mutant allele in bulk WES data (top) and snATAC-seq data (bottom) are shown. snATAC-seq-supported reads were counted separately for cancer cells and normal cells and then normalized to the total number of cells in each group. The heat map at the bottom shows *TERT* expression in cancer and normal cells per sample. **b**, Epigenetic regulation of known oncogenes identified using snMultiome-seq data. Each dot shows one enhancer-to-gene link *z* score. The enhancer-to-gene *z* score was computed by averaging ACR-to-gene link *z* scores for all ACRs falling into one enhancer, as annotated in the EpiMap or GeneHancer database. The dot colour corresponds to a cancer type in which an ACR-to-gene link was identified, and the dot size corresponds to normalized RNA expression of the genes shown on the *x* axis. **c**, Coverage plot of the *EGFR* region in BRCA basal, CESC, HNSCC and CRC cancer cells. Only samples with neutral *EGFR* CNV were included. *EGFR* RNA expression is shown on the right. **d**, Kaplan–Meier plots and analysis of progression-free survival in the TCGA-GBM cohort stratified by PITX3 regulon activity (top) and in the TCGA-PDAC cohort stratified by KLF6 regulon activity (bottom). The error bands represent the 95% confidence intervals. Two-sided *P* values calculated using log-rank (Mantel–Cox) tests are indicated. High- and low-regulon-activity groups are defined on the basis of values above and below the median, respectively. *n* is specified for each patient subgroup. **e**, Regulon activity of KLF4 in HPV-positive and HPV-negative HNSCC samples from this study (top) and TCGA-HNSCC (bottom). *P* values calculated using two-sided Wilcoxon rank-sum tests are shown.

We also investigated the accessibility of *TERT* promoter (*TERTp*) with hotspot mutations in cancer and normal cells. We profiled two *TERTp* hotspot mutations C228T (chromosome 5, 1295113, G to A) and C250T (chromosome 5, 1295135, G to A) in the analyses. The C228T mutation was primarily detected in GBM cancer cells, whereas the C250T mutation was typically observed in SKCM cancer cells. Out of all samples, 25 showed *TERTp* mutations, with the majority of the variants preferentially accessible in cancer cells, which was also in accordance with high *TERT* expression from snRNA-seq data (Fig. 5a). Conversely, in normal cells, the snATAC-seq coverage for *TERTp* positions was notably lower, indicating the absence of *TERTp* accessibility in normal cells (Fig. 5a). Compared with snATAC-seq data, bulk WES had a much lower variant allele frequency of *TERTp* mutations, indicating that snATAC-seq enables the detection of mutations that induce chromatin accessibility (Fig. 5a).

We have also evaluated the epigenetic regulation of oncogenes^49^ by correlating their expression with the accessibilities of the enhancers (ACR-to-gene links). We identified 30 oncogenes of which the expression was linked to enhancer accessibility, with the strongest links in *EGFR*, *KRAS*, *ERBB2*, *CTNNB1* and *MET* (Fig. 5b). Many oncogenes showed numerous enhancer-to-gene links suggesting extensive and complex epigenetic regulation of these genes. *EGFR* showed the highest number of links in HNSCC; this observation aligned with the highest *EGFR* expression and highest accessibility of enhancers in the *EGFR* gene body (Fig. 5c (only *EGFR* WES-based CNV-neutral samples were included in the analysis) and Supplementary Table 8j). Accessibility of the *EGFR* gene body in CESC was similar to that in HNSCC. However, accessibility of upstream enhancers was less prominent, which could explain lower *EGFR* expression in CESC. Finally, *EGFR* accessibilities in BRCA basal and CRC were even less pronounced, showcasing granular epigenetic regulation of *EGFR* independent of its CNV.

## Clinically relevant epigenetic programs

We next searched for epigenomic programs with potential clinical relevance. PITX3 regulon activity was notably higher in GBM cancer cells (FDR = 0.002) than in OPCs (Supplementary Table 4c). Regulon activity scored using bulk RNA-seq expression data in TCGA patients with GBM showed that increased PITX3 activity was associated with poor progression-free survival (hazard ratio (HR) = 1.86, log-rank *P* = 0.00076) and poor overall survival (HR = 1.84, log-rank *P* = 0.00085) (Fig. 5d and Supplementary Table 9a,b). In the TCGA-PDAC cohort, increased KLF6 regulon activity was also associated with poor progression-free and overall survival (Fig. 5d and Supplementary Table 9a,c). This observation is corroborated by our earlier findings of increased KLF6 regulon activity and its motif accessibility (Fig. 3b), and also increased gene expression and enhancer accessibility linked to *KLF6* (Fig. 2d) in primary PDAC cancer cells compared with in ductal-like-2 cells. By contrast, the tissue-specific regulons E2F8 (CRC) and BACH2 (GBM) had higher scores in the respective CNCs (Supplementary Table 4c) and were linked to better survival in the TCGA-CRC and TCGA-GBM cohorts, respectively (Extended Data Fig. 10c and Supplementary Table 9b,d).

We further investigated the impact of human papillomavirus (HPV) status on the landscape of active TFs (Methods and Supplementary Table 1b). We observed a marked decrease in KLF4 regulon activity in HPV-positive tumours compared with in HPV-negative tumours in this study, which was further validated in the TCGA-HNSCC cohort (Fig. 5e and Supplementary Table 9e). We identified additional regulons with potentially altered activity in HPV-positive HNSCC samples compared with their HPV-negative counterparts (Supplementary Table 9e). Furthermore, we identified CDX1, EGR1 and TBX21 as additional factors affecting the overall survival in patients of the TCGA-HNSCC cohort (Methods and Extended Data Fig. 10d). Specifically, patients with increased TBX21 regulon activities tend to have a better survival, whereas increased CDX1 and EGR1 portends poorer prognosis.

Finally, to evaluate for therapeutically relevant genetic and epigenetic alterations, we identified cancer-specific DEGs and DACRs that are potential drug targets (Methods). Among druggable DEGs and DACRs, we observed some well-known examples, including *ESR1* expression and accessibility in BRCA and UCEC and *VEGFA* accessibility in ccRCC and CRC (Extended Data Fig. 10e,f). Moreover, we observed instances of known drug targets enriched in cancer types for which these targets are not used in clinical practice. Among these were *EGFR* accessibility in ccRCC, *TOP1* expression in UCEC, MM and ccRCC, and *FGFR2* expression in GBM, ccRCC and basal BRCA. These associations may indicate targets that could be used therapeutically in these tumour types and warrant further preclinical validation.

## Discussion

We created and investigated a large-scale single-cell multi-omic atlas of tumours and NATs from 225 samples across 11 cancer types, unveiling diverse cancer and normal tissue cell types. Advancing beyond previous bulk ATAC/RNA-seq studies, our analysis provides nuanced insights into cancer biology, including cancer-specific epigenetic architecture, relationships between normal and malignant cells, and primary-to-metastatic transitions in the same lineage. We identified CNC types on the basis of shared chromatin accessibility patterns with cancer cells, which may be suggestive of cell lineage and underscores the importance of epigenetic architecture in determining cell-of-origin, as well as offering important insights into the transition from normal cells to cancer. Identification of changes in chromatin accessibility between primary and metastatic cancers of the same type and comparison across tumour types highlighted both commonalities and distinctions in chromatin landscape and epigenetic programs governing cancer progression across cancer types.

Chromatin accessibility differences between primary and metastatic tumours may hint at ways to interrupt metastatic transition. GATA6 TF motifs are depleted in open chromatin and *GATA6* expression is decreased in metastatic PDAC compared with primary PDAC. Furthermore, GATA6 regulon activity decreases in PDAC, CRC, OV and UCEC primary cancer cells compared with their respective CNCs. GATA6 loss in PDAC induces an EMT phenotype and is associated with basal subtype and decreased overall survival, consistent with this finding^43^. We identified many putative epigenetic drivers that have not been previously reported and *cis-*regulatory elements that correlate with important TFs. We also identified proximal and distal enhancers that are linked to *ASAP2* upregulation in primary PDAC cells, which promotes cell migration and tumour growth in vitro and in PDAC xenograft models^27^, as well as identifying genetic drivers (*TP53* mutant in BRCA) that influence the chromatin accessibility of genes that are involved in cell motility, invasion and proliferation (*GDF15* and *FGD3*). Integrated analysis of bulk WES, snRNA-seq and snATAC-seq in this study further highlights the allele-specific chromatin accessibility effect of *TERTp* mutations across cancers. The finding of strong associations between cancer drivers, such as *EGFR*, *KRAS* and *MET* and their associated enhancers, further stresses the importance of epigenetic and genetic interaction during tumorigenesis. Further functional validation will shed light on key regulatory pathways in cancer (Supplementary Note 8).

Understanding the landscape of chromatin architecture across tumours, chromatin accessibility changes at critical cancer transitions, and the interplay between chromatin accessibility, genetic alterations and transcriptional patterns is crucial to advancing cancer biology and clinical practice. Certain changes in chromatin accessibility that represent critical events/drivers of cancer initiation and metastatic spread may be potential therapeutic targets. Although TFs themselves are very difficult to target with traditional therapeutics and their many-varied roles in normal tissues raise concerns for off-target effects, we highlighted potentially targetable elements by focusing on broad transcriptional programs. Finally, we anticipate that this atlas will be a valuable resource for future cancer studies.

## Methods

### Specimen data

All samples for MM, OV, BRCA, PDAC, UCEC, CRC, CESC/AD, SKCM and HNSCC, as well as 2 NATs for GBM and 1 NAT for ccRCC were collected with informed consent in concordance with Institutional Review Board (IRB) approval at the School of Medicine at Washington University in St Louis. IRB protocols were as follows: 201105374, 201108117, 201407156, 202106166, 201911095, 201102270, 201103136 and 201102312. Tumour samples were collected during surgical resection and verified by standard pathology. GBM and ccRCC samples originating from the NCI CPTAC were part of previous studies^12,53^.

### Experimental methods

#### Nucleus lysis for snMultiome-seq

Approximately 20–30 mg of flash-frozen or cryopulverized or 200 μm of OCT sections of tissue from each sample was retrieved and aliquoted for nucleus preparation for the Chromium Next GEM Single Cell Multiome ATAC + Gene Expression sequencing protocol for simultaneously profiling epigenomic landscape and gene expression in the same individual nuclei. The samples were resuspended in lysis buffer (10 mM Tris-HCl (pH 7.4) (Thermo Fisher Scientific, 15567027), 10 mM NaCl (Thermo Fisher Scientific, AM9759), 3 mM MgCl_2_ (Thermo Fisher Scientific, AM9530G), NP-40 substitute (Sigma-Aldrich, 74385-1L), 1 M DTT (Sigma-Aldrich, 646563), 10% stock BSA solution (MACS, 130-091-376), nuclease-free water (Invitrogen, AM9937), plus 0.1 U  μl^−1^ RNase Inhibitor), resuspended and homogenized using a pestle, and filtered through a 40 μm cell strainer (pluriSelect), then washed with wash buffer (2% BSA + 1× PBS + RNase inhibitor). The filtrate was collected, then centrifuged at 500*g* for 6 min at 4 °C. The nuclear pellet was then resuspended in BSA wash buffer with RNase inhibitor, stained with 7AAD, and nuclei were purified and single-cell sorted using fluorescence-activated cell sorting (FACS). After counting and microscopy inspection of nucleus quality and concentration, nucleus suspensions were incubated in a transposition mix that included a transposase, while adapter sequences were simultaneously added to the ends of the DNA fragments and the preparation was diluted to 3,000–8,000 nuclei per μl to be used as one reaction for downstream preparation of both ATAC and gene expression preparation. About 20,000 nuclei were used for analysis using the Next GEM Single Cell Multiome ATAC + Gene Expression kit (10x Genomics) and gel beads in emulsion (GEMs) using the Chromium Next GEM Chip J Single Cell Kit, 16 rxns (PN-1000230). After post GEM-RT cleanup, the pre-amplification step was performed and the pre-amplified product was used as the input for both ATAC library construction and cDNA amplification for gene expression library construction. cDNA amplification/tagging was performed with 16 nucleotide barcodes and 10 nucleotide molecular identifiers during the reverse transcription (RT) reaction. After pre-amplification, the sample was divided and used as an input for two separate steps: 40 μl of the sample was used for ATAC library construction and 35 μl of the sample was used for cDNA amplification. Only 25% of the total cDNA was used for generation of GEX libraries for snRNA-Seq. Libraries were sequenced using the 10x Genomics Single Index N Set according to the manufacturer’s protocol for snATAC; and, for snRNA the 10x Genomics Dual Index TT Set A was used according to the manufacturer’s protocol for library preparation.

#### FACS analysis

Depending on the pellet size, 100–500 μl of nucleus suspension in the wash buffer (2% BSA + 1× PBS + RNase inhibitor) was stained with DRAQ5 or 7AAD for RNA or ATAC sequencing, respectively. Specifically, snRNA-seq nuclei were stained with 1 μl of DRAQ5 per 300 μl of the sample and snATAC-seq nuclei were stained with 1 μl of 7AAD per 500 μl of the sample. Sorting gates were based on size, granularity and dye staining signal.

#### Multiple myeloma single-cell suspension preparation and sequencing

Bone marrow mononuclear cell aliquots were centrifuged, after thawing, at 300*g* for 5 min to pellet cells. All supernatants were removed. To prepare cells for processing using the Miltenyi Dead Cell Removal Kit, cells were resuspended in 100 μl of beads and incubated at room temperature for 15 min. Cells were then run through the DepleteS selection using the autoMACS Pro Separator. The negative fraction (live cells) was pelleted by centrifugation at 450*g* for 5 min. Cells were finally resuspended in ice-cold phosphate-buffered saline (PBS) and 0.5% BSA and loaded onto a 10x Genomics Chromium Controller. The samples were loaded using the 10x Genomics Chromium Next GEM Single Cell 3′ GEM, Library & Gel Bead Kit v2. Barcoded libraries were then pooled and sequenced on the Illumina NovaSeq 6000 system with associated flow cells.

#### Nucleus lysis for snRNA-seq and snATAC-seq

First, 15–25 mg of pulverized tissue was placed into a 5 ml Eppendorf tube on ice. Using a wide-bore pipette tip (Rainin), a lysis buffer prepared according to the nucleus-isolation protocol (10x Genomics) and SuperRNase inhibitor (Invitrogen) were added to the tube. The tissue solution was gently pipetted until the lysis liquid turned a slightly cloudy colour (the number of pipetting iterations depended on the specific tissue). The tissue homogenate was then filtered through a 40 μm strainer (pluriSelect) and washed with a BSA wash buffer (2% BSA + 1× PBS + RNase inhibitor). The filtrate was collected, centrifuged at 500*g* for 6 min at 4 °C and resuspended with a BSA wash buffer. Then, 100 μl of cell lysis solution was set aside as the unstained reference, and the rest was stained with 7AAD or DRAQ5 depending on the ATAC or RNA protocol. Nuclei underwent FACS and sorting gates were based on size, granularity and dye staining signal. The final suspension was centrifuged at 500*g* for 6 min at 4 °C and resuspended with a BSA wash buffer. More specific details about the RNA protocol can be found at protocols.io (10.17504/protocols.io.14egn7w6zv5d/v1 (RNA protocol); for the ATAC protocol, 7AAD was substituted for dye).

#### 10x library preparation and sequencing of snRNA-seq and snATAC-seq

Nuclei and barcoded beads were isolated in oil droplets using the 10x Genomics Chromium instrument. Single-nucleus suspensions were counted and adjusted to a range of 500 to 1,800 nuclei per µl using a haemocytometer. Reverse transcription was subsequently performed to incorporate cell- and transcript-specific barcodes. All snRNA-seq samples were run using the Chromium Next GEM Single Cell 3’ Library and Gel Bead Kit v3.1 (10x Genomics). For snATAC-seq, the Chromium Next GEM Single Cell ATAC Library and Gel Bead Kit v1.1 prep (10x Genomics) was used for a subset of samples. For the multiome kit, the Chromium Next GEM Single Cell Multiome ATAC + Gene Expression kit was used. Barcoded libraries were then pooled and sequenced on the Illumina NovaSeq 6000 system with the associated flow cells.

#### Genomic DNA extraction

Tumour tissues and corresponding normal adjacent tissue were obtained from surgically resected specimens and, after a piece was removed for fresh single-cell preparation, the remaining sample was snap-frozen in liquid nitrogen and stored at −80 °C. Before bulk DNA extraction, the samples were cryo-pulverized (Covaris) and aliquoted for bulk extraction methods. Genomic DNA was extracted from tissue samples using either the DNeasy Blood and Tissue Kit (Qiagen, 69504) or the QIAamp DNA Mini Kit (Qiagen, 51304). Genomic germline DNA was purified from cryopreserved peripheral blood mononuclear cells using the QiaAMP DNA Mini Kit (Qiagen, 51304) according to the manufacturer’s instructions (Qiagen). The DNA quantity was assessed by fluorometry using the Qubit dsDNA HS Assay (Q32854) according to manufacturer’s instructions (Thermo Fisher Scientific).

#### WES data generation

A total of 100–250 ng of genomic DNA was fragmented on the Covaris LE220 instrument targeting 250 bp inserts. Automated dual-indexed libraries were constructed using the KAPA Hyper library prep kit (Roche) on the SciClone NGS platform (Perkin Elmer). Up to ten libraries were pooled at an equimolar ratio by mass before the hybrid capture targeting a 5 µg library pool. The library pools were hybridized using the xGen Exome Research Panel v1.0 reagent (IDT Technologies) that spans a 39 Mb target region (19,396 genes) of the human genome. The libraries were hybridized for 16–18 h at 65 °C followed by stringent wash to remove spuriously hybridized library fragments. Enriched library fragments were eluted and PCR cycle optimization was performed to prevent over amplification. The enriched libraries were amplified using the KAPA HiFi master mix (Roche) before sequencing. The concentration of each captured library pool was determined by quantitative PCR (qPCR) using the KAPA library Quantification Kit according to the manufacturer’s protocol (Roche) to produce cluster counts appropriate for the Illumina NovaSeq 6000 instrument. 2 × 150 bp paired-end reads were generated targeting 12 Gb of sequence to achieve around 100× coverage per library. Matching WES data were generated for 195 out of the 225 snATAC-seq samples. Of these 195 samples, tumour was available and used to generate 173 WES libraries corresponding to 176 of the snATAC-seq samples.

#### Cell lines

The Caki-1 cell line was purchased from ATCC (ATCC, HTB-46, https://www.atcc.org/products/htb-46) and authenticated using short-tandem-repeat (STR) profiling by ATCC. The MCF7 cell line was purchased from ATCC (ATCC, HTB-22, https://www.atcc.org/products/htb-22) and authenticated by STR profiling by ATCC. The U251 cell line was obtained from a previous study^54^, and was authenticated by STR profiling. No cell line used in this paper is listed in the database of commonly misidentified cell lines maintained by the International Cell Line Authentication Committee (ICLAC). All of the cell lines used here tested negative for mycoplasma contamination using MycoAlert (Lonza, LT07-118).

#### CUT&RUN experiment

The Caki-1, MCF7 and U251 cell lines were cultured under designated conditions according to information on the American Type Culture Collection (ATCC) website (https://www.atcc.org/). When cells reached the desired confluence and numbers, the CUT&RUN Kit (14-1048, EpiCypher) and CUT&RUN Library Prep Kit (14-1002, EpiCyher) were applied according to the manufacturer’s protocols. In brief, wash buffer, cell permeabilization buffer and antibody buffer were freshly prepared on day 1. ConA Beads were activated by washing and then diluted with a cold bead activation buffer. After these steps, 500,000 cells were collected for each reaction, followed by resuspending in a wash buffer and mixing well with activated beads. After 10 min of incubation at room temperature, the tubes were placed on an 8-strip magnet until the slurries cleared. The supernatant was removed and a cold antibody buffer was added to each reaction. The SNAP-CUTANA K-MetStat Panel was first added to the reactions designed for positive (H3K4me3) and negative (IgG) control antibodies. Then, 0.5 μg designated antibody was added to each reaction and incubated overnight. The next day, antibody-bound histone PTM or chromatin-interacting protein was washed with the cell permeabilization buffer. Next, pAG-MNase was added to cleave target-DNA complexes. Targeted chromatin was then digested and released by adding calcium chloride, *Escherichia coli* spike-in DNA and Stop Buffer Master Mix. DNA was purified, and up to 5 ng CUT&RUN-enriched DNA was used for further library construction according to the CUTANA CUT&RUN Library Prep Kit. Library fragment sizes were analysed using the TapeStation and the libraries were sequenced.

The following antibodies were used in CUT&RUN analysis: anti-NRF1 (46743, Cell Signalling Technology) and anti-CTCF (3418, Cell Signalling Technology).

#### Validation using mouse models

The following mouse strains (*Mus musculus*) were used as part of this study: p48-Cre mice (C57BL/6J background; laboratory of S. Hingorani); LSL-KrasG12D mice (C57BL/6J background; Jackson Laboratory, 008179); *Trp53*^*flox*^ mice (C57BL/6J background; Jackson Laboratory, 008462). All animal studies were completed in accordance with NIH-AALAC standards and consistent with Washington University School of Medicine IACUC regulations (protocol, 22-0233), and studies were approved by Washington University School of Medicine Institutional Animal Studies Committee. All animals were housed in a barrier facility under a 12 h–12 h light–dark cycle with 1–5 mice per cage.

For mpIHC analysis of mouse PDACs, embedded tissues were sectioned into 6 μm sections and loaded into BOND RXm (Leica Biosystems) for a series of staining, including using antibodies against GATA6 (Invitrogen, PA1-104) and CK19 (Cell Signaling Technology, 12434). On the basis of antibody host species, the default manufacturer protocols were used (IntenseR and Polymer Refine), including antigen retrieval with citrate buffer, goat serum and peroxide block; primary antibody incubation; post-primary incubation; and chromogenic visualization using an AEC substrate (Abcam). Between every two cycles of staining, the slides were manually stained with haematoxylin and eosin, then scanned using the Axio Scan.Z1 (Zeiss) system. The slides were then destained by a gradient of ethanol plus a 2% hydrochloride wash and blocked with extra avidin/biotin (Vector Laboratories) and a Fab fragment block (Jackson Laboratory). Citrate-based antigen retrieval was performed before each staining cycle. Images of the same specimen, but using different stains, were cropped into multiple segments using Zen (Zeiss). Each segment was then deconvoluted (Deconvolution, v.1.0.4; Indica Labs) for individual stains and fused using HALO software (Zeiss) with the default manufacturer’s settings. Markers of interest were pseudocoloured and quantified using the High Plex FL module within the HALO software.

### Analytical methods

#### WES reads alignment

FASTQ files were preprocessed using trimGalore v.0.6.7 (with the parameter --length 36 and all of the other parameters set to default; https://github.com/FelixKrueger/TrimGalore). FASTQ files were then aligned to the GDC’s GRCh38 human reference genome (GRCh38.d1.vd1) using BWA-mem^55^ v.0.7.17 with parameter -M and all of the other parameters set to default. The output SAM file was converted to a BAM using samtools (https://github.com/samtools/samtools; v.1.14) view with parameter -Shb, and all of the other parameters set to default. BAM files were sorted and duplicates were marked using the Picard v.2.6.26 SortSam tool with the following parameters: CREATE_INDEX=true, SORT_ORDER=coordinate, VALIDATION_STRINGENCY = STRICT, and all others set to default; and MarkDuplicates with the parameter REMOVE_DUPLICATES=true, and all others set to default. The final BAM files were then indexed using samtools v.1.14 index with all of the parameters set to the default.

#### Somatic mutation calling using bulk data

Somatic mutations were called from WES using the Somaticwrapper pipeline v.1.6 (https://github.com/ding-lab/somaticwrapper), which includes four different callers, that is, Strelka (v.2.9.10)^56^, MUTECT (v.1.1.7)^57^, VarScan (v.2.3.8)^58^ and Pindel (v.0.2.5)^59^. We kept the exonic single-nucleotide variants (SNVs) called by any two callers among MUTECT v.1.1.7, VarScan v.2.3.8 and Strelka v.2.9.10, and indels called by any two callers among VarScan v.2.3.8, Strelka v.2.9.10 and Pindel v.0.2.5. For the merged SNVs and indels, we applied a 14× and 8× minimal coverage cut-off for tumour and normal, separately. We also filtered SNVs and indels by a minimal variant allele fraction (VAF) of 0.05 in tumours and a maximal VAF of 0.02 in normal samples. We also filtered any SNV within 10 bp of an indel found in the same tumour sample. Finally, we rescued the rare mutations with VAF within 0.015 and 0.05 in ccRCC driver genes on the basis of an established gene consensus list^49^. In a downstream step, Somaticwrapper combines adjacent SNVs into double-nucleotide polymorphisms (DNPs) using COCOON (https://github.com/ding-lab/COCOONS): as the input, COCOON takes a MAF file from a standard variant calling pipeline. First, it extracts variants within 2 bp windows as DNP candidate sets. Next, if the corresponding BAM files used for variant calling are available, it extracts the reads (denoted as *n*_*t*_) spanning all candidate DNP locations in each variant set and counts the number of reads with all of the co-occurring variants (denoted as *n*_*c*_) to calculate co-occurrence rate (*r*_*c*_ = *n*_*c*_/*n*_*t*_). If *r*_*c*_ ≥ 0.8, the nearby SNVs will be combined into DNPs, and COCOON will update the annotation for the DNPs from the same codon based on the transcript and coordinate information in the MAF file. Finally, we rescued the rare mutations with VAF of [0.015, 0.05) in cancer driver genes based on the aforementioned gene consensus list^49^. Further analysis focused on cancer driver genes reported in the previous publications^12,49,53,60–63^.

#### Tumour-only somatic mutation calling using bulk data

For samples for which paired normal samples were not available, tumour-only somatic variants were called using the Mutect2 (tool from GATK v.4.1.2.0) tumour-only version of the Somaticwrapper pipeline (https://github.com/ding-lab/somaticwrapper/tree/tonly.v1.0) with the GDC panel of normal data (https://gdc.cancer.gov/about-data/gdc-data-processing/gdc-reference-files; gatk4_mutect2_4136_pon.vcf.tar). False positives were filtered out by retaining only variant sites with ≥20× coverage and >3 alternate allele supporting reads with ≥0.1 alternate allele VAF. DNPs were again inferred using COCOON.

#### Manual genotyping

We used bam-readcount for both determining and for manually verifying the *KRAS* mutation status in bulk WES at KRAS hotspots Gly12, Gly13 and Gln61. For each case, we first applied bam-readcount to generate read counts for each of the nine bases (3 codons times 3 bases per codon) at these loci and then calculated VAF values of all KRAS hotspots based on reference and alternative base read counts at each position. The only instance in which variants were not already identified by the Somaticwrapper pipeline was in PDAC. Owing to the well-known high rate of KRAS hotspot mutations in PDAC (>90%), any such mutations detected in PDAC during genotyping are automatically reported^64^.

To identify KRAS hotspot mutation status, we applied our in-house tool scVarScan that can identify reads supporting the reference and variant alleles spanning the variant site in each individual cell by tracing cell and molecular barcode information in each snRNA BAM file. For mapping, we used the Memorial Sloan Kettering Cancer Center Hotspots website (https://www.cancerhotspots.org) to obtain the most common KRAS hotspot mutations at Gly12, Gly13 and Gln61 and followed with scVarScan to detect potential minority KRAS mutations in each sample. For non-PDAC samples, hits were then filtered to high-quality mutant allele counts > 5.

#### CNV calling on bulk whole-exome data

Somatic copy-number variants were called using GATK (v.4.1.9.0)^65^. Specifically, the hg38 human reference genome (NCI GDC data portal) was binned into target intervals using the PreprocessIntervals function, with bin-length set to 1,000 bp and using the interval-merging-rule of OVERLAPPING_ONLY. A panel of normals (PON) was then generated using each normal sample as an input and the GATK functions CollectReadCounts with the argument --interval-merging-rule OVERLAPPING_ONLY, followed by CreateReadCountPanelOfNormals with the argument --minimum-interval-median-percentile 5.0. For tumour samples, reads that overlapped the target interval were counted using the GATK function CollectReadCounts. Tumour read counts were then standardized and de-noised using the GATK function DenoiseReadCounts, with the PON specified by --count-panel-of-normals. Allelic counts for tumours were generated for variants present in the af-only-gnomad.hg38.vcf according to GATK best practices (variants further filtered to 0.2 > af > 0.01 and entries marked with ‘PASS’) using the GATK function CollectAllelicCounts. Segments were then modelled using the GATK function ModelSegments, with the denoised copy ratio and tumour allelic counts used as inputs. Copy ratios for segments were then called on the segment regions using the GATK function CallCopyRatioSegments.

Bedtools^66^ intersection was used to map copy-number ratios from segments to genes and assign the called amplifications or deletions. For genes overlapping multiple segments, a custom Python script was used to call that gene as amplified, neutral or deleted based on a weighted copy-number ratio calculated from copy ratios of each segment overlapped, the lengths of the overlaps and the *z*-score threshold used by the CallCopyRatioSegments function. If the resulting *z*-score cut-off was within the range of the default *z*-score thresholds used by CallCopyRatioSegments (v.0.9,1.1), then the bounds of the default *z*-score threshold were used instead (replicating the logic of the CallCopyRatioSegments function).

To map copy-number ratios from segments to chromosome arms, another script was used according to the same approach to then call that chromosome arm as amplified, neutral or deleted. Due to the increased read depths associated with ccRCC and GBM, the PON used for GBM and ccRCC samples was composed exclusively of all of the normal samples from those cancers. The PON used for all other cancer types was compiled from all the normal samples across those remaining cancer types.

#### Sequencing read alignments and quality control of sc/snRNA-seq data

To process sequenced sc/snRNA-seq samples, Cell Ranger (v.6.0.2) from 10x Genomics (with Count functionality) was used for aligning reads to the prebuilt GRCh38 genome reference v.2020-A (refdata-gex-GRCh38-2020-A). The resulting gene-by-cell unique molecular identifier (UMI) count matrix was used by the R package Seurat (v.4.0.5)^67^ for subsequent processing. Paired samples were required to be from the same tissue piece as an snATAC-seq sample and they were generated from single nuclei, with the exception of MM samples, which were generated from single cells. Processed sc/snRNA-seq samples were selected, provided that they met the filtering criteria detailed below. The CellRanger report from each sample was then carefully evaluated and we included samples with no critical errors or warnings. Examples of errors for which samples were excluded were as follows: ‘Error: low fraction reads confidently mapped to transcriptome’ or ‘Error: GEX reads mapping to transcriptome is low’. Furthermore, samples were excluded for less than 700 median genes per cell (except in certain cases in which a high number of cells was detected).

Quality filters were applied to the data to remove barcodes that fell into any of the following categories: possible debris with too few genes expressed (<200) and too few UMIs (<1,000), possible more than one cell with too many genes expressed (>10,000) and too many UMIs (>80,000), possible dead cell or a sign of cellular stress and apoptosis with too high proportion of mitochondrial gene expression over the total transcript counts (>10%) and cells predicted to be doublets by Scrublet, as described below. The cut-offs for these filters were based on recommendations in the Seurat package documentation.

#### Normalization, feature selection, dimensionality reduction and clustering of sc/snRNA-seq data

The filtered gene-count matrix was scaled and normalized for sequencing depth using Seurat’s ‘SCTransform’ function (with the parameters: vars.to.regress = c(“nCount_RNA”, “percent.mito”), return.only.var.genes = F, and all others set to default). From this, the principal components were calculated using the Seurat RunPCA function. Cells were clustered using a graph-based clustering (default of Seurat) approach. First, we used the Seurat function FindNeighbors to embed cells in a *k*-nearest neighbour graph structure, based on the Euclidean distance in principal component analysis (PCA) space, with edges drawn between cells having similar expression patterns. We used the previously defined first 30 principal components (PCs) as inputs to the function, while other parameters were left as defaults. To cluster cells, we then applied modularity optimization techniques (using the default Louvain algorithm from the Seurat function FindClusters) to iteratively group cells together to optimize the standard modularity function. We set the resolution parameter at 0.5, while other parameters were left as defaults.

#### Identification of doublets in sc/snRNA-seq

For the removal of doublets, the Python package Scrublet v.0.2.3 was used to identify doublets from the filtered cellranger gene-by-cell UMI count matrix. The initial Scrublet object was generated from the filtered CellRanger gene-by-cell UMI count matrix using the Scrublet function (parameters: expected_doublet_rate=0.15 and random_state set to a random integer between 0 and 1,000,000). Cells were assigned doublet scores 10 times for each individual sample using the scr.scrub_doublets function (parameters: min_counts=2, log_transform=True). In the final iteration, the random_state of the scrublet object is set to 0. After each iteration, cells are clustered on doublet scores using the KMeans object (with parameters: n_clusters=2, init=‘k-means++’, n_init=10, and max_iter=10000) and fit_predict method using the Python package sklearn v.0.24.2. The boundary between the doublet and singlet clusters after all iteration is then averaged to determine the final doublet cut-off, which is then used by the Scrublet call_doublets function to predict a cell’s doublet status.

#### Merging of sc/snRNA-seq data across samples

Cancer-cohort-level objects were generated using the Seurat function merge to combine sc/snRNA-seq samples objects after quality control. Barcodes annotated as doublets were removed from all of the samples before merging. Once merged, objects were normalized using the Seurat SCTransform function with the same parameters as when normalizing individual objects. Cells were then clustered using the top 50 PCA dimensions using the FindNeighbors and FindClusters functions with Resolution=0.5. To generate the merged pan-cancer objects (containing all cell types, or tumour and selected normal cell types; Extended Data Fig. 2c,f), the same steps were taken starting from the cancer cohort objects. To make a merged object with all cell types, 600 cells were randomly sampled for each cohort-level cluster, and the resulting set of cells was used for merging. The resulting normalized matrices of merged objects were used for subsequent analysis.

#### Cell type annotation of sc/snRNA-seq data

We curated from the literature a list of well-known markers (Supplementary Table 1e and Supplementary Fig. 2c–f). Using the integrated sc/snRNA-seq data of all cells from each cancer type at a time, we filtered the marker genes down to those that were expressed in at least 5% of at least one cluster. We then labelled each cluster with cell type names by examining the expression values and the percentages expressed of all the filtered marker genes across all clusters (using the Dotplot function of the Seurat package). Finally, we also validated cancer cell type annotation using inferCNV results (Supplementary Fig. 2b). Detailed normal epithelial cell annotation was also performed using the expression of known markers (see normal pancreas cell, normal colon cell and normal kidney cell markers in Supplementary Table 1e). First, we isolated and reclustered normal epithelial cells from the PDAC, CRC and ccRCC cancer types, then we evaluated the expression of marker genes across clusters (using the Dotplot function of the Seurat package).

#### BRCA sample basal and non-basal subtype annotation

It was previously reported that BRCA samples of basal and non-basal subtypes tend to have different chromatin accessibility landscapes^6^. To annotate samples with basal and non-basal subtypes, we used two methods. First, we checked the expression of PAM50 genes^68^ across tumour cells of each sample using snRNA-seq data (Supplementary Fig. 7). We examined the per-sample expression of those markers using the DotPlot function of the Seurat package and found specific expressions of PAM50 signatures across samples. We next performed correlation analysis based on TF motif accessibility scores from snATAC-seq data. For this, we used TF scores averaged across cancer cells of each sample (Supplementary Fig. 8). We observed two clusters of samples, corresponding to samples of basal and non-basal subtypes. These two orthogonal approaches produced similar results, and we used the resulting annotation to separate BRCA samples into basal and non-basal groups.

#### InferCNV analysis

To detect large-scale chromosomal CNVs using sc/snRNA-seq data, inferCNV v.0.99.7 was used with the default parameters recommended for 10x Genomics data. InferCNV was run at the sample level and only with post-quality-control filtered data using the raw counts matrix. To run inferCNV on all samples, it was necessary to set ‘ulimit -s unlimited’ in the bash environment followed by defining options (expressions=500000) within R. For specific samples, when the hidden Markov model did not converge with these changes, it was necessary to use inferCNV v.1.11.2. Once finished, copy ratio calls for all genes in a cell were gathered from the hidden Markov model outputs. For snATAC-seq inferCNV was run in an identical manner as to the sc/snRNA-seq calls using the filtered gene accessibility by cell matrix.

#### scVarScan mutation mapping

We applied an in-house tool called scVarScan, which can identify reads supporting the reference allele and variant allele covering the variant site in each individual cell by tracing cell and molecular barcode information in a sc/snRNA-seq BAM file. The tool is freely available at GitHub (https://github.com/ding-lab/10Xmapping). For mapping, we used high-confidence somatic mutations from WES data.

#### Identifying differentially expressed genes using sc/snRNA-seq data

To perform differential expression analysis, we used the FindMarkers function from the Seurat package with default Wilcoxon rank-sum testing. For all DEG analysis, we used a merged object containing selected normal and cancer cells from all cancers. First, to identify tissue- and cancer-cell specific DEGs, we compared cancer cells from each tumour type to the combined set of cancer cells from all other tumours. We specified the following parameters: min.pct=0.1, min.diff.pct=0, logfc.threshold=0 and only.pos=T. Next, to identify cancer cell-specific DEGs, we performed the comparison between cancer cells and their closest normal cell type (Fig. 1c) for each cancer. For this analysis, we used cancer cells from primary tumours only. Moreover, we specified the following parameters: min.pct=0.05, min.diff.pct=0, logfc.threshold=0 and only.pos=F. Finally, to identify metastasis-associated DEGs, we compared neoplastic cells from primary tumours versus neoplastic cells from metastatic tumours from four cohorts used in the analysis (CRC, PDAC, SKCM and UCEC). The following parameters were specified: min.pct=0.1, min.diff.pct=0, logfc.threshold=0 and only.pos=F. For all DEG analysis, Bonferroni correction was applied for *P* value adjustment using all genes from each comparison, and DEGs were considered to be significant if they had an adjusted *P* < 0.05.

#### Gene regulatory network analysis using SCENIC

To infer gene regulatory networks, we used the SCENIC pipeline pySCENIC command line interface version (v.0.11.2)^35^. We ran SCENIC on an SCT-normalized assay of sampled sc/snRNA-seq merged object, 200 cells sampled randomly per cell type of each sample. For the first step we used the GRNBoost2 method, as it is suggested for large scale datasets. For the input, we provided the list of unique TFs that are present in JASPAR2020 db^69^. Steps 2 and 3 of regulon prediction were run with the default parameters using the RcisTarget hg38_refseq-r80 v.9 gene-motif ranking databases (10 kb around the TSS, and 500 bp around the TSS). As the first step of the SCENIC pipeline is using a stochastic gradient boosting algorithm, it is suggested by the developers to run it multiple times and to filter TFs and their targets to those that were reported across multiple iterations^70^. Consequently, we ran the SCENIC pipeline ten times and filtered TFs and their targets to those that appeared in at least 80% of SCENIC runs. We then recalculated AUC scores for the resulting regulons using the AUCell (v.1.19.1) R package. Finally, we filtered regulons to those that contain at least 20 target genes. By using this approach, we were able to obtain a more stable set of regulons that are active in our dataset.

To prioritize regulons for Fig. 3a, we first conducted a differential analysis comparing neoplastic cells from each cancer versus neoplastic cells from all other cancers (primary tumours only), using regulons’ AUC scores. For this, we used a two-sided Wilcoxon test, and the resulting *P* values were adjusted using the Benjamini–Hochberg FDR method. We selected regulons that were both significant (FDR < 0.05) and that also met the following criteria: fold change between the two groups of greater than 1.5 and the mean score in cell group 1 exceeding the median of mean scores across all cell groups. The top 10 such regulons with the highest fold change were selected in each cancer type. If there were less than 10 regulons that passed these criteria, then all regulons were taken for that cancer type. We also added the following regulon–cancer pairs that were supported by comparing TF scores for the same cells’ group analysis using snATAC-seq data: KLF6 in PDAC, NRF1 in GBM, RARA in BRCA (non-basal), MXI1 in ccRCC, E2F7 in GBM and ELF3 in PDAC. Next, to annotate regulons as tissue- or cancer-cell-specific, we performed differential regulon analysis between tumour cells and their CNCs for each cancer type. For this, we used a two-sided Wilcoxon test, and the resulting *P* values were adjusted using the Benjamini–Hochberg FDR method. The regulon was annotated as cancer-cell-specific if FDR < 0.05, the difference in scores between these groups was >0.01 and log_2_[FC] > 0.1.

#### Pathway analysis using sc/snRNA-seq data

For the analysis of the pathways’ activities across regulon gene targets (Extended Data Fig. 6e), we used the sets of genes per tissue- and cancer-cell-specific regulons that were also DEGs in the same cancer (Supplementary Table 2b). We then calculated pathway activity scores using the Jaccard index between the sets of resulting regulons’ targets and the sets of genes from hallmark MSigDB^71^ pathways. We further performed over-representation analysis using hypergeometric test from the fgsea R package. *P* values were adjusted using Benjamini–Hochberg FDR correction.

#### Sequencing read alignments of snATAC-seq and snMultiome-seq

To process sequenced snATAC-seq and snMutiome-seq data, we used the CellRanger-atac count (v.2.0, 10x Genomics) and CellRanger-arc count (v.2.0, 10x Genomics) pipelines, respectively. These pipelines filter and map snATAC-seq reads and identify transposase cut sites, and the CellRanger-arc pipeline also performs filtering and alignment of snRNA-seq reads. The GRCh38 human reference was used for the read mapping (refdata-cellranger-arc-GRCh38-2020-A-2.0.0). Owing to low snRNA-seq quality, the snATAC-seq part of some snMultiome-seq samples was separately run with the modified version of CellRanger-atac v.2.0, which had ATAC cell barcodes replaced with snMultiome-seq barcodes. In particular, the snMultiome-seq barcode file cellranger-arc-2.0.0/lib/python/atac/barcodes/737K-arc-v1.txt was copied into CellRanger-atac directory cellranger-atac-2.0.0/lib/python/barcodes/ and renamed to 737K-cratac-v1.txt. The CellRanger report from each sample was carefully evaluated and we excluded samples with few errors, except the ‘Number of cells is too high’ error, while retaining samples with no errors or with just warnings. Examples of errors for which we removed samples are as follows: ‘ATAC high-quality fragments in cells is low’, ‘ATAC TSS enrichment is low’ and ‘ATAC fragments in peaks is low’.

#### Peak calling for snATAC-seq data

To call peaks on snATAC-seq data (from regular snATAC-seq and from snMultiome-seq), we used the MACS2 tool (v.2.2.7.1)^72^ through the CallPeaks function of the Signac package (v.1.3.0, https://github.com/timoast/signac). We further removed peaks from the Y chromosome, as well as those overlapping genomic regions containing ‘N’. All peaks were resized to 501 bp centred at the peak summit defined by MACS2. We next performed the iterative removal procedure described previously^6^ to get the set of non-overlapping peaks. In brief, we start with retaining the most significant peak by MACS2 peak score (−log_10_[*q*]), removing all peaks that have direct overlap with it. We repeat this procedure for the remaining peaks, until we have the set of non-overlapping peaks. The resulting sample peak set was used to calculate peak-count matrix using FeatureMatrix from the Signac package, which was also used for downstream analysis.

#### Quality control of snATAC-seq data

Quality-control filtering of the snATAC-seq datasets was performed using functions from the Signac package. Filters that were applied for the cell calling include: 1,000 < number of fragments in peaks < 20,000; percentage of reads in peaks > 15; ENCODE blacklist regions percentage < 0.05 (https://www.encodeproject.org/annotations/ENCSR636HFF/); nucleosome banding pattern score < 5; and enrichment-score for Tn5-integration events at transcriptional start sites > 2. Open chromatin regions were annotated with the R package ChIPseeker (v.1.26.2)^73^ using transcript database TxDb.Hsapiens.UCSC.hg38.knownGene. The promoter region was specified (−1000,100) relative to the TSS.

#### Normalization, feature selection, dimensionality reduction and clustering of snATAC-seq data

The filtered peak-count matrix was normalized using term frequency-inverse document frequency (TF-IDF) normalization implemented in the Signac package. This procedure normalizes across cells, accounting for differences in coverage across them and across peaks, giving higher values to the rarer peaks. All peaks were used as features for dimensional reduction. We used the RunSVD Signac function to perform singular value decomposition on the normalized TF-IDF matrix, a method that is also known as latent semantic indexing (LSI) dimension reduction. The resulting 2:30 LSI components were used for nonlinear dimensionality reduction using the RunUMAP function from the Seurat package. The nuclei were clustered using a graph-based clustering approach implemented in Seurat. First, we used the Seurat function FindNeighbors to construct a shared nearest neighbour graph using the 2:30 LSI components. We next used the FindClusters function to iteratively group nuclei together while optimizing modularity using the Louvain algorithm.

#### Quality control, normalization, feature selection, dimensionality reduction and clustering of snMutiome-seq data

For snMultiome-seq data containing profiles of both snRNA- and snATAC-seq data, we first performed separate processing and filtering of cells using the same steps as were described for the processing of separate sc/snRNA-seq and snATAC-seq assays. To obtain the final list of barcodes, we retained the cells that passed the quality control filters in both the snRNA- and snATAC-seq assays. In the result, we obtained filtered gene- and peak-count matrices for the same set of cells. We then performed TF-IDF normalization of the peak-count matrix, followed by LSI dimensionality reduction using the RunTFIDF and RunSVD Signac functions. For normalization and dimensionality reduction of the gene-count matrix, we used the SCTransform and RunPCA functions of Seurat with the same parameters as used for regular sc/snRNA-seq data processing.

We next computed the weighted nearest neighbour (WNN) graph with the FindMultiModalNeighbors function using both data modalities. We used 1:30 PCA components from snRNA-seq and 2:30 LSI components from snATAC-seq for this analysis. We performed nonlinear dimensionality reduction of the resulting WNN graph using the RunUMAP function of Seurat. Finally, we obtained clusters with the FindClusters function using the WNN graph, setting the argument algorithm = 3 (SLM).

#### Identification of doublets in snATAC-seq and snMultiome-seq samples

To identify doublets in snATAC-seq data, we used the Python package Scrublet v.0.2.3 on the filtered cellranger peak-by-cell UMI count matrix. The processing steps were the same as for doublet identification in sc/snRNA-seq. To assign doublets for snMultiome-seq barcodes, we performed doublet identification separately on the filtered CellRanger peak-by-cell and gene-by-cell UMI count matrices. We annotated a barcode as a doublet if it was identified as a doublet by using both assays.

#### Merging of snATAC-seq data across samples (cohort-level objects)

To create snATAC-seq cohort-level merged objects, functions from the Signac and Seurat packages were used. To normalize peak significance scores across samples and cancers, we converted MACS2 peak scores (−log_10_[*q*]) to a score per million as described previously^6^. To get the set of peaks for merging, we first combined peaks from all of the samples for each cohort separately. For overlapping peaks in each cohort, we performed an iterative removal procedure, the same as was used for creating individual sample peak sets, using normalized peak scores as described above. Using this procedure, we obtained the cancer-type-level peak sets. To gain the pan-cancer set of non-overlapping peaks, we renormalized peak scores using the score per million normalization procedure described above and performed the same iterative removal procedure for the combined cohort-level peak set from all 11 cancer types. The resulting list of pan-cancer peaks was quantified in each cohort using the FeatureMatrix Signac function, so that the resulting peak-cell matrices had the same set of features in all of the samples processed.

To merge snATAC-seq datasets, the merge function of the Seurat package was used. We next performed TF-IDF normalization and LSI-dimensionality reduction using the RunSVD function from the Signac package. Non-linear dimensionality reduction was performed using the RunUMAP function with 2:50 LSI components. For analysis involving CNC, we also created two additional merged objects (HNSCC-CESC/AD and UCEC/OV), so that they contain CNC for the HNSCC and OV cohorts, respectively.

#### Merging of snATAC-seq data across cancers (pan-cancer-level objects)

For the analysis involving comparisons between cancers, we aimed to create a pan-cancer-level merged object. To reduce the computational complexity, we subsetted tumour and selected normal cell types for each cohort that can be the putative cell-of-origin: luminal mature and luminal/basal progenitors for BRCA; oligodendrocytes, OPC and astrocytes for GBM; acinar and islet for PDAC; ciliated and secretory endometrial epithelial cells from UCEC; and other normal epithelial cells from all cohorts where they were available, and normal B cells from the MM cohort (Extended Data Fig. 2b,e). We further randomly sampled 1,000 cells for each cohort-level cluster for this. We next used a merge procedure, followed by TF-IDF normalization and LSI dimensionality reduction using Seurat and Signac package functions. For nonlinear dimension reduction with the RunUMAP function, we used 2:150 LSI components. The resulting merged object normalized peak by cell matrix was used in the pan-cancer analysis and in the analysis of TF motif accessibility differences (Extended Data Fig. 2b,e). We also made another merged object to compare chromatin-accessibility profiles across broad cell groups. To make this object, 600 cells were randomly sampled for each cohort-level cluster, and the resulting set of cells was used for merging, applying the same processing steps as described for the processing of the first pan-cancer merged object (Extended Data Fig. 2a,d).

#### Cell type annotation of snATAC-seq and snMultiome-seq data

For snMultiome-seq samples, cell labels were taken directly from snRNA-seq sample annotations. For regular snATAC-seq, the cell types of samples were first annotated with cell type label transfer using functions from Signac and Seurat. First, we quantified chromatin accessibility associated with each gene by summing the reads overlapping the gene body and its upstream region of 2 kb, therefore creating the gene by cell matrix. Coordinates for the genes were used from the Ensembl database v.86 (EnsDb.Hsapiens.v86 package). We next performed log-normalization of the resulting matrices using the NormalizeData function. The integration of paired snATAC-seq and sc/snRNA-seq datasets was performed using the FindTransferAnchors function with the canonical correlation analysis option for the dimensionality reduction. We then used the TransferData function to transfer cell type labels from the sc/snRNA-seq dataset to the snATAC-seq dataset using the obtained set of anchors from the previous step. The cell types were then re-evaluated at the cancer-type-merged object level, where, for each cluster, the cell type label was assigned by the most abundant cell type in that cluster. Cancer cell type annotation was also validated using inferCNV results (Supplementary Fig. 2b). Detailed normal epithelial cell type annotation was performed in sc/snRNA-seq space first. Then, for snMultiome-seq samples, cell labels were directly taken from snRNA-seq annotation and, for regular snATAC-seq samples, cell types were annotated with cell type label transfer using functions from Signac and Seurat.

#### Inference of closest normal cell type by tumour–normal association analysis

We set out to determine the CNCs for 10 cancer types (all except MM due to the lack of resolution, see below) that contained sufficient numbers of cells from 2 to 7 normal tissue cell types per cancer type. We divided the BRCA cohort samples based on basal versus non-basal subtypes as the two subtypes were reported to have different cells of origin^74,75^. To determine a CNC for each cancer, we did the following. We used a combined set of tissue- and cancer-specific DACRs (Supplementary Table 2a) or DEGs (Supplementary Table 2b) for snATAC-seq- and snRNA-seq-based calculations, respectively. For the resulting sets of genes and open chromatin regions, we calculated the average expression or accessibility for the pooled cells from the selected normal (potential cell of origin) cell types (Fig. 1c and Extended Data Fig. 4a), and cancer cells from each sample separately. We next calculated the Pearson correlation coefficient between each tumour and selected normal cell types from its tissue. Both data types produced similar patterns (Fig. 1c and Extended Data Fig. 4a), and we considered the cell type to be a CNC if it had a higher median of correlation coefficients across tumour samples based on snATAC-seq data (Supplementary Table 2d).

On the basis of the above analysis, we defined CNCs for 10 cancers, and they were consistent with those reported in the previous studies: luminal mature cells for BRCA of non-basal subtypes^74^; luminal progenitor cells for BRCA of basal subtype^74,75^; ductal-like-2 cells for PDAC^13,76–79^; distal stem cells for CRC^80^; secretory endometrial epithelial cells for UCEC^81^ and OV; normal squamous cells for HNSCC and CESC; melanocytes for SKCM; proximal tubule cells for ccRCC^82,83^; and OPCs for GBM^84,85^. For MM, we used normal B cells as the CNC^17^. B cells are believed to acquire the initial CNV and structural variants during the class-switch recombination and somatic hypermutation process in a germinal centre. These abnormal B cells are believed to further differentiate into plasma cells and give rise to MM^17^. We have significantly more B cells than normal plasma cells in our dataset and, as the initial tumorigenic events seem to occur in B cells, they were used as the CNC.

#### Identifying DACRs using snATAC-seq data

To perform analysis of differentially DACRs, we used the FindMarkers function of the Signac package (v.1.3) with logistic regression and the fraction of fragments in peaks used as a latent variable to reduce the effect of different sequencing depths across cells. *P-*value adjustment was performed using Bonferroni correction using all peaks in the dataset. We used the same groups of cells that were used for the identification of DEGs in the respective comparisons. To calculate the fold change for all DACRs, we used an improved version of the FoldChange function in the Signac package (v.1.8).

First, to identify tissue- and cancer-cell-specific DACRs, we compared cancer cells from each tumour type to the combined set of cancer cells from all other tumours, using the merged pan-cancer object with cancer and selected normal cells. The following additional parameters were specified for the FindMarkers function: min.pct=0.1, min.diff.pct=0, logfc.threshold=0 and only.pos=T. ACRs that had inconsistent fold change direction between Signac v.1.3 and v.1.8 (*n* = 38) were removed.

Next, to identify cancer cell-specific DACRs (Fig. 1d and Extended Data Fig. 4b), we compared cancer cells from primary tumours with their closest normal cell types (CNCs; Fig. 1c) for each cancer using cohort-level merged objects for 9 out of 11 cancers for which CNCs were available. For HNSCC, we used the CESC/AD-HNSCC merged object that has normal squamous cells for this comparison (CNC for HNSCC) and, for OV cancer, we used the merged UCEC-OV object that had secretory endometrial epithelial cells (CNC for OV). Furthermore, we specified the following parameters for the FindMarkers function: min.pct=0.05, min.diff.pct=0, logfc.threshold=0 and only.pos=F. Furthermore, we wanted to exclude DACRs that were probably affected by CNVs. For this, we annotated DACRs with their closest genes (using the ChIPseeker package, as described above) and then calculated CNV scores for those genes using inferCNV results. CNV scores were calculated as the fraction of cancer cells per cohort that had that gene amplified or deleted (AMP and DEL scores, respectively). We then filtered DACRs using the following criteria: with log_2_[FC] > 0 if AMP > 0.25 and for DACRs with log_2_[FC] < 0 if DEL > 0.25.

Finally, to identify DACRs associated with metastasis, we compared cancer cells from primary tumours to cancer cells from metastatic tumours from 4 cohorts (CRC, PDAC, SKCM and UCEC), using cohort-level merged objects. The following parameters were specified: min.pct=0.01, min.diff.pct=0, logfc.threshold=0 and only.pos=F. To select the DACRs for plotting (Extended Data Fig. 8a), we also calculated the sample-level fold change between cancer cells from each metastatic tumour and pooled cancer cells from all primary tumours of the same cancer. We further prioritized the top 200 DACRs, first by the highest fraction of metastasis samples with a positive fold change and then by the mean fold change across the samples.

#### Pathway enrichment analysis in DACRs

To calculate pathway activity from DACRs (Fig. 4d and Extended Data Fig. 4f), we used cancer-associated pathways from the hallmark gene sets of MSigDB^71^. We used the over-representation analysis function fora from the fgsea package (v.1.24.0) to perform hypergeometric tests with the universe set as all unified peaks detected. DACRs with positive fold changes in each cancer type were selected and then further filtered to have a positive fold change in at least 50% of the samples in the cancer type. To ensure a balanced comparison between cancer types, the number of DACRs used in the analysis is capped at the top 1,000 per cancer type in Extended Data Fig. 4f and the top 5,000 per cancer type in Fig. 4d on the basis of fold change. Each list of DACRs from a cancer type was tested with each gene set and the FDR was calculated for multiple-testing correction. The FDR of each test and the number of genes associated with DACRs in the test is reported in the corresponding bubble plot. The total numbers of DACRs associated with any genes in the gene set across cancer types are reported in the bar plots.

#### Visualizing the coverage of snATAC-seq data

For snATAC-seq coverage plots, we used the CoveragePlot function from the Signac package.

#### Calculation of TF motif scores using snATAC-seq data

To evaluate TF-binding accessibility profiles in the snATAC-seq data, we used the chromVAR tool (v.1.12.0)^86^, which calculates biased-corrected deviations (TF motif scores) corresponding to gain or loss of accessibility for each TF motif relative to the average cell profile. We ran chromVAR using wrapper-functions from the Signac package with the default parameters and the JASPAR2020 database. Mapping of the TF motifs to the DACRs was performed using the motifmatchr R package. To identify TFs with differential activity between cell groups of snATAC-seq data, we used a two-sided Wilcoxon rank-sum test for the whole set of TFs in the assay, subsequently applying FDR correction to the resulting *P* values.

#### Identifying differentially accessible TF motifs using snATAC-seq data

We performed analysis of differentially accessible TF motifs (DAMs) using chromVAR scores for the following comparisons: primary cancer cells from each cancer cohort versus pooled primary cancer cells from all other tumours (Supplementary Table 4d), primary cancer cells versus respective CNC (Supplementary Table 4e) and metastatic cancer cells versus primary cancer cells (Fig. 4a and Supplementary Table 6d). For all DAM analysis, we used scores calculated on merged pan-cancer objects containing cancer and selected normal cells. To perform DAM analysis, we used a two-sided Wilcoxon rank-sum test between the corresponding groups, subsequently applying FDR correction to the resulting *P* values. For metastasis-specific TFs, we also used results of differential regulons obtained from SCENIC (based on sc/snRNA-seq data). Differential regulons were calculated using a two-sided Wilcoxon rank-sum test between the same groups of cells, and then applying FDR correction. We selected only those TFs that were significant (FDR < 0.05) in both the DAM and the regulon analysis, and also required them to have a score change in the same direction between the same cell groups. Finally, we calculated the expression score as the absolute value of expression log_2_[FC] between the metastatic and primary cancer cells using per sample average values, also requiring the same fold change direction as the direction of the score difference for the same TF (Fig. 4a).

#### Annotating genomic regions with *cis-*regulatory elements

Open chromatin regions were annotated with *cis-*regulatory elements from the geneHancer Regulatory Elements Elite list for hg38^87^ from the Genome USCS browser (last updated version, 2 September 2018). Genomic regions of geneHancer enhancers and promoter/enhancers were overlapped with a minimum overlap of 400 bp using the findOverlaps function from the IRanges R package. We also downloaded scEnhancer enhancers from all tissues and overlapped them with open chromatin regions the same way. Moreover, we downloaded interactions between GeneHancer regulatory elements and target genes from geneHancer Interactions Double Elite list (last updated version, 15 January 2019). Region-to-gene links were then annotated by presence in the geneHancer Interactions Double Elite list.

#### Annotating genomic regions with public ChIP–seq datasets

First, we identified each JASPAR2020 TF motif in every open chromatin region using the function matchMotifs from the motifmatchr (v.1.12.0) R-package. We next downloaded ENCODE ChIP–seq hg38 bed files for all available TFs (download date, 28 January 2022). We then overlapped TF-binding ChIP–seq regions with the corresponding TF motif coordinates in our chromatin regions set using the findOverlaps function from the IRanges R package with minimum overlap equal to the length of the motif. If a given TF motif fully overlapped with a ChIP–seq-based binding region of the same TF, then we labelled this motif as being supported by ChIP–seq data.

#### Confirming tissue- and cancer-cell-specific TFs using published chromatin accessibility datasets

We collected the sc/snATAC-seq or bulk ATAC-seq studies with relevant differentially expressed TF analysis from published literature, including BRCA^88^, MM^89^, ccRCC^90^, PDAC^91^, pan-organ chromatin accessibility^8^ and the bulk ATAC-seq study in human cancers^6^. For BRCA, epithelial cells were compared to endothelial cells, fibroblast or immune cells, and the upregulated TFs were identified. In MM, fold change expression of TFs between myeloma and plasma cells was calculated to identify upregulated TFs in myeloma cells. For ccRCC and PDAC, the fold change expression of cancer-specific TFs between cancer cells and normal cells was calculated to identify upregulated TFs in either cancer cells (cancer-specific TF) or normal cells (tissue-specific TF).

Moreover, the sc/snATAC-seq dataset from the pan-organ chromatin accessibility study was used to confirm cancer and tissue-specific TFs. The relevant cell types were annotated with their relevant disease code as the CNCs of the cancer. For example, mammary luminal epithelial cells to non-basal BRCA, mammary basal epithelial to basal BRCA, keratinocyte to HNSCC, colon epithelial cells to CRC, enterocyte to CRC, colon goblet to CRC, SI goblet to CRC, melanocyte to SKCM, acinar to PDAC, ductal cell to PDAC, astrocyte to GBM, oligodendrocyte to GBM, oligo precursor to GBM and plasma cells to MM. The tissue and cancer cell-specific TFs highly expressed in the corresponding cell populations were then identified.

Finally, the bulk ATAC-seq dataset from human cancers^6^ was used to determine whether the tissue and cancer-specific TFs are the markers for the corresponding chromatin-accessibility-driven clusters. The clusters were annotated as follows: Cluster 1 to ccRCC, cluster 2 to CRC, cluster 3 to BRCA, cluster 5 to GBM, cluster 7 to SKCM, cluster 8 to CESC, cluster 14 to BRCA, and cluster 15 to UCEC.

#### CUT&RUN sequencing read alignments, quality control and peak calling

To process the CUT&RUN reads, we first performed quality control using FastQC to assess read quality (http://www.bioinformatics.babraham.ac.uk/projects/fastqc). We next used Trimmomatic^92^. The resulting trimmed reads were subsequently mapped to the human reference genome (GRCh38.d1.vd1.fa.tar.gz) using Bowtie2^93^ with the dovetail setting. Finally, to eliminate any duplicated reads, the aligned reads were processed for duplicate removal using Picard (http://broadinstitute.github.io/picard/). To call peaks, we used MACS2 using an IgG BAM file as a control. Then, for resulting peaks, we applied the same filtering steps as for the peak calling on snATAC-seq data.

#### Direct binding profiling of TFs to target genes using ENCODE ChIP–seq datasets

To comprehensively analyse the TF-specific ChIP–seq datasets from ENCODE, we used SCENIC to obtain the list of target genes for 53 tissue- and cancer-specific TFs with ChIP–seq datasets available. Subsequently, we extracted and aggregated the ChIP–seq peaks from multiple biosamples (Supplementary Table 5b) using the readPeakFile function from ChIPseeker and determined overlaps with the promoter regions of the target genes (5 kb upstream and downstream of TSSs) using the makeBioRegionFromGranges and getTagMatrix functions from ChIPseeker (Supplementary Table 5c). To annotate all of the peaks and regions, we used TxDb.Hsapiens.UCSC.hg38.knownGene in Ensembl style. We also overlapped the snATAC-seq peaks and CUT&RUN peaks with target genes using the aforementioned methods. Finally, we visualized the average ChIP–seq, snATAC-seq and CUT&RUN signals around the TSSs of the target genes using the plotAvgProf function from ChIPseeker, allowing for a comprehensive understanding of the regulatory landscape of the TFs across various tissues, cell lines and cancer types.

#### Bulk ATAC-seq and snATAC-seq comparison

We compared the peak coordinates from bulk ATAC-seq^6^ with the peak coordinates from our snATAC-seq data in eight cancer types, namely UCEC, ccRCC, GBM, BRCA, CESC/AD, CRC, SKCM and HNSCC. For each cancer cohort, common open chromatin regions were defined as the ones that had overlaps of at least 50 bp (overlap > 10%) with any open chromatin regions from the bulk ATAC-seq study, and all other regions (no overlap or overlap < 10%) were identified as snATAC-seq-specific open chromatin regions.

#### Identifying snATAC-seq cell-type-specific peaks

To generate the bar plot showing the snATAC-seq specific peaks that were found in multiple cell types, we first used the AccessiblePeaks function from the Signac package^94^ to identify accessible regions in each cell type of the eight cancer types. We next categorized the identified peaks into two groups on the basis of whether they appeared in only one cell type, such as tumour cells, or whether they appeared in more than one cell type. We then used the BEDtools^66^ intersect function to compare the peak coordinates of snATAC-seq-specific peaks with these two cell type groups of peaks. This analysis enabled us to identify the snATAC-seq-specific peaks that were accessible in multiple cell types, which we then used to generate the bar plot.

#### Overlapping chromatin accessibility peaks and ChIP–seq peaks

To overlap snATAC-seq and ChIP–seq peaks, we used the BEDtools^66^ intersect function to compare the peak coordinates obtained from ChIP–seq^95^ with our snATAC-seq-specific, bulk-ATAC-seq-specific and bulk/snATAC-seq overlapping peaks. This analysis was conducted separately for each of the eight cancer types to determine the extent to which the identified snATAC-seq peaks represented true signals rather than noise.

#### Overlapping snATAC-seq-specific peaks and fetal chromatin accessibility peaks

To investigate whether our snATAC-seq-specific peaks were recurrently observed in other datasets, we compared our peak sets with the cell atlas of fetal chromatin accessibility^9^. We downloaded the master list of sites (GSE149683_File_S1.Master_list_of_sites.txt) and converted the genomic coordinates from hg19 to hg38 using the UCSC liftOver tool. We then converted the bed files to GRanges objects and used the findOverlaps function of GRanges to determine the overlaps between our snATAC-seq-specific peaks and the fetal chromatin accessibility peaks.

#### Linking genomic regions to genes

We applied the LinkPeaks function from the Signac R package (v.1.8.0)^94,96^ on tumour cells with snMultiome-seq data (snRNA-seq and snATAC-seq measured in the same cell). Only open chromatin regions located within 500 kb of a gene TSS were considered. Links were considered to be significant with a correlation value *r* > 0.05 and *P* < 0.05. Furthermore, we followed an established procedure^6^ to account for diffuse correlations. Diffuse correlations occur in genomic regions in which chromatin accessibility is generally high, whereby the gene expression is increased. It does not necessarily relate to an increased accessibility of *cis-*regulatory elements. To account for diffuse correlations, we divided each chromosome into 100 kb windows, quantified accessibility of these regions and correlated this accessibility with expression of genes of which the TSS is within 500 kb. As diffuse 100 kb windows are significantly larger than peaks (501 bp), they have accessibility coverage in more cells and, consequently, receive higher correlation values on average. To mitigate these differences, we *z-*scored correlation values for both open chromatin regions and 100 kb windows. We then compared *z*-scored correlation values and retained only those predicted region-to-gene links that had higher *z*-scored correlation values than the 100 kb window that they belong to. Moreover, we reasoned that copy-number changes are strong drivers of gene expression patterns, so it is important to account for them. We used the results of inferCNV on RNA-assay for snMultiome-seq samples and on ATAC-assay for regular snATAC-seq samples to quantify the number of cells with a gain or amplification of each gene and excluded those genes that are frequently amplified (in >25% of cancer cells and >2,000 cells). For example, on the basis of these thresholds, the *ELF3* gene was excluded in PDAC, but was retained in BRCA, CRC, HNSCC, OV and UCEC.

#### Links associated with cancer transitions

To identify links implicated in cancer transitions from normal cells to primary tumour (Fig. 2d and Extended Data Fig. 5g), we required the following: (1) the ACR in the link to be significantly more accessible (log_2_[FC] > 0.5, Wilcoxon rank-sum test FDR < 0.05) and (2) the gene to be significantly upregulated (log_2_[FC] > 0.25, Wilcoxon rank-sum test FDR < 0.05) in primary cancer cells versus their CNCs.

#### Enrichment of links in TF target genes

We calculated the enrichment of genes linked to ACR containing a TF motif based on the idea that, if a group of genes is indeed regulated by a TF, then we should be able to identify TF-motif-containing genomic regions of which the accessibilities are correlated with expression of these genes. The working model is based on the biology of TF activity. Namely, a TF binds to its binding site near a target gene, indicating the accessibility of this region, and subsequently stimulates the expression of this gene. This relationship between accessibility and gene expression should be detectable by correlation in a similar manner as we showed above. To test whether TF target genes identified by SCENIC analysis are significantly enriched for genes linked to the accessibility of this TF motif, we first sought to characterize the random background rate of gene–ACR linkage occurrences for each regulon by performing 500 samplings of *N* random-picked genes, where *N* is the number of target genes in the regulon, and then identifying the number *K* of genes linked to ACRs containing the TF motif. Random genes were limited to genes expressed in each cancer type and were therefore different for each cancer type. The null hypothesis is taken as the expectation for the number of genes linked to a TF motif, namely the average $E[K]\approx \frac{{\sum }_{i=1}^{500}{K}_{i}}{500}$. Presuming that gene–ACR counts are normally distributed, we then used a Gaussian curve of *μ* = mean(*K*) and *σ* = s.d.(*K*) for testing each respective regulon, computing *z* = (*M* − *μ*)/*σ*, where *M* is the observed number of target genes linked to TF motifs and converting this score to a one-sided *P* value. For visualization purposes, we computed fold change $\frac{M}{{\rm{mean}}(K)}$. Motifs in ACRs were identified using the CreateMotifMatrix function in the motifmatchr R package.

#### Links associated with genetic drivers

To find ACR-to-gene links in cancer driver genes we filtered links by oncogenes from a previous study^49^. For visualization of accessibility and gene expression of *EGFR* in BRCA basal, CESC and HNSCC cancers, we included only samples with *EGFR* copy-number neutral calls from WES using the GATK pipeline (see above). All of the samples except for one HNSCC sample also had neutral inferred copy-number calls for *EGFR* based on inferCNV results from snRNA-seq data. This HNSCC sample with case ID P5514 showed no significant *EGFR* expression difference with WES-based *EGFR*-copy-number neutral cases (log_2_[FC] = 0.08, Wilcoxon rank-sum test *P* value = 0.09). However, all WES-based *EGFR-*amplified cases showed significant upregulation of *EGFR* expression compared with *EGFR*-copy-number neutral cases (P5504, log_2_[FC] = 0.13, *P* = 3.7 × 10^−6^; P5216, log_2_[FC] = 2.2, *P* = 1.7 × 10^−39^; P5379, log_2_[FC] = 1.3, *P* = 4.6 × 10^−81^; P5576, log_2_[FC] = 3.9, *P* = 9.3 × 10^−61^). As the GATK pipeline did not call *EGFR* copy-number gain in P5514 and we did not observe upregulation of *EGFR* expression in this case, we included it in Fig. 5c.

#### HPV status assignment

To detect HPV reads in the sample, we followed a series of steps. First, we constructed a genome database for known HPV genotypes. We next extracted the unmapped reads from the snRNA-seq BAM files that did not align to the human genome. We then used BWA^55^ to align these unmapped reads against the constructed virus genome database. Finally, we identified the HPV reads from the alignment results. Detailed source code for this process can be found at the GitHub repository (https://github.com/ding-lab/VirusScan/tree/simplified).

#### Survival analysis

RNA-seq expression data for TCGA samples were obtained through the cBioPortal (https://www.cbioportal.org/), along with clinical information from the TCGA Pan-Cancer Clinical Data Resource (TCGA-CDR)^97^. The regulons generated in this study (see the ‘Gene regulatory network analysis using SCENIC’ section) were used to calculate regulon activity on the basis of bulk RNA-seq expression data for samples from TCGA cohorts (HNSCC, GBM, READ, COAD and PAAD) using the AUCell (v.1.19.1) R package (Supplementary Table 9a).

Samples were grouped on the basis of regulon activity scores: those with scores of higher than the median as the ‘high group’ and those with scores ≤median as the ‘low group’. The survival probability of progression-free survival/overall survival and Kaplan–Meier curves were then calculated for both groups using the survival (v.3.2.7) and survminer (v.0.4.9) R packages. We also performed Cox proportional hazard models to discern the regulons that most significantly and independently influenced patient survival. Significant regulons identified from Kaplan–Meier curves were added to the models to ascertain their distinct contribution to survival, after adjusting for age, sex and HPV status.

#### Identifying regulons significantly associated with HPV infection

To assess differences in regulon activity between HPV^+^ and HPV^−^ HNSCC samples, we used the Wilcoxon rank-sum test for comparisons to identify HPV-status-associated regulon changes. We further validated the HPV-status-associated regulons in the TCGA-HNSCC cohort using the calculated regulon scores (Supplementary Table 9a).

#### Making case-level objects of paired primary/metastatic samples for EMT analysis

In our cohort, we had nine patient cases with both primary and metastatic tumours in UCEC and CRC cancers. For the EMT analysis, we created case-level snATAC-seq objects, including cancer cells from a primary sample, cancer cells from a metastatic sample and normal epithelial cells from a primary sample (if available). These cells were renormalized and clustered similar to the approach described above (using 2:30 LSI components for the runUMAP and FindNeighbors functions). Clustering resolution was adjusted per case to prevent overclustering. For UCEC cases CPT1541DU and CPT704DU, the resolution was 0.2, for CPT2373DU and CPT4096DU it was set to 0.1, and for CPT4427DU, it was 0.3. For CRC cases CM1563C and CM663C, the resolution was 0.1 and, for CM268C and CM618C, it was 0.2. Few cases showed small clusters of cells with increased accessibility of immune markers, suggesting that they are probably doublets with immune cells; they were therefore marked as other and were not included in the downstream analysis.

#### Trajectory inference for EMT analysis of paired primary/metastatic samples

For the trajectory analysis of the nine paired primary/metastatic snATAC-seq samples, we used the slingshot R-package (v.2.5.1), which implements a top performing trajectory inference method in a large trajectory inference benchmark^98,99^. Slingshot requires two inputs: dimensionality reduced data and a clustering of cells. For the clustering of cells, we used the cell type annotations (normal, primary tumour and metastatic tumour) in all cases as input, specifying the normal cluster as the starting cluster or primary tumour if normal cells were not available in the sample. For the dimensionality reduction, we used a supervised method known as between cluster analysis (BCA), which uses cell type information in addition to the underlying data to reduce the data in a way that is more amenable to trajectory inference by better preserving the relationships between cell types. Concretely let $X\in {R}^{nxp}$ denote an snATAC-seq measurement where *n* is the number of cells and *p* is the number of peaks and a clustering of the cells $C=\{{C}_{1},{C}_{2},...,{C}_{K}\}$ into *K* cell types. Let ${\mu }_{k}=\frac{1}{| {C}_{k}| }{\sum }_{i\in {C}_{k}}{X}_{i}\in {R}^{1xp}$ denote the centroid of cluster *C*_*k*_ and define the between-cluster variance *V*_*B*_ as ${V}_{B}=tr({\sum }_{k=1}^{K}| {C}_{k}| {{\mu }_{k}}^{T}{\mu }_{k})=tr({S}_{B}(X,C))$, where ${S}_{B}(X,C)\in {R}^{pxp}$ is known as the between cluster scatter. The objective of BCA is to find a set of *r* = *K* − 1 orthogonal axes held in the columns of $W\in {R}^{pxr}$ that best preserve the between cluster variance; concretely, BCA solves the following optimization problem $\mathop{\max }\limits_{{W}^{T}W={I}_{r}}tr({W}^{T}{S}_{B}(X,C)W)$. The solution (optimal W*) is given by the largest *r* eigenvectors of *S*_*B*_(*X*,*C*), with the corresponding BCA embedding corresponding to *Y*_*BCA*_ = XW*. For every case, we gave Slingshot the first two components of BCA as input (we found in practice that this performed the best qualitatively as opposed to using all *K* − 1 components). As the between-cluster variance is a supervised statistic (requires knowledge of a cell type clustering), it preserves relationships between cell types, which is desirable for trajectory inference. Note that BCA works only for datasets with three or more clusters when wanting to produce a visualization or input to a trajectory inference method.

To control for the confounding effects of total read count, we gave as input to BCA the 2:50 LSI components and either the cell type annotation (normal, primary or metastatic cancer cells) or the case-level clusters (Extended Data Fig. 8b). We gave BCA the cell type annotations as the clusters when there were normal cells present in the sample (*K* = 3); otherwise, we used the case-level clusters. Moreover, both case CM1563C and case CPT2373DU had only two cell type clusters (primary and metastasis) and two or fewer case-level clusters; we therefore gave Slingshot as input the 2:50 LSI components for CM1563C and CPT2373DU, as BCA returns only a one-dimensional embedding for both of these samples.

Slingshot outputs a pseudotime (a real number modelling the underlying biological progression) for each cell. For each case we correlated, using Pearson’s coefficient, the pseudotimes with each of the 663 TF motif scores obtained from our previous analysis and adjusted the corresponding *P* values for each correlation using the Benjamini–Hochberg method to control for multiple tests. We used an FDR threshold cut-off of 0.05 (Supplementary Table 7).

#### Identifying enriched metastatic pathways from ACRs

For the nine paired primary–metastatic samples (4 CRC and 5 UCEC), we identified significant pathways that are characterized by DACRs across primary and metastatic cancer cells. We identified two different sets of primary–metastatic DACRs: regions that were significantly associated with TF scores and regions that were significantly associated with pseudotime identified in the trajectory analysis filtered to contain only primary and metastatic cells. The rationale here is that both the pseudotime and the TF motif score are relevant to metastatic progression of primary tumour cells. Thus, regions that are significantly associated with either feature are likely to have a role in the metastasis of tumour cells. We used lasso regression as implemented in the R glmnet package^100^ to identify which peaks were significantly associated with either TF motif score or pseudotime. That is, we used either the pseudotime or the TF motif score as the response variable and assigned all of the chromatin regions as covariates. We chose lasso for two reasons, as opposed to linear regression or ridge regression. First, there are far more peaks than cells; therefore, linear regression cannot be applied. Second, we wanted a sparse set of peaks that are more likely to be part of the metastatic process. We considered peaks to be significantly associated if they have a non-zero lasso regression coefficient. We chose the value of the lasso regularization parameter lambda by performing tenfold cross-validation using cv.glment, subsequently choosing the lambda that minimizes the cross-validation error in all of the samples except in CPT2373DU, for which we saw empirically better results by choosing the minimum lambda across the default glment lambda sequence.

The genes related to the pseudotime DACRs were collected for gene-set over-representation analysis (Extended Data Fig. 9h) using the database of hallmark MSigDB pathways^71^. The significant pathways were obtained by running a hypergeometric test using clusterProfiler listing the pathways with a varied range of FDRs. We performed an identical analysis for the DACRs associated with the activities of TFs involved in metastasis (Supplementary Fig. 5).

### Reporting summary

Further information on research design is available in the Nature Portfolio Reporting Summary linked to this article.

## Online content

Any methods, additional references, Nature Portfolio reporting summaries, source data, extended data, supplementary information, acknowledgements, peer review information; details of author contributions and competing interests; and statements of data and code availability are available at 10.1038/s41586-023-06682-5.

### Supplementary information

Supplementary Notes 1–8 Supplementary Figs. 1–8, full captions for Supplementary Tables 1–9 and Supplementary References.

Reporting Summary

Supplementary Table 1 Dataset overview

Supplementary Table 2 Tissue- and cancer-specific DEGs/DACRs identified in this study.

Supplementary Table 3 ACR-to-gene links in PDAC and BRCA cohorts connecting a DACR and a DEG.

Supplementary Table 4 Tissue- and cancer-cell-specific regulons and TFs identified in this study.

Supplementary Table 5 Confirming tissue- and cancer-cell-specific TFs using published datasets.

Supplementary Table 6 Identifying DACRs, DEGs, TFs and regulons associated with metastasis.

Supplementary Table 7 Correlation of TF motif scores with pseudotime.

Supplementary Table 8 Impact of genetic drivers.

Supplementary Table 9 Regulons associations with clinical features.

## Data Availability

Sequencing data are part of Human Tumour Atlas Network (HTAN) dbGaP Study accession phs002371.v3.p1 and Clinical Proteomic Tumour Analysis Consortium (CPTAC) dbGaP Study accession phs001287.v17.p6. Data can be accessed through the HTAN DCC Portal (https://data.humantumoratlas.org/) under the HTAN WUSTL Atlas. Sequencing data for CPTAC ccRCC and GBM samples are available through the NCI Genomic Data Commons (GDC) under the CPTAC3 project. Matrices for CPTAC GBM and ccRCC samples and CUT&RUN data are available from the Gene Expression Omnibus (GEO) under accession numbers GSE240822 and GSE240699, respectively. GRCh38 references used for sc/snRNA-seq (refdata-gex-GRCh38-2020-A) and snATAC-seq and snMultiome-seq (refdata-cellranger-arc-GRCh38-2020-A-2.0.0) analyses are freely available from the 10x Genomics website (https://support.10xgenomics.com). The reference GRCh38 genome (https://api.gdc.cancer.gov/data/254f697d-310d-4d7d-a27b-27fbf767a834) used for WES and CUT&RUN read alignment is available from GDC (https://gdc.cancer.gov/about-data/gdc-data-processing/gdc-reference-files).

## Code availability

Many bioinformatics tools were used in the course of this work. All tools written and/or published by the authors are freely available at our public GitHub repository (https://github.com/ding-lab/), including the somaticwrapper variant calling pipeline (https://github.com/ding-lab/somaticwrapper), the code for the mutation mapping from bulk to single cells (https://github.com/ding-lab/10Xmapping), the pipeline for HPV status assignment (https://github.com/ding-lab/VirusScan/tree/simplified) and COCOON (https://github.com/ding-lab/COCOONS). Other tools used here are available from their associated authors. The scripts and additional information for reproducing analysis and figures are available at our GitHub page (https://github.com/ding-lab/PanCan_snATAC_publication).
