# Supplementary material for: Epigenetic regulation during cancer transitions across 11 tumour types
Source: Nature. 2023 Nov 1;623(7986):432–41. doi: 10.1038/s41586-023-06682-5 (PMC10632147; doi:10.1038/s41586-023-06682-5)
Supplement: Supplementary file 1 — Supplementary Figs. 1–8, full captions for Supplementary Tables 1–9 and Supplementary References. [file 41586_2023_6682_MOESM1_ESM.docx]

**Epigenetic Regulation During Cancer Transitions Across 11 Tumor Types**

Nadezhda V. Terekhanova^1,2,*^, Alla Karpova^1,2,*^, Wen-Wei Liang^1,2,*^, Alexander Strzalkowski^3^, Siqi Chen^1,2^, Yize Li^1,2^, Austin N. Southard-Smith^1,2^, Michael D. Iglesia^1,2^, Michael C. Wendl^1,2^, Reyka G. Jayasinghe^1,2^, Jingxian Liu^1,2^, Yizhe Song^1,2^, Song Cao^1,2^, Andrew Houston^1,2^, Xiuting Liu^1^, Matthew A. Wyczalkowski^1,2^, Rita Jui-Hsien Lu^1,2^, Wagma Caravan^1,2^, Andrew Shinkle^1^, Nataly Naser Al Deen^1,2^, John M. Herndon^4,5^, Jacqueline Mudd^4^, Cong Ma^3^, Hirak Sarkar^3^, Kazuhito Sato^1,2^, Omar M. Ibrahim^1,2^, Chia-Kuei Mo^1,2^, Sara E. Chasnoff^4,5^, Eduard Porta-Pardo^6,7^, Jason M. Held^1,5^, Russell Pachynski^1,5^, Julie K. Schwarz^8^, William E. Gillanders^4,5^, Albert H. Kim^5,9^, Ravi Vij^1,5^, John F. DiPersio^1,5^, Sidharth V. Puram^10^, Milan G. Chheda^1,5^, Katherine C. Fuh^11,12^, David G. DeNardo^1,5^, Ryan C. Fields^4,5,#^, Feng Chen^1,5,#^, Benjamin J. Raphael^3,#^, Li Ding^1,2,5,13,#^

*^1^Department of Medicine, Washington University in St. Louis, St. Louis, MO, 63110, USA;*

*^2^McDonnell Genome Institute, Washington University in St. Louis, St. Louis, MO, 63108, USA;*

*^3^Department of Computer Science, Princeton University, Princeton, NJ, 08544, USA;*

*^4^Department of Surgery, Washington University in St. Louis, St. Louis, MO, 63110, USA;*

*^5^Siteman Cancer Center, Washington University in St. Louis, St. Louis, MO, 63110, USA;*

*^6^Josep Carreras Leukaemia Research Institute, Badalona, 08916, Spain*

*^7^Barcelona Supercomputing Center, Barcelona, 08034, Spain*

*^8^Department of Radiation Oncology, Washington University in St. Louis, St. Louis, MO, 63110, USA;*

*^9^Department of Neurological Surgery, Washington University in St. Louis, St. Louis, MO, 63110, USA;*

*^10^Department of Otolaryngology–Head & Neck Surgery, Washington University in St. Louis, St. Louis, MO, 63110, USA;*

*^11^Department of Obstetrics and Gynecology, University of California, San Francisco, San Francisco, CA, 94158, USA;*

*^12^Department of Obstetrics and Gynecology, Washington University in St. Louis, St. Louis, MO, 63110, USA;*

*^13^Department of Genetics, Washington University in St. Louis, St. Louis, MO, 63110, USA;*

*Equal contribution

#Correspondence should be addressed to: [rcfields@wustl.edu](mailto:rcfields@wustl.edu) (R.C.F.), [fchen@wustl.edu](mailto:fchen@wustl.edu) (F.C.), [braphael@princeton.edu](mailto:braphael@princeton.edu) (B.J.R.) and [lding@wustl.edu](mailto:lding@wustl.edu) (L.D.)

**SI guide**

[**Supplementary Note 1. Benchmarking of accessible chromatin regions (ACRs) against public ATAC-seq and ChIP-seq datasets 3**](#_i6i231k60aze)

[Supplementary Figure 1. Benchmarking of accessible chromatin regions (ACRs) against public ATAC-seq and ChIP-seq datasets. 3](#_rm75qsw7qxfg)

[**Supplementary Note 2. Cell type annotation using sc/sn-RNA-seq 4**](#_tl9d5iz9vrd8)

[Supplementary Figure 2. Cell type annotation using sc/sn-RNA-seq. 5](#_tgysvar8odgz)

[**Supplementary Note 3. Tissue- and cancer cell-specific DACRs validation using published snATAC-seq dataset 6**](#_fbxejsjrjwyt)

[Supplementary Figure 3. DACRs validation using published snATAC-seq dataset. 6](#_crw36f0uer1)

[**Supplementary Note 4. Regulon validation using our and published datasets 7**](#_q7wd4ivuygzm)

[Supplementary Figure 4. Tissue- and cancer-specific regulons and TFs. 8](#_umxy6wx15rmw)

[**Supplementary Note 5. TFs associated with pseudotime in 9 cases with paired primary tumor and metastasis samples 9**](#_xt1t221inky7)

[**Supplementary Note 6. Pathway enrichment in ACRs associated with TFs involved in metastasis across 9 cases with paired primary tumor and metastasis samples 9**](#_1l33so7kpk21)

[Supplementary Figure 5. Pathway enrichment analysis in DACRs associated with TFs involved in metastasis. 10](#_wxtkviem0ii1)

[**Supplementary Note 7. Characterization of mutational landscape in 11 cancers 11**](#_we8fxsu1gn58)

[Supplementary Figure 6. Mutational landscape of 11 cancer types. 11](#_kac6ncph4yro)

[**Supplementary Note 8. Limitation of the study 12**](#_lhkv83ijcxqr)

[Supplementary Figure 7. Basal and non-basal subtype annotation using snRNA-seq data. 12](#_1rlolypsi6fx)

[Supplementary Figure 8. Basal and non-basal subtype annotation using snATAC-seq data. 13](#_lqgzxzqk6sdn)

[**Captions for Supplementary Tables 1 to 9 14**](#_tupvqfjq40y7)

[Supplementary Table 1: Dataset overview. 14](#_6ym13br2rrb4)

[Supplementary Table 2: Tissue and cancer-specific DEGs/DACRs identified in this study. 14](#_56c9887w6qr4)

[Supplementary Table 3: ACR-to-gene links in PDAC and BRCA cohorts connecting a DACR and a DEG. 14](#_5fz6ty6i1xxi)

[Supplementary Table 4: Tissue- and cancer-cell specific regulons and TFs identified in this study. 15](#_rvwzizt8nq99)

[Supplementary Table 5: Confirming tissue- and cancer-cell specific TFs using published datasets. 15](#_sv3yr5a2nd3y)

[Supplementary Table 6: Identifying DACRs, DEGs, TFs, and regulons associated with metastasis. 15](#_36zd9fqhbg81)

[Supplementary Table 7: Correlation of TF motif scores with pseudotime. 16](#_tm709ckkqh05)

[Supplementary Table 8: Impact of genetic drivers. 16](#_6b0ni87p84m7)

[Supplementary Table 9: Regulons associations with clinical features. 16](#_g5y7sjmnpo1f)

[**References 17**](#_up2n5mu8dbno)

#

# **Supplementary Note 1. Benchmarking of accessible chromatin regions (ACRs) against public ATAC-seq and ChIP-seq datasets**

We profiled chromatin accessibility of 1.24 million ACRs spanning 618 Mb (19.8%) of the reference human genome, which is comparable to a snATAC-seq atlas of 15 fetal organs[^1^](https://www.zotero.org/google-docs/?7a0SiL). We further benchmarked ACRs with the TCGA pan-cancer bulk ATAC-seq study[^2^](https://www.zotero.org/google-docs/?hapAWX), finding 8-27% of ACRs were shared between the bulk and our dataset, with the majority of ACRs being snATAC-seq unique (**Supplementary Figure 1a**). We investigated these snATAC-seq unique ACRs and found 60-75% and 74-83% of them overlapped with regions identified by ENCODE ChIP-seq data[^3,4^](https://www.zotero.org/google-docs/?PKVZWM) and the fetal pan-organ snATAC-seq data[^1^](https://www.zotero.org/google-docs/?cQDM5h), respectively (**Supplementary Figure 1b-d**). We also reasoned that single cell resolution could result in identification of non-cancer cell ACRs, and indeed snATAC-seq unique ACRs were found in one or multiple cell types of tumor microenvironment (TME) (**Supplementary Figure 1e**). We also observed that small proportions of snATAC-seq unique ACRs (ranging from 0.01% in ccRCC to 23.5% in CESC/AD) were cancer cell unique (**Supplementary Figure 1e**). Taken together, our snATAC-seq dataset provides a large number of reliable ACRs representing both cancer cell-specific and the TME-shared ACRs.

## **
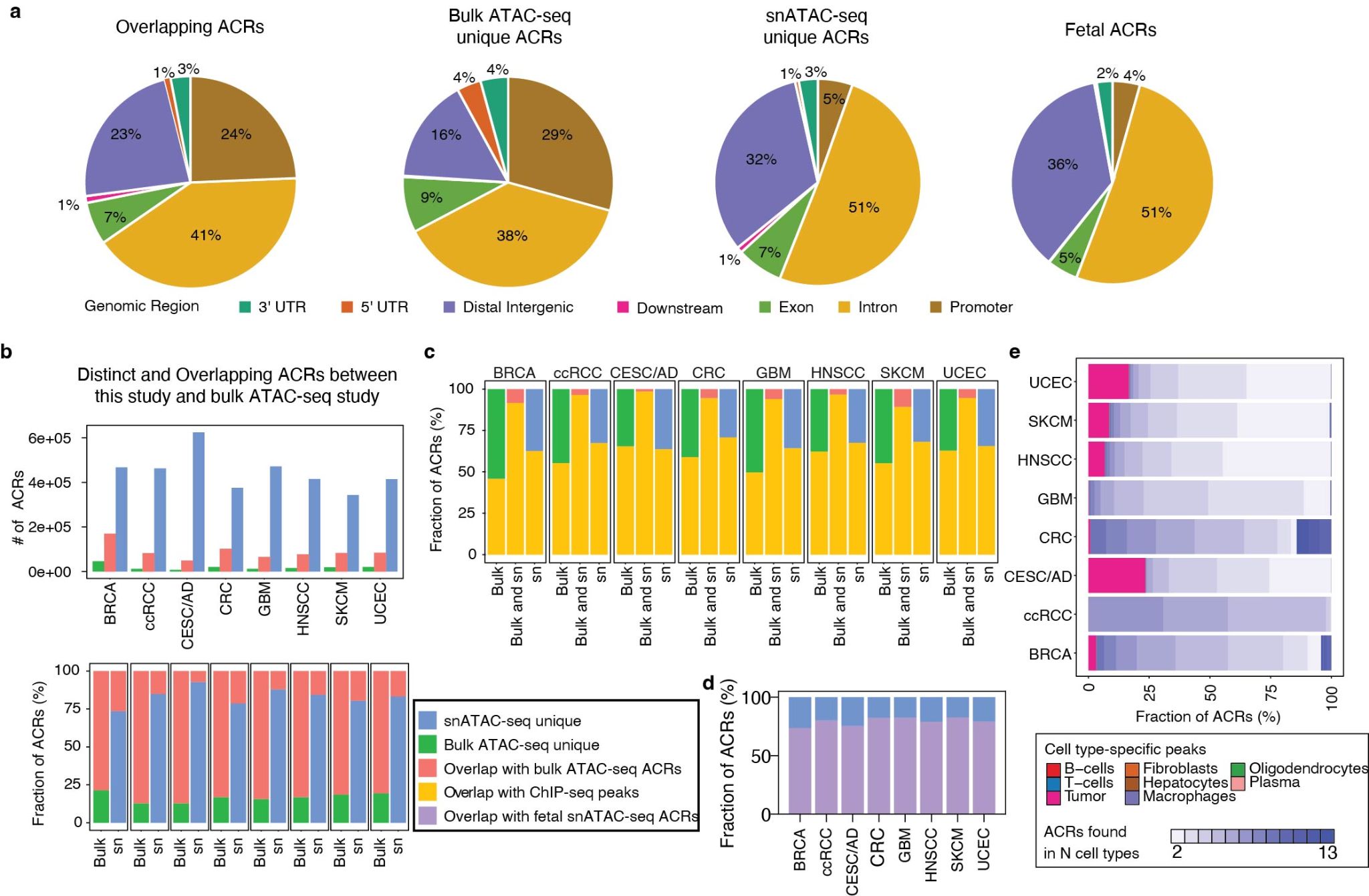
**

## **Supplementary Figure 1. Benchmarking of accessible chromatin regions (ACRs) against public ATAC-seq and ChIP-seq datasets.**

**a**, Distribution of ACRs across genomic regions. ACRs were categorized into four groups: (1) ACRs shared between snATAC-seq (this study) and bulk ATAC-seq[^2^](https://www.zotero.org/google-docs/?I9C0Xr), (2) ACRs unique to the bulk ATAC-seq study, (3) ACRs unique to this study, and (4) fetal ACRs[^1^](https://www.zotero.org/google-docs/?xBGqzT). **b**, Comparison of distinct and overlapping ACRs between this study and the bulk ATAC-seq study[^2^](https://www.zotero.org/google-docs/?yKQc49). The number and the fraction of ACRs are shown in the upper and lower panels, respectively. **c**, Bar plot showing overlap of distinct and shared ACRs between this study and bulk ATAC-seq study[^2^](https://www.zotero.org/google-docs/?IOuu4i) with ENCODE ChIP-seq peaks. Sn: this study; bulk: the bulk ATAC-seq study. **d**, Bar plot showing overlap of snATAC-seq unique ACRs and the fetal snATAC-seq ACRs[^1^](https://www.zotero.org/google-docs/?Se6XN1). **e**, Bar plot showing fractions of snATAC-seq unique ACRs found in one or multiple cell types of tumor microenvironment.

#

# **Supplementary Note 2. Cell type annotation using sc/sn-RNA-seq**

The combined sc/snRNA-seq data yielded 1,157,955 cells/nuclei, which were annotated by the expression of curated epithelial, immune, and stromal marker genes (**Supplementary Figure 2c-f**, and **Supplementary Table 1e**). sc/snRNA-seq cell annotation was further used to annotate the snATAC-seq dataset and show strong agreements between the cell fractions (see Methods and **Supplementary Figure 2a**). On average, 55% of cells in each tumor sample were cancer cells. Annotation of cancer cells was confirmed by the copy-number status of cells from InferCNV analysis (**Supplementary Figure 2b)**, and sc/snRNA-seq and snATAC-seq paired samples yielded highly similar cell type content estimates (**Supplementary Figure 2a**). This suggests cell identities benchmarked by orthogonal methods are consistent.

**
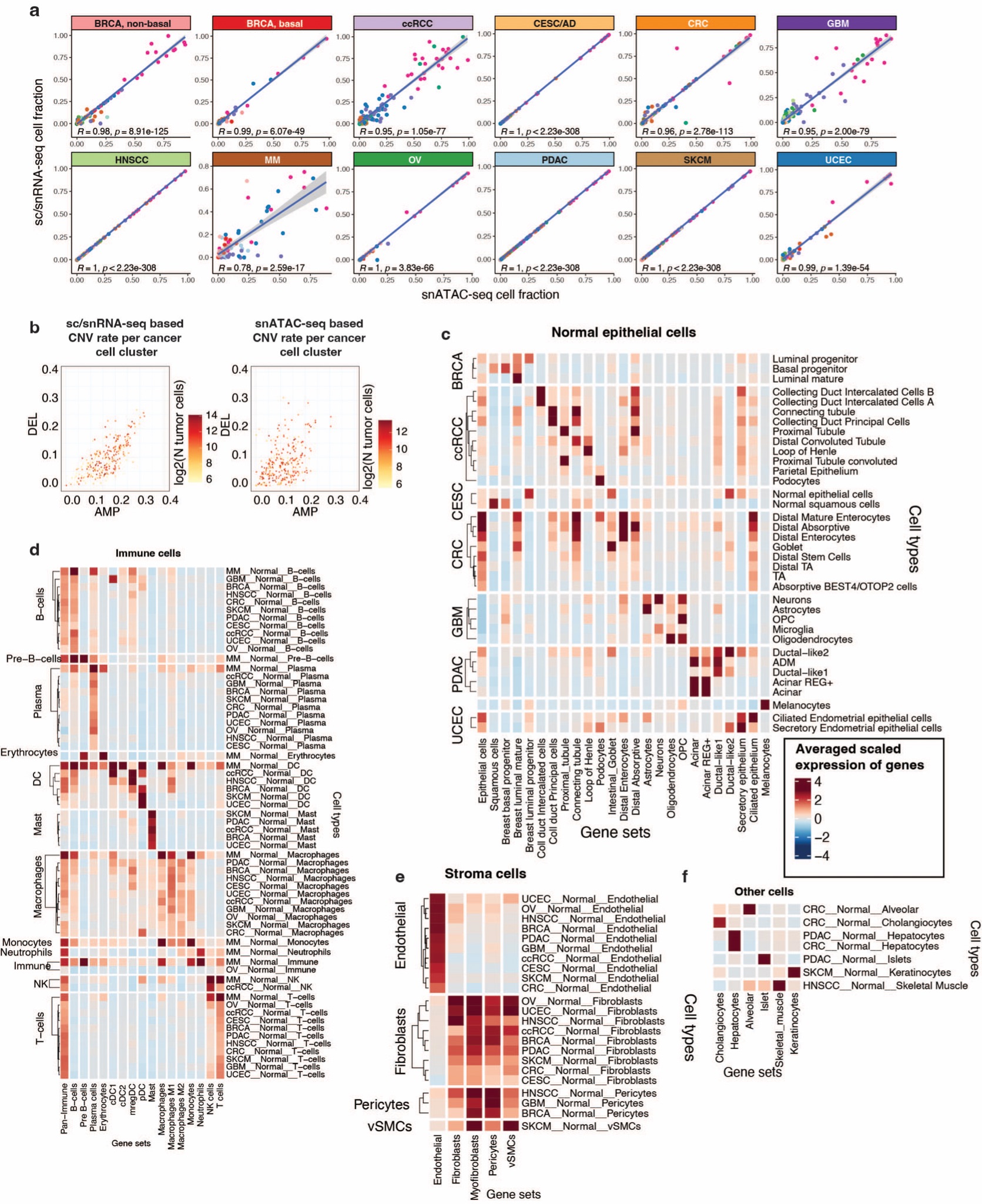
**

## **Supplementary Figure 2. Cell type annotation using sc/sn-RNA-seq**.

**a**, Correspondence between sc/snRNA- and snATAC-seq cell type annotations. Each dot corresponds to cell type fractions in snATAC-seq and sc/snRNA-seq of matching samples, and it is color-coded by cell type (see the legend of **Extended Data Fig. 1b**). The gray band corresponds to the 95% confidence level interval for predictions from the linear model. Pearson R correlation coefficients and t-test two-sided *p*-values are shown. **b**. Scatter plots showing fraction of tumor cells affected by CNV (amplification or deletion) in each cancer cell cluster of the sc/snRNA-seq dataset (left) and snATAC-seq dataset (right). **c-f**, Heatmaps of averaged expression of marker gene sets (x-axis, **Supplementary Table 1e**) in normal epithelial and brain cell types (**c**), immune cell types (**d**), stromal cell types (**e**), and all other cell types (**f**) in each cancer type (y-axis) based on sc/snRNA-seq dataset. Normalized expression of marker genes was averaged across single cells of each cell type, scaled, and then expression of all genes within each marker gene set was further averaged.

# **Supplementary Note 3. Tissue- and cancer cell-specific DACRs validation using published snATAC-seq dataset**

To validate tissue- and cancer cell-specific DACRs, we overlapped them with cell type-specific regions from a previous snATAC-seq study on adult and fetal chromatin accessibility[^5^](https://www.zotero.org/google-docs/?sCi5J3). As anticipated, the DACRs showed significant overlap with cell type-specific regions (**Supplementary Figure 3**).

**
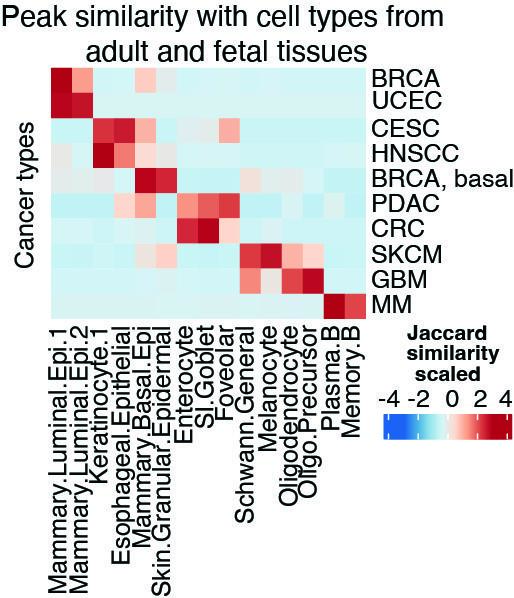
**

## **Supplementary Figure 3. DACRs validation using published snATAC-seq dataset**.

Overlap of cell type annotation between this study and the adult and fetal chromatin accessibility study[^5^](https://www.zotero.org/google-docs/?rSv0BN).

#

# **Supplementary Note 4. Regulon validation using our and published datasets**

To further validate the regulons we prioritized in **Fig. 3a**, we used several approaches. First, we performed differentially accessible transcription factor (TF) motif analysis between cancer cells and respective closest normal cells (CNCs) and confirmed 28 out of 46 (61%) cancer cell-associated regulons (**Supplementary Table 4c, e**). Next, we observed that regulons within the same cancer type had higher similarity in terms of gene targets with each other compared to regulons from other cancer types (**Supplementary Figure 4a**), supporting the existence of cancer-specific TF networks. We also looked into public databases and found that 41 out of 87 tissue and cancer cell-specific TFs were also differentially expressed in previous sc/snATAC-seq or bulk ATAC-seq studies, including BRCA[^6^](https://www.zotero.org/google-docs/?RLvzOe), MM[^7^](https://www.zotero.org/google-docs/?F7FEfZ), ccRCC[^8^](https://www.zotero.org/google-docs/?oryKX1), PDAC[^9^](https://www.zotero.org/google-docs/?FNZCr9), pan-organ chromatin accessibility[^5^](https://www.zotero.org/google-docs/?fBkS92), and the bulk ATAC-seq study in human cancers[^2^](https://www.zotero.org/google-docs/?xAdfAl) (**Supplementary Table 5a**).

Literature supports our regulon analysis. We identified KLF6 as a potential mediator of normal-to-cancer transcriptional changes in both PDAC and ccRCC. KLF6 has been identified as an important regulator of metabolic changes, potentially acting downstream of KRAS and MEK to promote polyamine synthesis to drive PDAC cell growth and survival[^10^](https://www.zotero.org/google-docs/?sWAePC). PITX3 transcription factor binding sites (TFBS) are overexpressed in high grade GBM[^11^](https://www.zotero.org/google-docs/?Qsya0E) and upregulation of the ZNF148/PTX3 axis promotes malignant transformation in GBM[^12^](https://www.zotero.org/google-docs/?C3uK8F).

Motif overrepresentation analysis can be another complementary approach to validate the regulons. We implemented motif overrepresentation analysis in DACRs between cancer cells from primary tumors vs their CNCs, and between cancer cells from metastatic tumors vs cancer cells from primary tumors. We examined the top 20 motifs enriched in DACRs more accessible in primary cancer cells (in red) or DACRs more accessible in normal cells (in blue) in each cancer type, grouped by TF family and annotated by concordance with ChromVar results (**Supplementary Figure 4b**). A similar analysis shows TF motifs enriched in DACRs found between metastatic and primary cancer cells (**Supplementary Figure 4c**). Most of the motifs found by overrepresentation analysis were also identified with ChromVar analysis. The most prominent examples include basic leucine zipper factors (FOS, JUN, BATF), fork head factors (FOX, RFX), nuclear receptors with C4 zinc fingers (NR3C1, HNF4A), and tryptophan cluster factors (ETV, ETS). We also validated significant cancer cell-specific regulons. FOXL1 and MAFK motifs were enriched in DACRs upregulated in primary PDAC compared to its CNCs (**Supplementary Figure 4b**).

# **
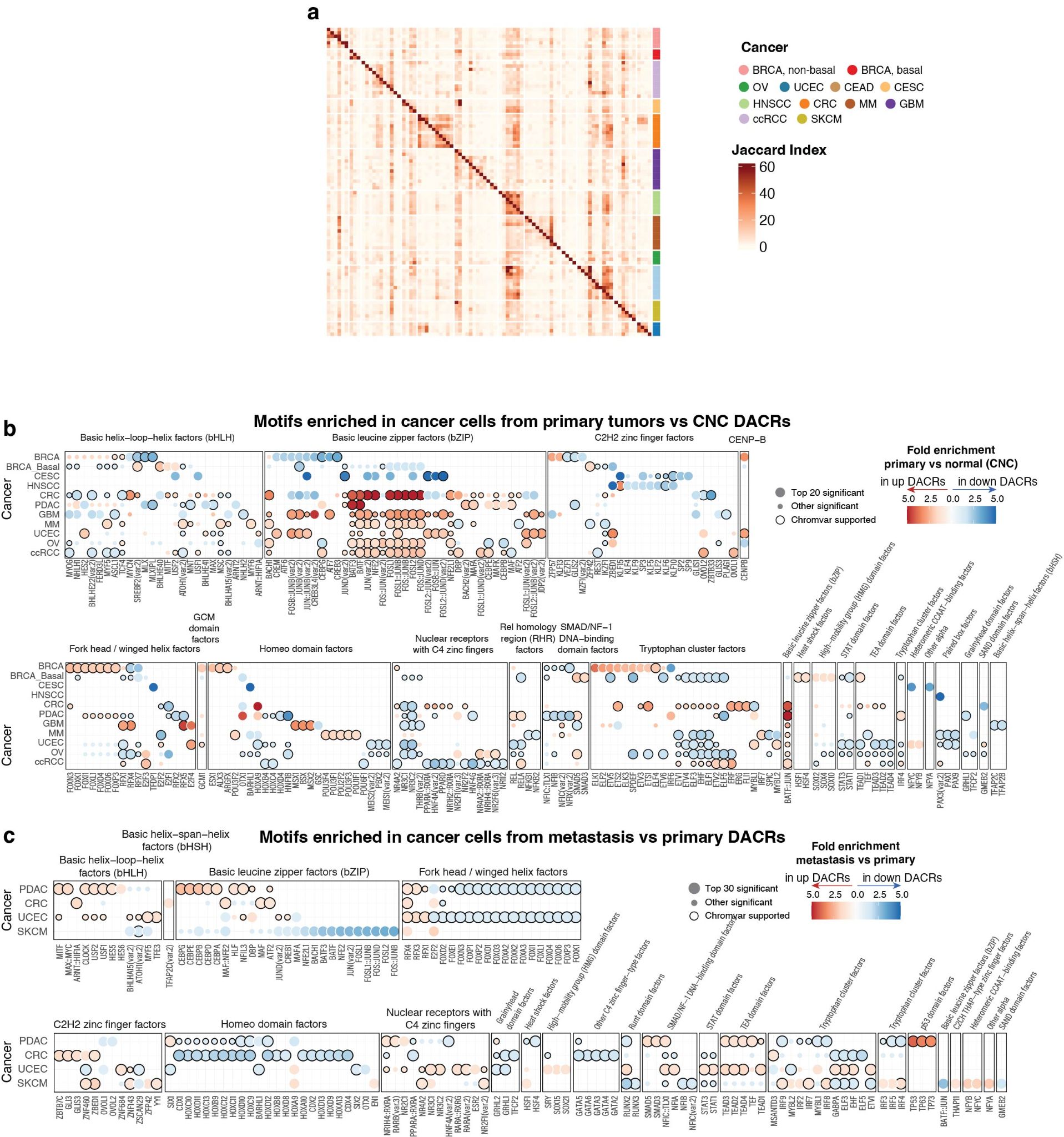
**

## **Supplementary Figure 4. Tissue- and cancer-specific regulons and TFs.**

**a,** Heatmap showing pairwise similarity (i.e Jaccard index) of tissue and cancer cell-specific regulons’ target genes. **b-c,** Bubble plots representing the top 20 motifs enriched in upregulated and downregulated DACRs for each cancer type for the comparisons: (**b**) cancer cells from primary tumors vs CNC and for (**c**) cancer cells from primary tumors vs cancer cells from metastatic tumors. The motifs are grouped by the TF family and annotated by their concordance with ChromVar scores. The red bubbles indicate motifs enriched in up-regulated DACRs, and the blue bubbles indicate those enriched in down-regulated DACRs.

#

# **Supplementary Note 5. TFs associated with pseudotime in 9 cases with paired primary tumor and metastasis samples**

We correlated trajectories’ pseudotimes from all samples with TF motifs scores obtained from snATAC-seq (**Supplementary Table 7**). The CRC samples pseudotimes were all correlated with TCF7 and LEF1 (**Supplementary Table 7**), which are known to drive Wnt-signaling, a pathway associated with EMT in CRC[^13^](https://www.zotero.org/google-docs/?hGoFCA). The UCEC samples were much more heterogeneous in terms of correlation of TF motifs with pseudotime. For example, trajectories of two cases, CPT4427DU and CPT704DU, were positively correlated with TEAD1 and TEAD2, which have been implicated in cell migration and invasion in endometrial carcinomas[^14^](https://www.zotero.org/google-docs/?UWhYc9), whereas the trajectories of other two cases, CPT1541DU and CPT4096DU, were both positively correlated with NFKB1 and TBX3, with the latter being associated with metastasis in some cancers[^15,16^](https://www.zotero.org/google-docs/?WNOS5q).

#

# **Supplementary Note 6. Pathway enrichment in ACRs associated with TFs involved in metastasis across 9 cases with paired primary tumor and metastasis samples**

We further sought to identify pathways associated with TFs involved in metastasis (**Supplementary Table 6d**). We first associated ACR accessibility with TF scores and then performed pathway enrichment analysis on significantly associated ACRs (see Methods). Our analysis revealed that ACRs associated with FOX-family TFs, including FOXP1 and FOXA1, were significantly more enriched in metastasis-associated pathways compared to other TF-associated ACRs in both CRC and UCEC (**Supplementary Figure 5**). Moreover, we observed that FOXP1 and FOXA1 were negatively correlated with pseudotime in a majority of samples for both UCEC and CRC (**Supplementary Table 7**) suggesting that FOXP1 and FOXA1 are negative regulators for metastasis in these cancer types.

# **
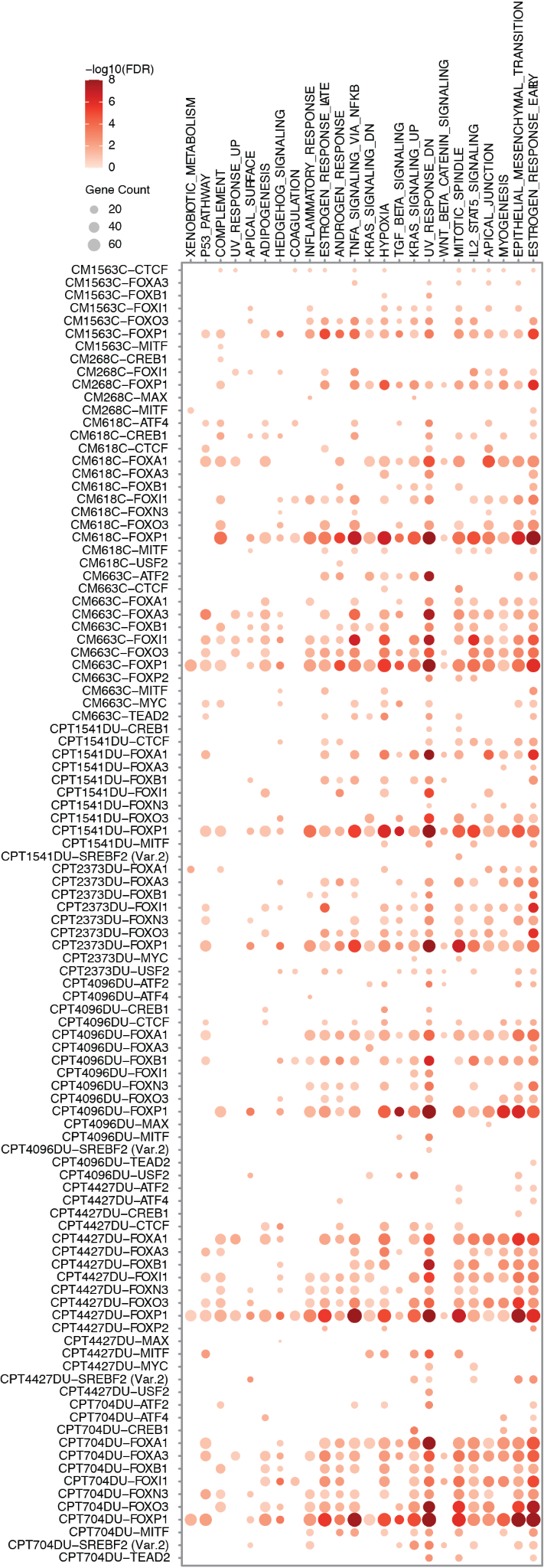
**

#

## **Supplementary Figure 5. Pathway enrichment analysis in DACRs associated with TFs involved in metastasis.**

Bubble plot showing pathways enriched in genes nearest to DACRs (metastasis vs primary) significantly associated with the TFs (see Methods) that were also associated with pseudotime from individual cases.

# **Supplementary Note 7. Characterization of mutational landscape in 11 cancers**

In this pan-cancer cohort, four main mutational signatures were detected by non-negative matrix factorization (NMF), namely APOBEC cytidine deaminase, spontaneous deamination of 5-methylcytosine, exposure to tobacco mutagens, and UV exposure (**Supplementary Figure 6a-b**). Furthermore, *TP53, KRAS, VHL, KMT2D, PIK3CA, PBRM1, KMT2C, NF1, APC*, and *ARID1A* were identified as the top 10 frequently mutated genes. They can be divided into two categories: cancer-type-specific, such as *VHL* in ccRCC and *APC* in CRC, and pan-cancer, for instance, *TP53, KRAS, KMT2D, PIK3CA*, and *KMT2C* (**Supplementary Figure 6c** and **Supplementary Table 8**). As for CNVs, the top frequent events included chr8q, chr7p, and chr3p (**Extended Data Fig. 10a**)

# **
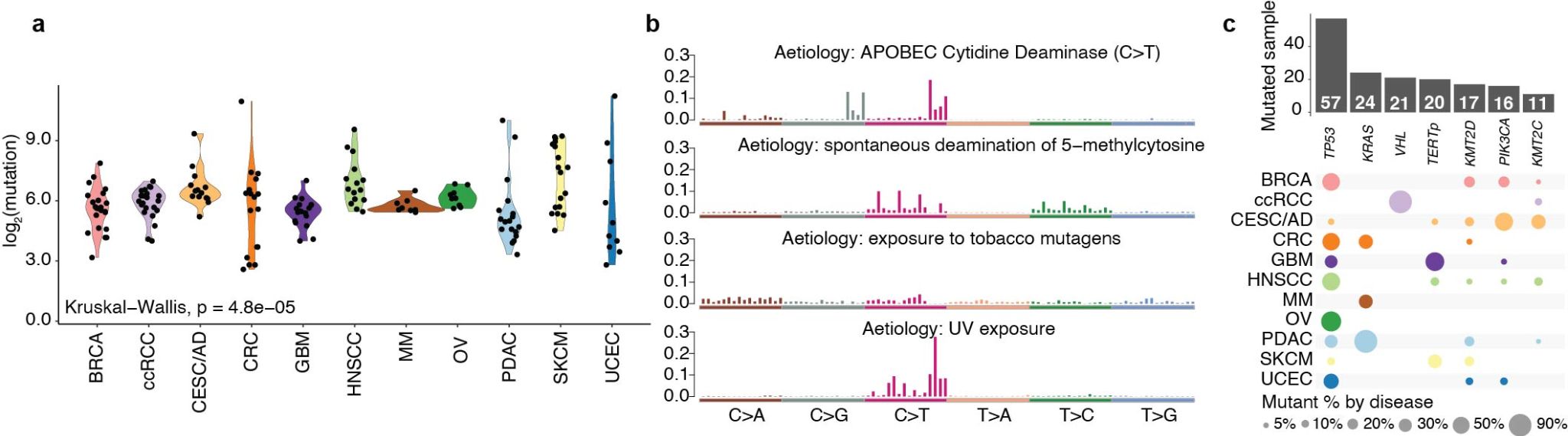
**

## **Supplementary Figure 6. Mutational landscape of 11 cancer types.**

**a**, The distribution of WES-based somatic mutation counts (i.e., log2-scale) across 11 cancer types with corresponding sample sizes: BRCA (*n*=23), ccRCC (*n*=23), CESC/AD (*n*=14), CRC (*n*=17), GBM (*n*=18), HNSCC (*n*=15), MM (*n*=8), OV (*n*=9), PDAC (*n*=18), SKCM (*n*=18), and UCEC (*n*=10). The Kruskal-Wallis H test *p*-value is shown. **b**, The WES-based mutational signatures identified in this pan-cancer cohort. **c**, Upset plot showing the representative frequently mutated genes identified in our dataset.

# **Supplementary Note 8. Limitation of the study**

Although our study provides important insights into potential epigenetic drivers of cancer, it is important to acknowledge some limitations. First, we have a cohort size of 225 samples across cancer types but just a little over 20 samples on average per cancer type. Future studies could benefit from a larger cohort to expand our knowledge of tumor heterogeneity. Second, further functional validation is needed to confirm the clinical relevance of our findings. Despite these limitations, our study is a valuable resource for the research community, providing a better understanding of epigenetic regulations during oncogenesis and supporting the development of novel therapeutic approaches for cancer treatment.


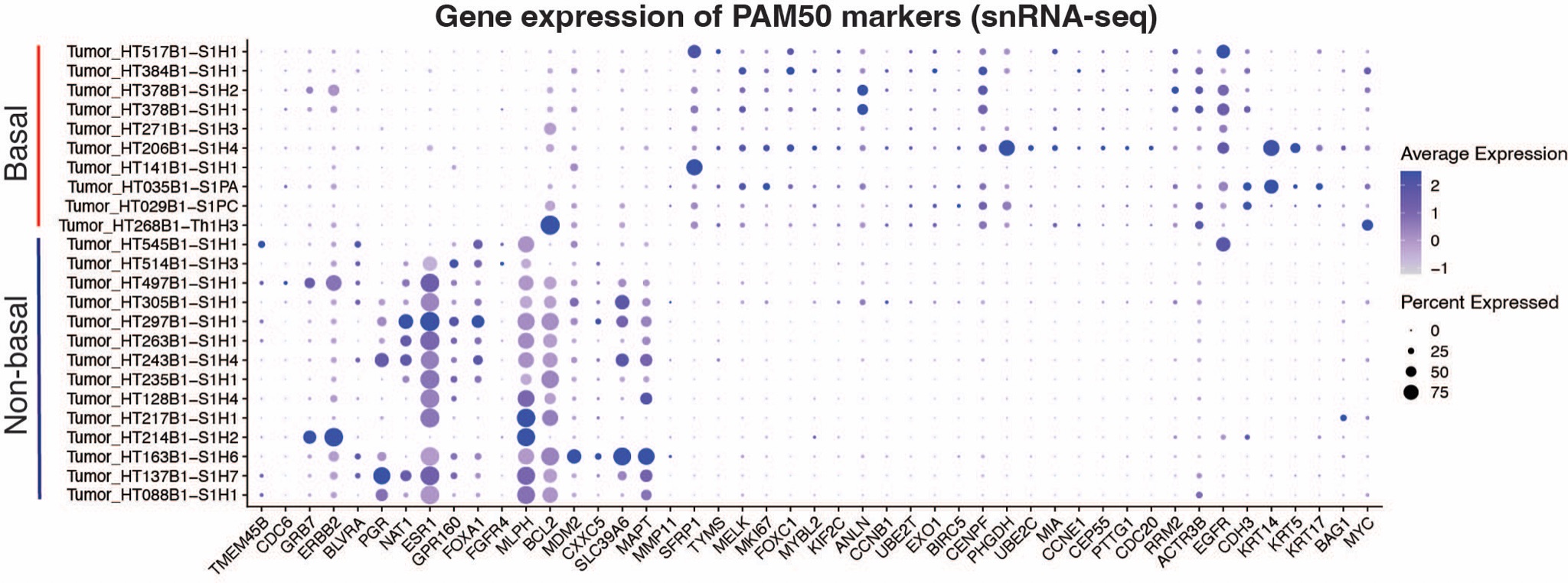


## **Supplementary Figure 7. Basal and non-basal subtype annotation using snRNA-seq data.**

Dot plot showing gene expression of PAM50 markers in cancer cells of snRNA-seq samples. Markers on the left distinguish non-basal samples, while markers on the right are specific to basal samples. Expression is shown for all PAM50 markers that were quantified in the snRNA-seq dataset.


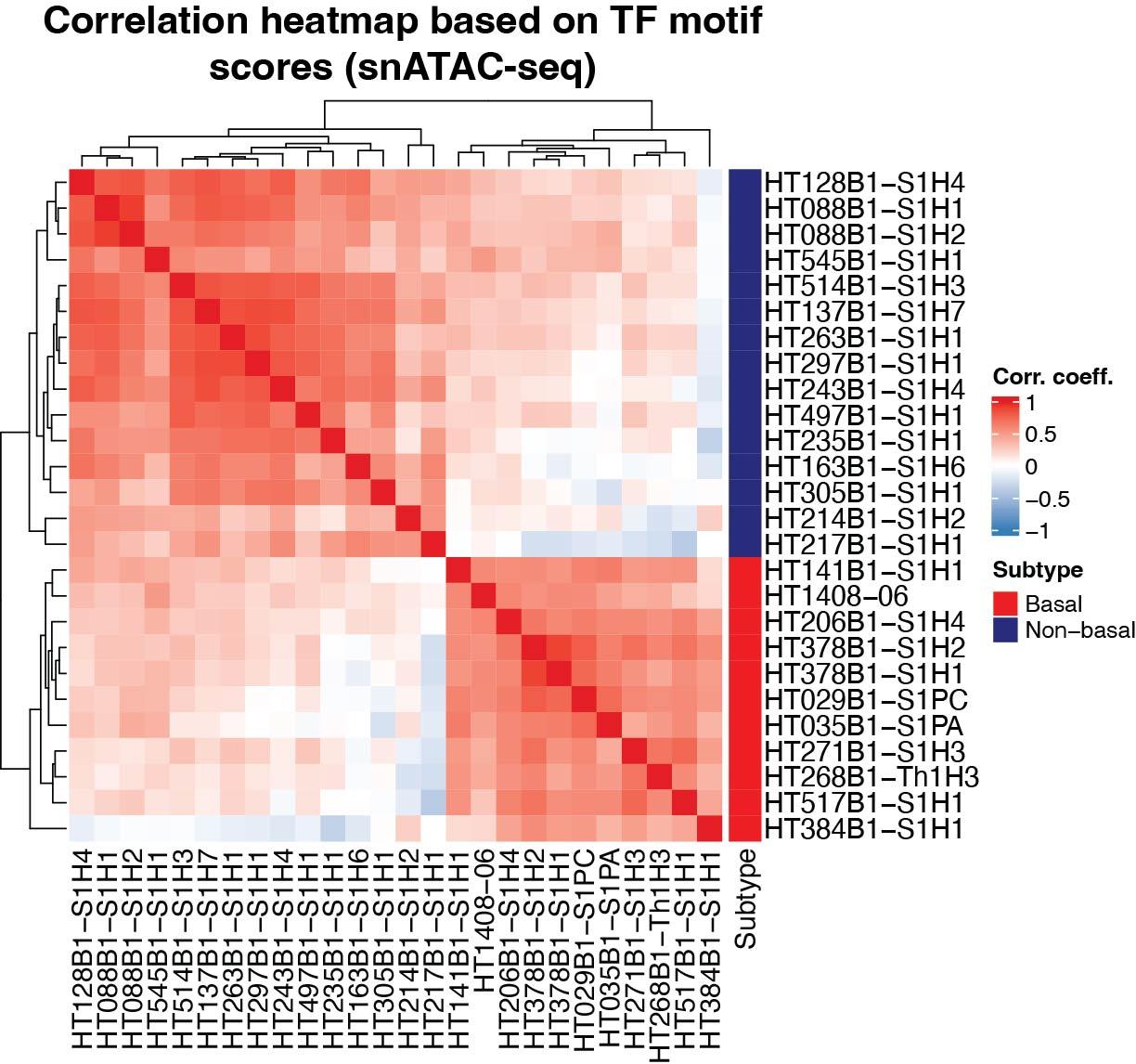


## **Supplementary Figure 8. Basal and non-basal subtype annotation using snATAC-seq data.**

Heatmap showing Spearman’s rank correlation coefficients between snATAC-seq samples (cancer cells only) based on TF motif scores. For each TF, scores were averaged across all cancer cells of each sample, and then pairwise correlation was calculated using all TF scores from the dataset. We observe two distinct clusters of samples corresponding to samples of basal and non-basal subtypes.

#

# **Captions for Supplementary Tables 1 to 9**

## **Supplementary Table 1: Dataset overview.**

**a,** Samples used in the study. **b,** Clinical and demographic information for samples used in the study. **c,** Number of peaks between this study and bulk ATAC-seq study[^2^](https://www.zotero.org/google-docs/?BlrPab). **d,** Number of peaks in different genomic regions between this study and bulk ATAC-seq study[^2^](https://www.zotero.org/google-docs/?Szy2K6). **e,** Marker genes for cell type annotation.

## **Supplementary Table 2: Tissue- and cancer cell-specific DEGs/DACRs identified in this study.**

**a,** Tissue- and cancer cell-specific DACRs. Statistical analysis was performed using a logistic regression test with fraction of fragments in peaks used as a latent variable, and Bonferroni correction was applied for the resulting two-sided *p*-values. **b,** Tissue- and cancer cell-specific DEGs. Statistical analysis was performed using a two-sided Wilcoxon rank-sum test, and Bonferroni correction was applied for the resulting *p*-values. **c,** Tissue- and cancer cell-specific DACRs, top by Fch (shown in the **Extended Data Fig. 2g**). **d,** Identification of CNC. Pearson R correlation coefficients are shown for cancer cells from each tumor and normal cell type pair using aggregated per sample (for cancer cells) or per cohort (for normal cells) chromatin accessibilities. **e,** Cancer cell-specific DACRs. Statistical analysis was performed using a logistic regression test with fraction of fragments in peaks used as a latent variable, and Bonferroni correction was applied for the resulting two-sided *p*-values. **f,** Cancer cell-specific DEGs. Statistical analysis was performed using a two-sided Wilcoxon rank-sum test, and Bonferroni correction was applied for the resulting *p*-values.

## **Supplementary Table 3: ACR-to-gene links in PDAC and BRCA cohorts connecting a DACR and a DEG.**

**a,** Explanation of column names. **b,** Links included in **Fig. 2d**. **c,** Links included in **Extended Data Fig 5g**. In **b-c**, the *p*-value is computed as the probability of observing a greater correlation coefficient given the distribution of correlation coefficients from a set of background peaks.

## **Supplementary Table 4: Tissue- and cancer-cell specific regulons and TFs identified in this study.**

**a,** The table contains gene target lists for 258 SCENIC regulons. **b,** Differential regulons between cancer cells from each tumor type vs cancer cells from all other tumor types. This analysis was done using only primary tumor samples. **c,** Differential regulons between cancer cells from each tumor type (primary tumor samples only) vs its CNC. **d,** Differentially accessible TF motifs (DAMs) between primary cancer cells from one tumor type and pooled cancer cells from all other primary tumors. **e,** DAMs between primary cancer cells and their respective CNC. For all statistical analyses in **Supplementary Table 4b-e**, two-sided Wilcoxon rank-sum test was performed, and Benjamini-Hochberg FDR correction was applied for the resulting *p*-values. **f,** Prioritized regulons shown in **Fig. 3a**.

## **Supplementary Table 5: Confirming tissue- and cancer-cell specific TFs using published datasets.**

**a,** Identification of tissue- or cancer cell-specific TFs that are differentially expressed in published datasets. **b**, Metadata for ENCODE ChIP-seq biosamples. **c**, Identification of the direct binding of TFs to the promoters of their target genes using ENCODE ChIP-seq.

## **Supplementary Table 6: Identifying DACRs, DEGs, TFs, and regulons associated with metastasis.**

**a,** DACRs obtained by comparing metastatic cancer cells vs primary cancer cells from each tumor type using snATAC-seq data. Statistical analysis was performed using a logistic regression test with fraction of fragments in peaks used as a latent variable, and Bonferroni correction was applied for the resulting two-sided *p*-values. **b,** DEGs obtained by comparing metastatic cancer cells vs primary cancer cells from each tumor type using snRNA-seq data. Statistical analysis was performed using a two-sided Wilcoxon rank-sum test, and Bonferroni correction was applied for the resulting *p*-values. **c,** Prioritized metastasis associated DACRs that are shown in **Extended Data Fig. 8a**. Peaks were annotated using the ChIPseeker package (see Methods). **d,** DAMs obtained by comparing metastatic cancer cells vs primary cancer cells from each tumor type using snATAC-seq data. Statistical analysis was performed using a two-sided Wilcoxon rank-sum test, and Benjamini-Hochberg FDR correction was applied for the resulting *p*-values. **e,** Differential regulons obtained by comparing metastatic cancer cells vs primary cancer cells from each tumor type. Statistical analysis was performed using a two-sided Wilcoxon rank-sum test, and FDR correction was applied for the resulting *p*-values. **f,** Prioritized metastasis associated TFs that are shown in **Fig. 4a**.

## **Supplementary Table 7: Correlation of TF motif scores with pseudotime.**

Pearson correlation coefficient between each TF motif score and pseudotime found by Slingshot for 9 cases with paired primary/metastatic snATAC-seq samples. The *p*-values were obtained from the two-sided t-test, and then were adjusted by Benjamini-Hochberg correction.

## **Supplementary Table 8: Impact of genetic drivers.**

**a,** The cancer driver gene list. **b,** The driver mutation frequency in this pan-cancer cohort. **c,** The summary driver mutation metatable. **d,** The driver mutation status. **e,** CNV arm-level calls. **f,** *KRAS* mutation mapping calls based on sn and bulk data. **g,** The bulk tumor look-up table of this pan-cancer cohort. **h,** Driver mutation VAF summary. **i,** Sample-level mutation count. **j**, *EGFR* CNV calls per sample.

## **Supplementary Table 9: Regulons associations with clinical features.**

**a,** Clinical information and regulon scores for TCGA samples. **b,** The impact of regulon activity on overall survival and progression free survival in TCGA-GBM cohort. **c,** The impact of regulon activity on overall survival and progression free survival in TCGA-PDAC cohort. **d,** The impact of regulon activity on overall survival and progression free survival in TCGA-CRC cohort. **e,** Association between HPV infection and regulon activity in HNSCC (this study and TCGA cohort). The Wilcoxon rank-sum test two-sided FDR adjusted *p-*values are shown.

# References

[1. Domcke Silvia *et al.* A human cell atlas of fetal chromatin accessibility. *Science* **370**, eaba7612 (2020).](https://www.zotero.org/google-docs/?GNHtoT)

[2. Corces M. Ryan *et al.* The chromatin accessibility landscape of primary human cancers. *Science* **362**, eaav1898 (2018).](https://www.zotero.org/google-docs/?GNHtoT)

[3. Dunham, I. *et al.* An integrated encyclopedia of DNA elements in the human genome. *Nature* **489**, 57–74 (2012).](https://www.zotero.org/google-docs/?GNHtoT)

[4. Luo, Y. *et al.* New developments on the Encyclopedia of DNA Elements (ENCODE) data portal. *Nucleic Acids Res.* **48**, D882–D889 (2020).](https://www.zotero.org/google-docs/?GNHtoT)

[5. Zhang, K. *et al.* A single-cell atlas of chromatin accessibility in the human genome. *Cell* **184**, 5985-6001.e19 (2021).](https://www.zotero.org/google-docs/?GNHtoT)

[6. Kumegawa, K. *et al.* GRHL2 motif is associated with intratumor heterogeneity of cis-regulatory elements in luminal breast cancer. *Npj Breast Cancer* **8**, 70 (2022).](https://www.zotero.org/google-docs/?GNHtoT)

[7. Frede, J. *et al.* Dynamic transcriptional reprogramming leads to immunotherapeutic vulnerabilities in myeloma. *Nat. Cell Biol.* **23**, 1199–1211 (2021).](https://www.zotero.org/google-docs/?GNHtoT)

[8. Long, Z. *et al.* Single-cell multiomics analysis reveals regulatory programs in clear cell renal cell carcinoma. *Cell Discov.* **8**, 68 (2022).](https://www.zotero.org/google-docs/?GNHtoT)

[9. Fan, X. *et al.* Integrated single-cell multiomics analysis reveals novel candidate markers for prognosis in human pancreatic ductal adenocarcinoma. *Cell Discov.* **8**, 13 (2022).](https://www.zotero.org/google-docs/?GNHtoT)

[10. Lee, M.-S. *et al.* Ornithine aminotransferase supports polyamine synthesis in pancreatic cancer. *Nature* **616**, 339–347 (2023).](https://www.zotero.org/google-docs/?GNHtoT)

[11. Roura, A.-J. *et al.* Regulatory networks driving expression of genes critical for glioblastoma are controlled by the transcription factor c-Jun and the pre-existing epigenetic modifications. *Clin. Epigenetics* **15**, 29 (2023).](https://www.zotero.org/google-docs/?GNHtoT)

[12. Cheng, S. *et al.* Upregulation of the ZNF148/PTX3 axis promotes malignant transformation of dendritic cells in glioma stem-like cells microenvironment. *CNS Neurosci. Ther.* **n/a**, (2023).](https://www.zotero.org/google-docs/?GNHtoT)

[13. Mayer, C.-D., Magon de La Giclais, S., Alsehly, F. & Hoppler, S. Diverse LEF/TCF Expression in Human Colorectal Cancer Correlates with Altered Wnt-Regulated Transcriptome in a Meta-Analysis of Patient Biopsies. *Genes* **11**, (2020).](https://www.zotero.org/google-docs/?GNHtoT)

[14. Liu, X. *et al.* Tead and AP1 Coordinate Transcription and Motility. *Cell Rep.* **14**, 1169–1180 (2016).](https://www.zotero.org/google-docs/?GNHtoT)

[15. Dong, L. *et al.* Novel HDAC5-interacting motifs of Tbx3 are essential for the suppression of E-cadherin expression and for the promotion of metastasis in hepatocellular carcinoma. *Signal Transduct. Target. Ther.* **3**, 22 (2018).](https://www.zotero.org/google-docs/?GNHtoT)

[16. Zimmerli, D. *et al.* TBX3 acts as tissue-specific component of the Wnt/β-catenin transcriptional complex. *eLife* **9**, e58123 (2020).](https://www.zotero.org/google-docs/?GNHtoT)
