# Supplementary material for: Epigenetic regulation during cancer transitions across 11 tumour types
Source: Nature. 2023 Nov 1;623(7986):432–41. doi: 10.1038/s41586-023-06682-5 (PMC10632147; doi:10.1038/s41586-023-06682-5)
Supplement: Supplementary file 2 — Reporting Summary [file 41586_2023_6682_MOESM2_ESM.pdf]

nature portfolio

Corresponding author(s): Li Ding, Benjamin J. Raphael, Feng Chen, Ryan C. Fields

Last updated by author(s): Aug 31, 2023

# Reporting Summary

Nature Portfolio wishes to improve the reproducibility of the work that we publish. This form provides structure for consistency and transparency in reporting. For further information on Nature Portfolio policies, see our Editorial Policies and the Editorial Policy Checklist.

## Statistics

For all statistical analyses, confirm that the following items are present in the figure legend, table legend, main text, or Methods section.

| n/a | Confirmed | |
|---|---|---|
| ☐ | ☒ | The exact sample size (*n*) for each experimental group/condition, given as a discrete number and unit of measurement |
| ☐ | ☒ | A statement on whether measurements were taken from distinct samples or whether the same sample was measured repeatedly |
| ☐ | ☒ | The statistical test(s) used AND whether they are one- or two-sided<br>*Only common tests should be described solely by name; describe more complex techniques in the Methods section.* |
| ☐ | ☒ | A description of all covariates tested |
| ☐ | ☒ | A description of any assumptions or corrections, such as tests of normality and adjustment for multiple comparisons |
| ☐ | ☒ | A full description of the statistical parameters including central tendency (e.g. means) or other basic estimates (e.g. regression coefficient) AND variation (e.g. standard deviation) or associated estimates of uncertainty (e.g. confidence intervals) |
| ☐ | ☒ | For null hypothesis testing, the test statistic (e.g. *F*, *t*, *r*) with confidence intervals, effect sizes, degrees of freedom and *P* value noted<br>*Give P values as exact values whenever suitable.* |
| ☒ | ☐ | For Bayesian analysis, information on the choice of priors and Markov chain Monte Carlo settings |
| ☒ | ☐ | For hierarchical and complex designs, identification of the appropriate level for tests and full reporting of outcomes |
| ☐ | ☒ | Estimates of effect sizes (e.g. Cohen's *d*, Pearson's *r*), indicating how they were calculated |

*Our web collection on statistics for biologists contains articles on many of the points above.*

## Software and code

Policy information about availability of computer code

| | |
|---|---|
| Data collection | No software was used for data collection |
| Data analysis | 10Xmapping, https://github.com/ding-lab/10Xmapping<br>AUCell v1.19.1, R-package<br>BWA v0.7.17, https://github.com/lh3/bwa<br>Cell Ranger v6.0.2, 10X Genomics<br>Cell Ranger ATAC v2.0, 10X Genomics<br>Cell Ranger ARC v2.0, 10X Genomics<br>ChIPseeker v1.26.2, R-package<br>chromVAR v1.12.0, R-package<br>COCOON, https://github.com/ding-lab/COCOONS<br>ComplexHeatmap v2.14.0, R-package<br>data.table v1.14.6, R-package<br>dplyr v1.0.10, R-package<br>EnsDb.Hsapiens.v86 v2.99.0, R-package<br>fgsea v1.16.0, R-package<br>future v1.21.0, R-package<br>GATK v4.1.2.0 and v4.1.9.0 https://github.com/broadgsa/gatk<br>ggplot2 v3.3.5, R-package<br>InferCNV v0.99.7 and v1.11.2, https://github.com/broadinstitute/infercnv<br>Louvain algorithm (used by FindClusters function of Seurat v.4.0.5, R package) |

MACS2 v2.2.7.1, https://github.com/macs3-project/MACS
matplotlib v3.4.2, py39hf3d152e_0
MuTect v1.1.7, https://github.com/broadinstitute/mutect
MuTect2 https://gatk.broadinstitute.org/hc/en-us/articles/360036733771-Mutect2
motifmatchr v1.12.0, R-package
numpy v1.21.1, h49503c6_1_cpython
org.Hs.eg.db v3.12.0, R-package
pandas v1.3.1, py39hde0f152_0
Picard v2.6.26, https://github.com/broadinstitute/picard
Pindel v0.2.5, https://github.com/genome/pindel
plyr v1.8.8, R-package
pySCENIC v0.11.2, https://pyscenic.readthedocs.io/en/latest/index.html
python v3.7.12 and v.3.9.6
RColorBrewer v1.1.3, R-package
reshape v0.8.9, R-package
reshape2 v1.4.4, R-package
Rtsne v0.16, R-package
samtools v1.14, https://github.com/samtools/samtools
Scrublet v0.2.3, https://github.com/swolock/scrublet
sctransform v0.3.2, R-package
scVarScan, https://github.com/ding-lab/10Xmapping
Seurat v4.0.5, R-package
Signac v1.3.0 and v.1.8.0, R-package
sklearn v0.24.2, python-package
slingshot v2.5.1, R-package
Somaticwrapper v1.6, https://github.com/ding-lab/somaticwrapper
Somaticwrapper tonly.v1.0, https://github.com/ding-lab/somaticwrapper/tree/tonly.v1.0
survival v3.2.7, R-package
survminer v0.4.9, R-package
Strelka v2.9.2, https://github.com/Illumina/strelka
tidyverse v1.3.2, R-package
trimGalore v.0.6.7, https://github.com/FelixKrueger/TrimGalore
TxDb.Hsapiens.UCSC.hg38.knownGene v3.10.0, R-package
VarScan v2.3.8, https://dkoboldt.github.io/varscan
VirusScan, https://github.com/ding-lab/VirusScan/tree/simplified
Custom codes developed in the study are available on GitHub (https://github.com/ding-lab/PanCan_snATAC_publication)

For manuscripts utilizing custom algorithms or software that are central to the research but not yet described in published literature, software must be made available to editors and reviewers. We strongly encourage code deposition in a community repository (e.g. GitHub). See the Nature Portfolio guidelines for submitting code & software for further information.

## Data

Policy information about availability of data

All manuscripts must include a data availability statement. This statement should provide the following information, where applicable:

- Accession codes, unique identifiers, or web links for publicly available datasets
- A description of any restrictions on data availability
- For clinical datasets or third party data, please ensure that the statement adheres to our policy

Sequencing data are part of Human Tumor Atlas Network (HTAN) dbGaP Study Accession: phs002371.v3.p1 (https://www.ncbi.nlm.nih.gov/projects/gap/cgi-bin/study.cgi?study_id=phs002371.v3.p1), and Clinical Proteomic Tumor Analysis Consortium (CPTAC) dbGaP Study Accession: phs001287.v17.p6 (https://www.ncbi.nlm.nih.gov/projects/gap/cgi-bin/study.cgi?study_id=phs001287.v17.p6). Data can be accessed through the HTAN DCC Portal https://data.humantumoratlas.org/ under the HTAN WUSTL Atlas. Sequencing data for CPTAC ccRCC and GBM samples are available through the NCI Genomic Data Commons (GDC) under the CPTAC3 project. Matrices for CPTAC GBM and ccRCC samples and CUT&RUN data are available from the Gene Expression Omnibus (GEO) under respective accession numbers GSE240822 and GSE240699. GRCh38 references used for sc/snRNA-seq (refdata-gex-GRCh38-2020-A) and snATAC-seq and snMultiome-seq (refdata-cellranger-arc-GRCh38-2020-A-2.0.0) analyses are freely available from 10X Genomics website (https://support.10xgenomics.com). The reference GRCh38 genome (GRCh38.d1.vd1.fa.tar.gz) used for WES and CUT&RUN reads alignment is available from GDC (https://gdc.cancer.gov/about-data/gdc-data-processing/gdc-reference-files).

## Research involving human participants, their data, or biological material

Policy information about studies with human participants or human data. See also policy information about sex, gender (identity/presentation), and sexual orientation and race, ethnicity and racism.

| | |
|---|---|
| Reporting on sex and gender | For all samples for MM, OV, BRCA, PDAC, UCEC, CRC, CESC/AD, SKCM, HNSCC, as well as 2 normal adjacent tissues (NATs) for GBM and 1 NAT for ccRCC , samples sex was collected from electronic health record system. For GBM and ccRCC samples originating from the NCI Clinical Proteomic Tumor Analysis Consortium (CPTAC), sex information was obtained from the Protein Data Commons (https://pdc.cancer.gov/pdc). |
| Reporting on race, ethnicity, or other socially relevant groupings | Self-reported race and/or ethnicity information is provided in the Supplementary Table 1b for BRCA, ccRCC, CRC, GBM, MM, and PDAC samples. For BRCA, CRC, MM, and PDAC samples race and/or ethnicity was collected from electronic health record system. For GBM and ccRCC samples originating from the NCI CPTAC, race and ethnicity was obtained from the Protein Data Commons (https://pdc.cancer.gov/pdc). |

| | |
|---|---|
| Population characteristics | Our dataset comprises samples from 11 tumor types, with patients ages 24-88. Distribution of the samples across the cohorts can be found in the Supplementary Table 1a and Extended Data Fig. 1a. Detailed clinical information can be found in the Supplementary Table 1b. |
| Recruitment | Patients who fit the clinical criteria and consented to the study were selected for inclusion in the genetic and molecular tumor analysis. GBM and ccRCC samples originating from the NCI CPTAC were part of the previous studies (Clark et al. 2019 and Wang et al. 2021). There was no self-selection bias or other biases in the recruitment of patients. |
| Ethics oversight | All samples for MM, OV, BRCA, PDAC, UCEC, CRC, CESC/AD, SKCM, HNSCC, as well as 2 NATs for GBM and 1 NAT for ccRCC were collected with informed consent in concordance with Institutional Review Board (IRB) approval at the School of Medicine at Washington University in St Louis. IRB protocols are the following: 201105374, 201108117, 201407156, 202106166, 201911095, 201102270, 201103136, and 201102312. Tumor samples were collected during surgical resection and verified by standard pathology. GBM and ccRCC samples originating from the NCI CPTAC were part of the previous studies (Clark et al. 2019 and Wang et al. 2021). |

Note that full information on the approval of the study protocol must also be provided in the manuscript.

# Field-specific reporting

Please select the one below that is the best fit for your research. If you are not sure, read the appropriate sections before making your selection.

☒ Life sciences ☐ Behavioural & social sciences ☐ Ecological, evolutionary & environmental sciences

For a reference copy of the document with all sections, see nature.com/documents/nr-reporting-summary-flat.pdf

# Life sciences study design

All studies must disclose on these points even when the disclosure is negative.

| | |
|---|---|
| Sample size | Sample sizes were chosen based on the availability of samples collected across 11 cancer types. No specific statistical methods were used to predetermine sample size. However, our approach ensured a good representation for each cancer type, with a minimum of 10 samples per cancer type and a total of 225 samples for snATAC-seq, along with 206 samples for paired sc/snRNA-seq. This sample size aligns with or exceeds that of many previously published single cell chromatin accessibility studies on the date of submission. |
| Data exclusions | For the analyses results visualizations using averaged chromatin accessibilities in Fig. 1c and Extended Data Figs. 2g, 3a, 4a and 8a, cell groups with less than 20 cells were excluded as the averaged measurements per small cell number are less reliable. |
| Replication | We benchmarked accessible chromatin regions (ACRs) with the TCGA pan-cancer bulk ATAC-seq study (Corces et al. 2018), finding 8-27% of ACRs were shared between the bulk and our dataset, with the majority of ACRs being snATAC-unique (Supplementary Fig. 1a). We investigated these snATAC-unique ACRs and found 60-75% and 74-83% of them overlapped with regions identified by ENCODE ChIP-seq data (Dunham et al. 2012, Luo et al. 2020) and the fetal pan-organ snATAC-seq data (Domcke et al. 2020), respectively (Supplementary Fig. 1b-d). We also reasoned that single cell resolution could result in identification of non-cancer cell ACRs and indeed snATAC-unique ACRs were found in one or multiple cell types of tumor microenvironment (TME) (Supplementary Fig. 1e). We also observed that small proportions of snATAC-unique ACRs (ranging from 0.01% in ccRCC to 23.5% in CESC/AD) were cancer cell-unique (Supplementary Fig. 1e). Taken together, our snATAC-seq dataset provides a large number of reliable ACRs representing both cancer cell-specific and the TME-shared ACRs.<br><br>Tissue- and cancer cell-specific identified DACRs (differentially accessible chromatin regions) overlapped with cell type-specific regions previously identified in a snATAC-seq study of adult and fetal chromatin accessibility, as expected (Zhang et al. 2021; Supplementary Fig. 3).<br><br>To support our identified regulons, we looked into public databases and found that 41 out of 87 tissue- and cancer cell-specific transcription factors (TFs) were also differentially expressed in previous sc/snATAC-seq or bulk ATAC-seq studies, including BRCA (Kumegava et al. 2022), MM (Frede et al. 2021), ccRCC (Long et al. 2022), PDAC (Fan et al. 2022), pan-organ chromatin accessibility (Zhang et al. 2021), and the bulk ATAC-seq study in human cancers (Corces et al. 2018; Supplementary table 5a). We then validated the target genes for each TF using TF-specific ChIP-seq data from ENCODE (Dunham et al. 2012, Luo et al. 2020), corroborating direct binding to target genes in 51 out of 53 TFs that we examined (Extended Data Fig. 7a and Supplementary Table 5b-c). Our findings were further supported by a centered distribution of ChIP-seq peaks around TSSs of target genes, indicating TFs' regulation of the target genes. Furthermore, to validate our findings about tissue- and cancer cell-specific TFs, and metastasis associated TFs, we used results obtained by two orthogonal approaches: SCENIC to infer regulatory networks from sn/scRNA-seq data and chromVAR scores to infer TF activity based on their motif accessibility scores from snATAC-seq data. |
| Randomization | The study design didn't involve allocation of patients into treatment groups. Therefore randomization procedure was not relevant. |
| Blinding | The study design didn't involve allocation of patients into treatment groups. Therefore blinding procedure was not relevant. |

# Reporting for specific materials, systems and methods

We require information from authors about some types of materials, experimental systems and methods used in many studies. Here, indicate whether each material, system or method listed is relevant to your study. If you are not sure if a list item applies to your research, read the appropriate section before selecting a response.

## Materials & experimental systems

| n/a | Involved in the study | |
|---|---|---|
| ☐ | ☒ | Antibodies |
| ☐ | ☒ | Eukaryotic cell lines |
| ☒ | ☐ | Palaeontology and archaeology |
| ☐ | ☒ | Animals and other organisms |
| ☒ | ☐ | Clinical data |
| ☒ | ☐ | Dual use research of concern |
| ☒ | ☐ | Plants |

## Methods

| n/a | Involved in the study | |
|---|---|---|
| ☒ | ☐ | ChIP-seq |
| ☒ | ☐ | Flow cytometry |
| ☒ | ☐ | MRI-based neuroimaging |

# Antibodies

| | |
|---|---|
| Antibodies used | NRF1 (#46743; Cell Signalling Technology)<br>CTCF (#3418; Cell Signalling Technology)<br>GATA6 (#PA1-104, Invitrogen)<br>CK19 (#12434, Cell Signalling Technology) |
| Validation | Antibodies were validated by manufactures: NRF1 (#46743) and CTCF (#3418) have been validated using SimpleChIP Enzymatic Chromatin IP Kits; GATA6 (#PA1-104, Invitrogen) was verified by Cell treatment to ensure that the antibody binds to the antigen stated; CK19 (#12434, Cell Signalling Technology) was validated by Western blot, IHC and IF. |

# Eukaryotic cell lines

Policy information about cell lines and Sex and Gender in Research

| | |
|---|---|
| Cell line source(s) | Caki-1: ATCC catalog number HTB-46, https://www.atcc.org/products/htb-46<br>MCF7: ATCC catalog number HTB-22, https://www.atcc.org/products/htb-22<br>U251: from Luo et al. 2008 (PMID: 19091943) |
| Authentication | Caki-1 and MCF7 cell lines were authenticated via short tandem repeat (STR) profiling by ATCC. U251 cell line was authenticated via STR profiling. |
| Mycoplasma contamination | Caki-1, MCF7 and U251 tested negative for mycoplasma contamination using MycoAlert (Lonza, LT07-118). |
| Commonly misidentified lines (See ICLAC register) | None of the cell lines used here was among the commonly misidentified lines. |

# Animals and other research organisms

Policy information about studies involving animals; ARRIVE guidelines recommended for reporting animal research, and Sex and Gender in Research

| | |
|---|---|
| Laboratory animals | The following mouse strains (Mus musculus) were used as part of this study:<br>p48-Cre mice (C57BL/6J background), Laboratory of Sunil Hingorani<br>LSL-KrasG12D mice (C57BL/6J background), Jackson Laboratory #008179<br>Trp53flox mice (C57BL/6J background), Jackson Laboratory #008462 |
| Wild animals | No wild animals were used. |
| Reporting on sex | For all mouse experiments, cohorts were of equal numbers of each sex (both male and female) when possible. |
| Field-collected samples | No field collected samples were used. |
| Ethics oversight | All animal studies were completed in accordance with NIH-AALAC standards and consistent with Washington University School of Medicine IACUC regulations (protocol #22-0233), and studies were approved by Washington University School of Medicine Institutional Animal Studies Committee. All animals were housed in a barrier facility under a 12-hour light/dark cycle with 1-5 mice per cage. |

Note that full information on the approval of the study protocol must also be provided in the manuscript.
